# Safety and tolerability of losartan to treat recessive dystrophic epidermolysis bullosa in children (REFLECT): an open-label, single-arm, phase 1/2 trial

**DOI:** 10.1016/j.eclinm.2024.102900

**Published:** 2024-10-30

**Authors:** Dimitra Kiritsi, Franziska Schauer, Stella Gewert, Katja Reineker, Antonia Reimer-Taschenbrecker, Agnes Schwieger-Briel, Hagen Ott, Claudia Schmoor, Olga Grishina, Dedee Murrell, Brigitte Stiller, Tobias Zahn, Alexander Nyström, Leena Bruckner-Tuderman

**Affiliations:** a Department of Dermatology, Medical Center- University of Freiburg, Faculty of Medicine, University of Freiburg, Freiburg, Germany; b First Department of Dermatology, Faculty of Medicine, Aristotle University of Thessaloniki, Thessaloniki, Greece; c Department of Congenital Heart Disease and Pediatric Cardiology, University Heart Center Freiburg - Bad Krozingen, Medical Centre - University of Freiburg, Faculty of Medicine, Freiburg, Germany; d Pediatric Skin Center, Division of Dermatology, University Children's Hospital Zurich, Zurich, Switzerland; e Department of Pediatric Dermatology and Allergology, Children's Hospital Auf der Bult, Hannover, Germany; f Clinical Trials Unit, Faculty of Medicine, and Medical Center, University of Freiburg, Freiburg, Germany; g Department of Dermatology, St. George Hospital, Faculty of Medicine, University of NSW, UNSW Faculty of Medicine, Sydney, New South Wales, Australia; h Crowd Pharma GmbH, Pforzheim, Germany

**Keywords:** Genetic blistering disorder, Dystrophic epidermolysis bullosa, *COL7A1*, Symptom relief therapy, Skin fragility

## Abstract

**Background:**

Recessive dystrophic epidermolysis bullosa (RDEB) is a skin fragility disorder characterised by life-long mechanically induced skin blistering, fibrosis-driven pseudosyndactyly, and multi-organ involvement. Preclinical studies have suggested mitigated progression by angiotensin II type I receptor blockade through losartan. We aimed to determine the safety and tolerability of systemic losartan treatment among children with RDEB, and to obtain initial data on its clinical benefit.

**Methods:**

We conducted an open-label, single-arm, phase 1/2 trial at the Medical Center-University of Freiburg, Germany. Children with molecularly-confirmed RDEB, aged 2–16 years (starting from the 25th month of life) were eligible. Key exclusion criteria comprised anaemia with haemoglobin <8 g/dl; hypotension (defined as age-related systolic blood pressure under the 5th percentile); cardiologic contraindications, requirement for any medications that are likely to cause interactions with losartan; renal artery stenosis or renal insufficiency with creatinine clearance <30 ml/min; severe liver failure; severe, untreated electrolyte disturbances; history of cancer or chronic viral infections; hypersensitivity to losartan or any of the excipients and known or persistent abuse of medication, drugs, or alcohol. Treatment duration with losartan comprised 10 months, encompassing 16 weeks up-dosing of losartan, 24 weeks full dose losartan (final target dose of 1.4 mg/kg), and 4 weeks losartan tapering, followed by 12 weeks follow-up without losartan. The primary endpoint was occurrence of a serious safety concern, defined as one of the following side effects of losartan: clinically relevant severe hypotension, immediate hypersensitivity reactions to the drug or clinical relevant severe hypo- and hyperkalaemia. EB-specific scores (the EBDASI activity and damage score, Birmingham Epidermolysis Bullosa Severity Score (BEBS)) and other clinical outcome parameters were evaluated at five clinical visits as secondary outcomes: pain (Wong-Baker FACES Scale for pain), quality of life (Quality Of Life in EB [QOLEB] questionnaire and Children’s Dermatology Life Quality Index [CDLQI]), itch (Itch Assessment Scale for the Paediatric Burn Patients), dysphagia (Mayo Dysphagia Questionnaire-day 30 [MDQ-30]), pseudosyndactyly progression (our own morphometric scoring instrument), and hand function (Score of Colville and Terrill). All analyses (safety and efficacy) were performed in the safety population, defined as participants who received at least one dose of trial medication with losartan. This trial is registered with EudraCT, 2015-003670-32.

**Findings:**

Between Jul 28, 2017, and Feb 12, 2021, 29 children were enrolled. Of those 27 received the full treatment. Losartan was well tolerated, no treatment-related severe complications leading to a serious safety concern occurred. The patients revealed improvement in the RDEB clinical scores, namely a mean reduction at week 40 of −7.36 points (95%-CI: −16.13 to 1.41) in the EBDASI activity score and −10.50 points (95%-CI: −20.81 to −0.19) in the EBDASI damage score, while the Children’s Dermatology Life Quality Index rose by 2.64 points (95%-CI: −4.55 to −0.90). Similar to the EBDASI score, the BEBS showed a mean reduction of −3 points, 95%-CI: −0.21 to −5,79, P = 0.036). In the Wong-Baker FACES Scale for Pain an improvement of at least one level was identified for 9 of 28 patients between baseline and at month 9 (95%-CI: 15.9%–52.4%; P = 0.57). Regarding the Quality of Life in EB Score, five of 28 patients showed an improvement in the total scale of at least one level at month 9 (95%-CI: 6.1%–36.9%; P = 0.71). With the Itch assessment scale for the paediatric burn patients an improvement of at least one level could be observed in 12 of 28 patients (95%-CI: 24.5%–62.8%; P = 0.24). The MDQ-30 showed no relevant difference at 9 months after treatment start, as compared to baseline. We observed improvement of finger span with our own morphometric scoring instrument of pseudosyndactyly progression, revealing an increase of the maximal distance between thumb and index finger at month 9 by 6.92 mm, 95%-CI [3.48, 10.37] P = 0.0009. With the Hand function assessment score of Colville and Terrill, an improvement of at least one level was documented for 3 of 28 patients, i.e., 10.7% (95%-CI: 2.3%–28.2%; P = 0.63).

**Interpretation:**

Our results suggest that losartan was well tolerated by children with RDEB, and provide preliminary evidence that it may reduce disease burden. Further research with larger sample sizes and longer durations is needed to establish the treatment's long-term efficacy and safety.

**Funding:**

10.13039/501100011960Debra International, the Department of Dermatology, Medical Center-10.13039/501100002714University of Freiburg (Berta-Ottenstein Advanced Clinician Scientist Program of the Medical Faculty), and the 10.13039/501100001659German Research Foundation.

**Research in context**

**Evidence before this study**

Before the study, we searched PubMed for publication in English up until 2016/06/30 using the search terms “dystrophic epidermolysis bullosa” and “clinical trial” and “losartan”. This yielded no results. Based on our preclinical study, using losartan in RDEB mice this trial was initiated.

**Added value of this study**

Herein present an open-label, single-arm, phase 1/2 trial to determine safety and tolerability of systemic losartan treatment among children with RDEB, and to obtain initial data on its clinical benefit. Children with RDEB received oral losartan once daily for 9 months, followed by 3 months follow-up without losartan. To our knowledge this is the largest trial with a systemically applied treatment for patients with RDEB, acting as a disease-modifying therapy for RDEB. We have applied a variety of clinical scores showing improvement within the treatment period of 9 months and also provide data regarding inflammation and fibrosis, based on markers in patient sera and skin biopsies. Specifically, the patients revealed improvement in the RDEB clinical scores, while the Children’s Dermatology Life Quality Index rose. We also observed improvement in finger span. Additionally, several inflammation markers in the patients’ blood either remained stable or decreased, unlike data from natural history studies. Specifically, TNF decreased significantly, as also did marker of fibrosis evaluated in skin biopsies (as is picrosirius red). The drug was well tolerated with a safety profile consistent with the label for its use in hypertension.

**Implications of all the available evidence**

The landscape of therapeutics in RDEB is rapidly evolving. Anti-inflammatory and/or antifibrotic treatments might be beneficial to improve the RDEB systemic involvement and enhance the efficacy of topical curative approaches. We show that the widely available losartan is a safe and efficient symptom-relief therapy for RDEB and provide evidence in the largest cohort treated with losartan so far. This pathway has already been shown to be involved in the pathogenesis of another genetic disorder, the Marfan syndrome, where usefulness of losartan has just been reported in a meta-analysis of 1442 patients. For RDEB, 3 smaller studies with a total of 11 patients have been published with different dosages of losartan for a few weeks, all showing improvement of specific disease features (e.g., wound healing, total skin involvement or quality of life). In addition, we present outcome measures and instruments that could be used for trials on systemic treatments for RDEB in the future.

## Introduction

Recessive dystrophic epidermolysis bullosa (RDEB) is a progressive inherited blistering skin disease caused by collagen VII deficiency, an extracellular matrix protein and main constituent of the anchoring fibrils that are required for stable dermal to epidermal adhesion.^1^ Progressing RDEB follows a trajectory of unremitting skin blistering extending to development of a severely debilitating injury- and inflammation-driven disease associated with chronic wounds and heavy fibrosis, which manifest on hands and feet as pseudosyndactyly.^2^ The mucosae are also affected, involving severe dysphagia due to oral and esophageal involvement.^3^ Chronic itch and pain significantly limit the patients’ quality of life.^3^ RDEB is a systemic disease; however, most of the progress in developing clinical therapies has been made on topical approaches, and the only EMA approved drug for EB so-far, Filsuvez® gel, is a topical wound treatment. There is an urgent unmet need for safe treatments limiting RDEB’s systemic disease advancement.

Our investigations on cellular and molecular disease progression in RDEB identified inflammation and heightened transforming growth factor β (TGFβ) activity as major mechanisms downstream of the tissue fragility driving RDEB progression.^4^^,^^5^ Losartan, an angiotensin II type 1 receptor (AT1R) antagonist, has been a well-established medication since 1995 to treat hypertension and other conditions in children and adults alike with a benign safety profile in both age groups. On the tissue level, signalling through AT1R is connected to pro-inflammation and heightened transforming growth factor β (TGFβ) activity, which are two main pathways furthering fibrosis in a destabilised microenvironment.^6^ Thus, losartan appeared as a suitable drug candidate for RDEB. In a pre-clinical study using RDEB donor-derived *in vitro* models and a murine genetic model of RDEB, we observed that systemically administered losartan reduced inflammation and the ensuing fibrosis, thereby greatly alleviating symptoms and attenuating the advancement of RDEB.^2^ These data led us to hypothesize that repurposing losartan for RDEB would constitute a safe and effective approach to prevent systemic disease progression in children with RDEB. The primary objective was to determine the safety and tolerability of losartan for treatment of children with RDEB and secondary to obtain initial data on its clinical benefit.

## Methods

### Study design

The reporting adhered to the CONSORT guidelines. The investigator-initiated phase 1/2 trial REFLECT (symptom-RElieF with Losartan—Eb Clinical Trial) was designed by investigators at the Department of Dermatology, Medical Center-University of Freiburg and was financed by the patient organisation DEBRA International. Data analysis was performed by the Clinical Trials Unit, Medical Center- University of Freiburg. The trial protocol (Supplemental material) was approved by the Ethics committees of the University of Freiburg (number 157/16, EudraCT Number: 2015-003670-32, REFLECT trial) and the national competent authority BfArM. All patients provided their assent and their legal guardians provided written informed consent. No changes were made in the study design after approval of the protocol and trial commencement. The study medication comprised losartan potassium, which was prepared as extemporaneous oral solution at a concentration of 2.5 mg/ml by the pharmacy of the University Medical Center of the Johannes Gutenberg-University Mainz.^7^ All data were collected during planned visits to the Medical Center- University of Freiburg.

In this single-arm study, treatment duration with losartan comprised 10 months, encompassing 16 weeks up-dosing of losartan, 24 weeks full dose losartan (final target dose of 1.4 mg/kg), and 4 weeks losartan tapering, followed by 12 weeks follow-up without losartan. The primary objective of the present trial was to investigate losartan’s safety and tolerability in moderate to severe RDEB. A secondary objective was to deliver initial evidence on the efficacy of losartan in alleviating the disease manifestations and raising quality of life, and reducing inflammation and fibrosis in moderate to severe RDEB. Efficacy was assessed at week 40 (the end of full-dose losartan treatment), in comparison to the patients’ own baseline values before starting treatment. Based on available data on the natural history of DEB,^8^^,^^9^ the baseline was regarded as adequate control at this stage, since a spontaneous alleviation of inflammation, fibrosis, and mitten deformities cannot be expected. We therefore considered lowering of the disease scores as treatment success.

### Participants

Children (age 2–16 years) with a molecularly confirmed diagnosis of intermediate to severe RDEB were recruited into the trial. To be eligible, they could not have suffered from severe anaemia with haemoglobin below 8 g/dl or hypotension with an age-related systolic blood pressure under the fifth percentile. Cardiologic contraindication or medications likely to cause interactions with losartan were also excluded. Further exclusion criteria comprised renal artery stenosis or renal insufficiency with creatinine clearance <30 ml/min; severe liver failure; severe, untreated electrolyte disturbances; history of cancer or chronic viral infections; hypersensitivity to losartan or any of the excipients and known or persistent abuse of medication, drugs, or alcohol.

### Randomisation and masking

This is a single-arm study, thus no randomisation and masking were performed. All participants received the study drug.

### Procedures and outcomes

Adverse events (AEs) and serious adverse events (SAEs) were recorded by monitoring heart rate and function and blood pressure, via echocardiography, home blood pressure monitoring devices, and blood tests. All AEs were coded using the Medical Dictionary for Regulatory Activities (MedDRA), version 23.0. A patient diary was provided, and heart rate and blood pressure were measured and recorded daily. Laboratory tests were performed according to clinical practice, comprising blood electrolytes, urea, creatinine, aspartate aminotransferase (GOT), alanine aminotransferase (GPT), lactate dehydrogenase (LDH), cholinesterase, full cell blood count, NT-proBNP, C-reactive protein (CRP), and ferritin. The primary endpoint was any occurrence of a severe complication leading to a serious safety concern, defined as any of these losartan side effects: clinically relevant severe hypotension, immediate hypersensitivity reactions to the drug, or clinically relevant severe hypo- or hyperkaelemia.

Efficacy was assessed applying validated scoring systems for the clinical manifestations of RDEB, comprising the Birmingham Epidermolysis Bullosa Severity Score (BEBS),^10^ the Epidermolysis Bullosa Disease Activity and Scarring Index (EBDASI),^11^ the hand function Assessment score of Colville and Terrill as well as an own morphometric instrument to score pseudosyndactyly progression (based on already published data in^2^), the Mayo Dysphagia Questionnaire-day 30 (MDQ-30), an Itch Assessment Scale for the Paediatric Burn Patient, the Wong-Baker FACES Scale for Pain and two quality of life questionnaires (the Quality Of Life in EB questionnaire—QOLEB,^12^ primarily completed by the parents and Children’s Dermatology Life Quality Index- CDLQI,^13^ primarily completed by the children). Children’s height and weight were assessed at every visit; sex- and age-specific percentile values were derived from EB-specific growth charts of a group of 157 untreated children with RDEB serving as natural history control.^8^ Outcome measurements were not changed during the trial and conducted according to the study protocol established and approved before the commencement of the trial.

Efficacy was further assessed using fibrotic and inflammatory markers in skin (consent to a skin biopsy was provided by 7 of the 29 patients) and the patients’ sera (see protocol in the Supplemental material).

### Statistical analysis

Sample size calculation was based on the primary endpoint occurrence of a serious safety concern. A total of 29 trial participants was considered appropriate. If none of the 29 participants developed a severe complication leading to a serious safety concern, we concluded that with 95% probability, the severe complication rate is below 10% (upper limit of the 90% confidence interval (CI) equal to 9.8%). Incidences of AEs and SAEs were reported with 95%-CI. Efficacy endpoints were analysed by comparing post-treatment measurements after 40 weeks with pre-treatment measurements, and by calculating 95% confidence intervals for the differences based on normal distribution (for some parameters after log-transformation) for continuous data, or based on the binomial distribution for binary response data. Corresponding descriptive statistical tests comparing within-patient measurements were performed without adjustment for multiplicity in statistical tests and calculation of CI regarding secondary endpoints. The following statistical tests were performed: For continuous data for which a normal distribution could be assumed (for some parameters after log[1] transformation) paired t-test was used; for continuous data for which a normal distribution could not be assumed paired Wilcoxon test was used; and for binary data the McNemar test was used. Statistical tests were performed at a significance level of 0.05, but their results were interpreted in a descriptive sense. All analyses were performed in the safety population including all patients given at least one dose of losartan. Exploratory subgroup analyses were performed (subgroups defined by sex and RDEB severity (moderate versus severe).

### Role of the funding source

The funder of the study had no role in study design, data collection, data analysis, data interpretation, or writing of the report. DK, AN and LBT had access to the full dataset and had final responsibility for the decision to submit for publication.

## Results

### Characteristics of patients receiving losartan therapy

**Fig. 1:**
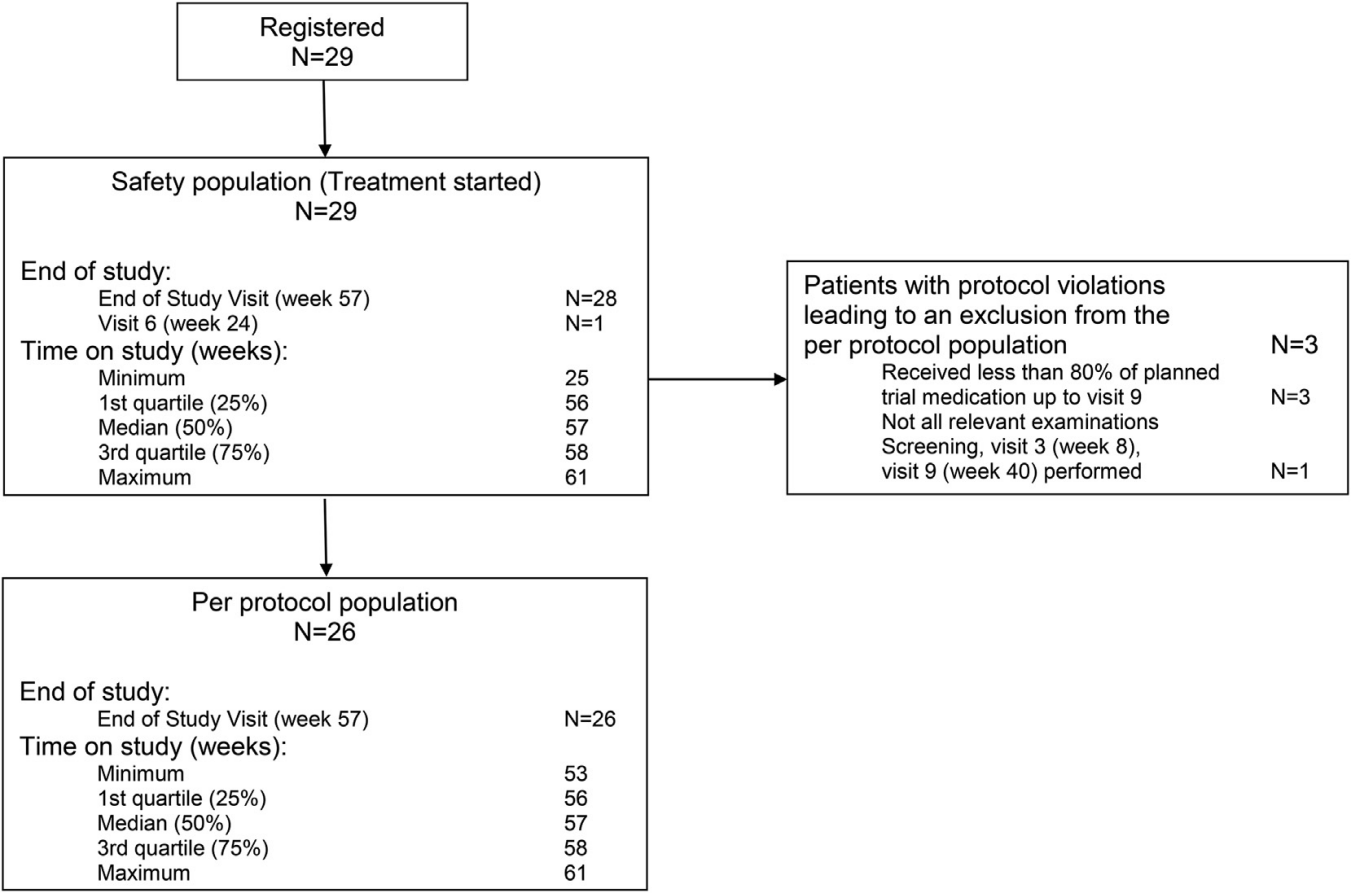
**Trial profile.** 29 children with RDEB were registered in the REFLECT trial, while 26 received more than 80% of the planned trial medication and had all relevant examinations done at visit 9.

The 29 patients enrolled in this trial were from 2 to 14 years old (between 25 and 160 months; specifically up to 5 years: 12 patients, between 5 and 10 years: 11 patients and over 10 years: 6 patients), while 55.2% of the participants were male and 44.8% female. Sixteen (55.2%) patients had severe RDEB, while 13 revealed moderate severity (44.8%). The majority (82.8%) had experienced at least one previous and/or ongoing disease or interventions, most frequently involving ‘gastrointestinal tract’ (48.3%), ‘surgery’ (34.5%), and ‘dermatologic, other than RDEB’ (17.2%), which could all be linked to RDEB. All children were given at baseline at least one concomitant medication, including vitamin substitutions. Baseline characteristics are shown in Table 1 and the trial profile is depicted in Fig. 1.

**Table 1:** Baseline characteristics.

Baseline characteristics	N = 29
Age; median (min; max), years	6 (2; 14)
Sex	
Female	13
Male	16
Weight; median (min; max), kg	16.6 (9.9; 50.9)
Height; median (min; max), cm	109 (83; 159)
Disease severity	
Severe RDEB	16
Moderate RDEB	13
Birmingham Epidermolysis Bullosa Severity Score; median (min; max)	25 (5; 45)
Epidermolysis Bullosa Activity and Scarring Index, Total; median (min; max)	171 (47; 251)
Epidermolysis Bullosa Activity and Scarring Index, Total Activity; median (min; max)	45 (19; 88)
Epidermolysis Bullosa Activity and Scarring Index, Total Damage; median (min; max)	124 (28; 200)
Hand function score by Colville and Terill	
Grad 0: no fusion	22
Grad 1: fusion extending to the proximal interphalangeal joint	5
Grad 2: fusion extending to the distal interphalangeal joint of finger	0
Grad 3: fusion extending to the tip of the digit	2
Children’s Dermatology Life Quality Index; median (min; max)	11 (2; 22)
Itch, as per Assessment Scale for the Paediatric Burn Patients	
Comfortable, no itch	0
Itches a little, does not interfere with activity	7
Itches more, sometimes interferes with activity	7
Itches a lot, difficult to be still, concentrate	12
Itches most terribly, impossible to sit still	3
Pain, as per Wong-Baker FACES Scale for pain	
No Hurt	7
Hurts little bit	11
Hurts little more	5
Hurts even more	3
Hurts whole lot	3
Hurts worst	0
Quality Of Life in EB (QOLEB) questionnaire, Total Scale; median (min; max)	20 (1; 36)
Mayo Dysphagia Questionnaire-day 30 (MDQ-30); median (min; max)	15 (0; 65)

### Dose escalations, safety and tolerability

**Fig. 2:**
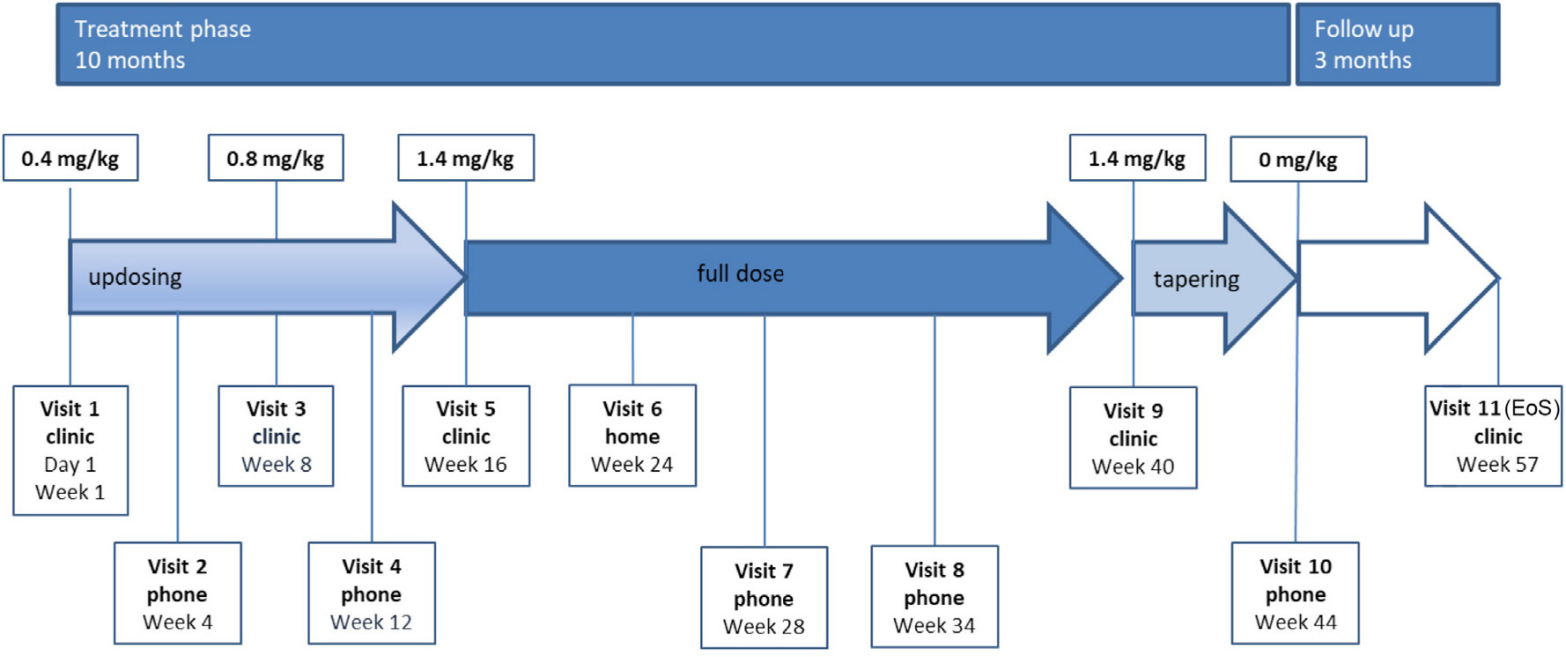
**Trial design.** Children with moderate to severe RDEB received losartan at a starting dose of 0.4 mg/kg bw, followed by an updosing to 1.4 mg/kg bw maintained for 5 months. Losartan was then tapered off within one month, with a subsequent 3 months follow up. The respective losartan dose and the visits, as well as their nature, are indicated in the graph.

The treatment period lasted 10 months entailing an escalating dose of losartan (starting with 0.4 and up to 1.4 mg/kg), followed by 3 months follow-up without losartan (Fig. 2). Six patients experienced at least one dose change/interruption of losartan administration, in all cases due to AEs. Two patients prematurely terminated the study: one developed peritonitis after leakage in the gastrostoma and died several weeks after losartan discontinuation of multiorgan failure; the other withdrew consent and refused the final visit because of fearing a COVID-19 infection. Three patients received less than 80% of the planned trial medication, one being the aforementioned deceased patient. The second suffered diarrhea that developed after the first dose increase, thus we kept the losartan at a lower dose throughout the whole study, while the third patient had two episodes of skin infections requiring intravenous antibiotics (losartan was discontinued only during that time).

Twenty-five (86.2%, 95%-CI: 68.3–96.1) patients suffered at least one adverse event, 14 (48.3%, 95%-CI: 29.4–67.5) had at least one infection, to be expected in a paediatric population (Table 2). One patient’s itching was the only AE considered related to the study medication. We observed no blood-pressure anomalies during the trial (median systolic blood pressure at baseline 101 mm Hg and at week 40 it was 102 mm Hg; median diastolic blood pressure at baseline 67 mm Hg and at week 40 61 mm Hg; median heart rate at baseline 109/min and at week 40 it was 110/min). No abnormalities in the electrocardiogram or echocardiography were documented while the patients received losartan. In fact, altogether, no safety concerns were raised, particularly no adverse events or severe complications leading to a serious safety concern. We can therefore conclude that with 95% probability the severe complication rate is below 10%.

**Table 2:** Incidence of Adverse Events (AEs) (occurring in ≥5% of patients) by MedDRA System organ class and preferred terms.

System organ class	Preferred term	AE Incidence		
		N	%	95%-Confidence Interval (%)
Total number of patients		29	100.0	
Number of patients with at least one AE		25	86.2	68.3–96.1
Infections and infestations		14	48.3	29.4–67.5
	Nasopharyngitis	5	17.2	5.8–35.8
	Conjunctivitis	2	6.9	0.8–22.8
	Urinary tract infection	2	6.9	0.8–22.8
	Wound infection	2	6.9	0.8–22.8
Gastrointestinal disorders		9	31.0	15.3–50.8
	Nausea	3	10.3	2.2–27.4
	Diarrhea	2	6.9	0.8–22.8
	Dysphagia	2	6.9	0.8–22.8
	Vomiting	2	6.9	0.8–22.8
General disorders and administration site conditions		6	20.7	8.0–39.7
	Pyrexia	2	6.9	0.8–22.8
	Temperature intolerance	2	6.9	0.8–22.8
Skin and subcutaneous tissue disorders		5	17.2	5.8–35.8
	Pruritus	3	10.3	2.2–27.4
Investigations		3	10.3	2.2–27.4
	Body temperature increased	2	6.9	0.8–22.8
Nervous system disorders		3	10.3	2.2–27.4
	Headache	3	10.3	2.2–27.4
Respiratory, thoracic and mediastinal disorders		3	10.3	2.2–27.4
	Epistaxis	2	6.9	0.8–22.8
Blood and lymphatic system disorders		2	6.9	0.8–22.8
	Anaemia	2	6.9	0.8–22.8
Injury, poisoning and procedural complications		2	6.9	0.8–22.8

Regarding the laboratory parameters: in general, changes from baseline to week 40 were small. For ferritin, an increase to week 40 was observed with a ratio to baseline of 1.46 (95%-CI: 1.15–1.83), P = 0.002. Serum CRP did not increase to week 40 with a ratio to baseline of 1.15 (95%-CI: 0.82–1.63), P = 0.39 (Supplemental Table S1). This indicates no progressive increase in systemic inflammation with age, as has previously been reported for RDEB.^8^

### Response evaluation

**Fig. 3:**
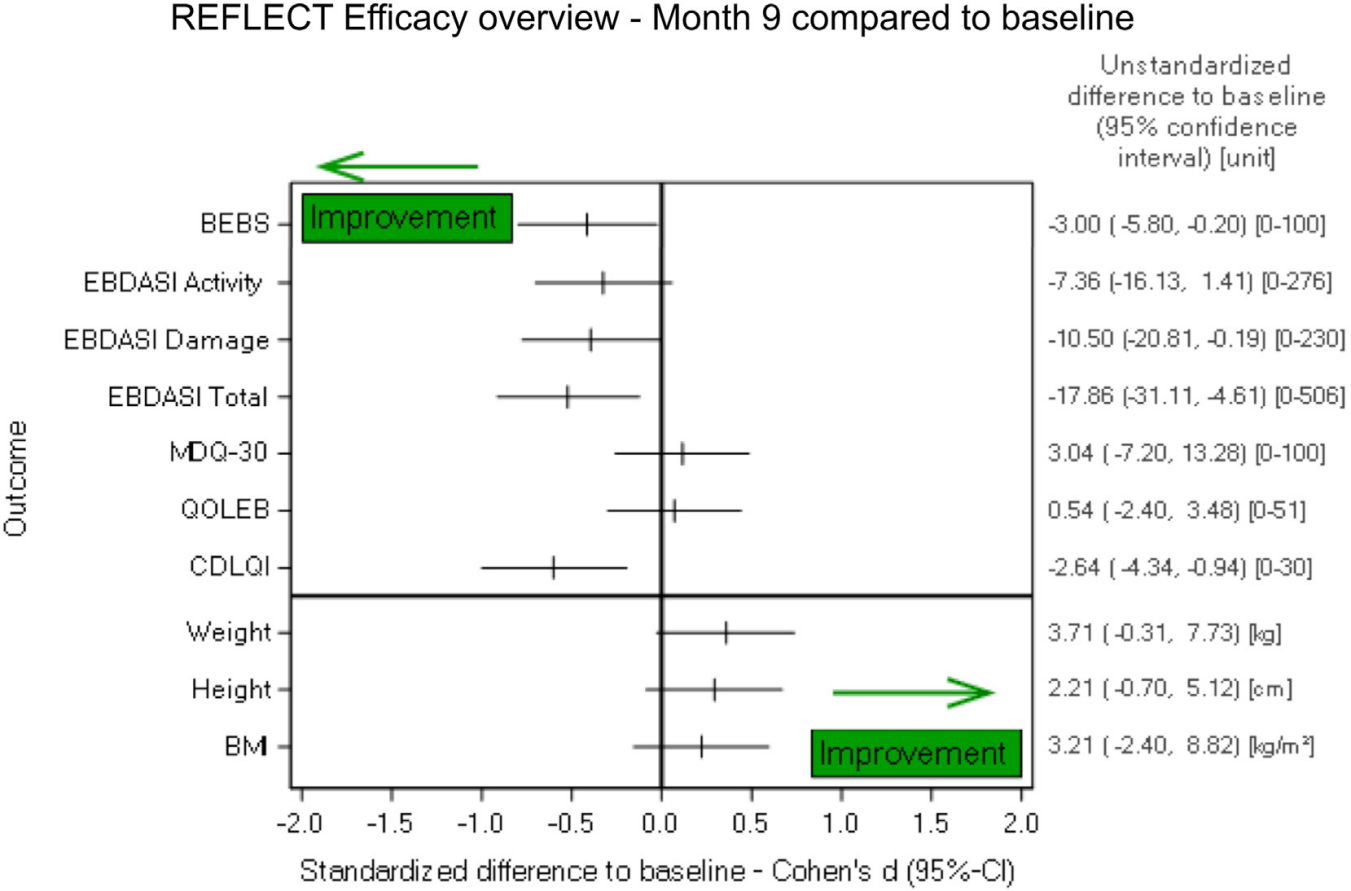
**Assessment of clinical benefit.** Most patients had some degree of improvement in the different functional, patient-reported and observed reported outcome measures, used to assess the manifestations of RDEB after 9 months of losartan treatment, when compared to the baseline. Only 2 scores remained unchanged or slightly worsened. For weight and height in the graph only the changes to baseline are presented, but for statistical analysis a historical untreated control group was employed, as detailed in the manuscript. BMI, body mass index; CDLQI, Children’s Dermatology Life Quality Index; QOLEB, Quality Of Life in EB questionnaire; MDQ-30, Mayo Dysphagia Questionnaire-day 30; EBDASI, Epidermolysis Bullosa Disease Activity and Scarring Index; PGA, Physician Global Assessment; BEBS, Birmingham EB severity score.

**Fig. 4:**
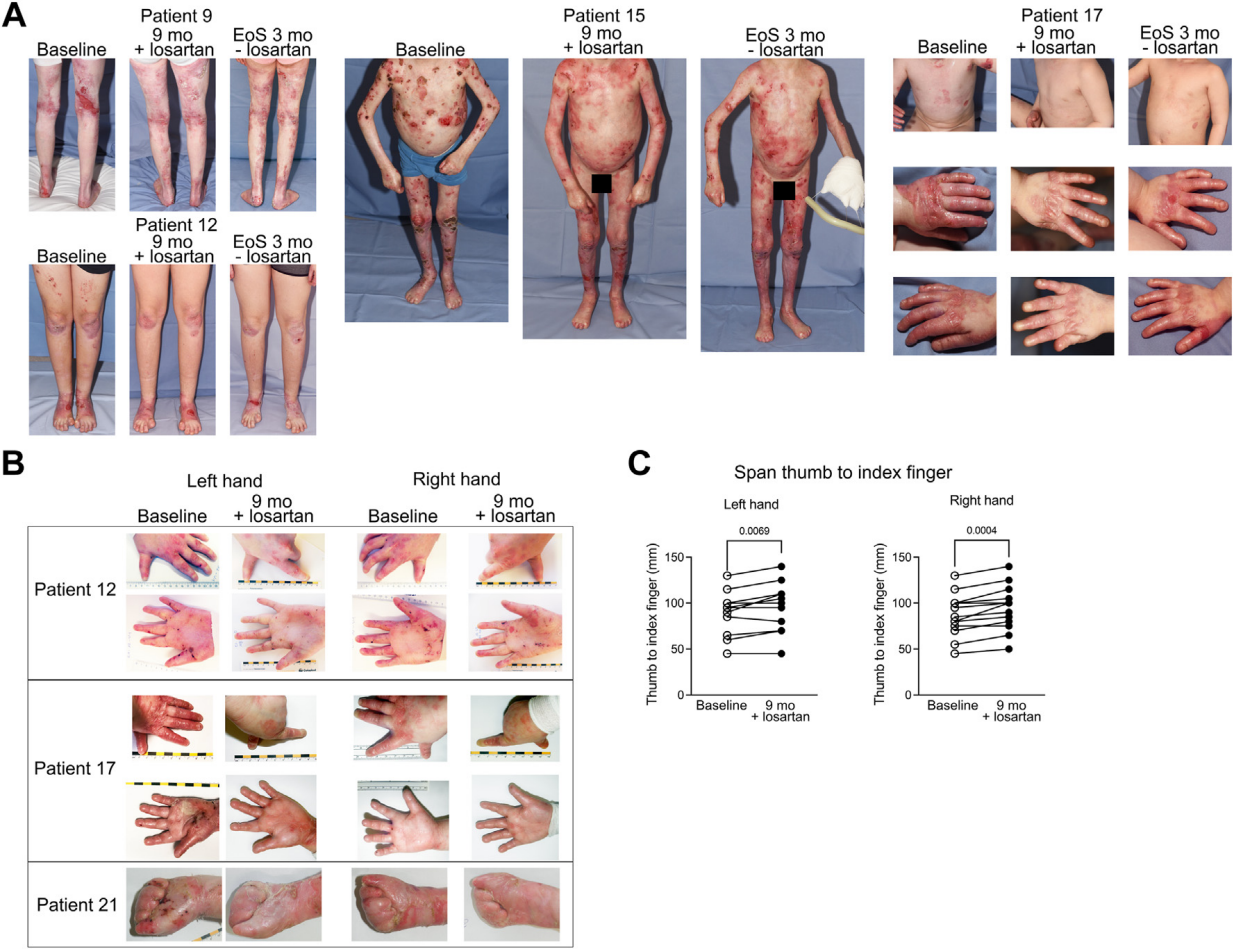
**Examples of specific patients and results of the morphometric hand function evaluation.** Panel A depicts body areas of representative patients with different ages and RDEB severity, prior to (visit 1) and after 9 months (visit 9) of losartan treatment. In general, less erythema and fewer wounds are present after losartan. Panel B and C present the hand functionality score used at visit 1 and 9. The plots show thumb to index finger span of left and right hands, respectively at visit 1 (open circles) and 9 (closed circles). All patients show a stabilisation or increase in the finger span, which is more significant on the right hand. P values were obtained with the use of Student’s t test.

The EBDASI represents the most widely used physician assessment score for EB. EBDASI assesses response to therapy separately from chronic damage, and is thus a suitable scoring system for interventional studies. A drop of at least 9 points in the Activity score is regarded as clinically meaningful,^11^ and 13 of 28 patients (46.4%; 95%-CI: 27.5%–66.1%) revealed this clinically relevant reduction in the relatively short 40-week treatment period. Mean change was −7.36 (95%-CI: −16.13 to 1.41), P = 0.09. There was no heterogeneity observed in the EBDASI score in female versus male patients, but appeared to be in the moderate versus severely affected patients. Specifically, it dropped from 48.69 to 37.63 in the severely affected patients (−11.06 points, 95%-CI: −21.73 to −0.39, P = 0.04), while in those with moderate disease the mean EBDASI activity score fell from 37.67–35.25 points at week 40 (−2.42 points, 95%-CI: −18.76 to 13.93, P = 0.75). The EBDASI damage score showed a reduction of −10.50 points (95%-CI: −20.81 to −0.19), P = 0.04, with no major difference in patients with moderate versus severe RDEB. The EBDASI total score showed a reduction of −17.86 points (95%-CI: −31.11 to −4.61), P = 0.01, with a larger reduction observed in severe RDEB (mean −22.13 points, 95%-CI: −41.36 to −2.89, P = 0.02) versus moderate RDEB (mean −12.1795%-CI: −35.52 to 8.19, P = 0.21) (Fig. 3). Examples of patients exhibiting clinically meaningful improvement in skin involvement with fewer skin lesions and less erythema are shown in Fig. 4A. Gradual worsening of the skin condition was observed after losartan withdrawal at the end of study visit. Pictures of the same patients from Fig. 4 are depicted 3 months after discontinuation of losartan in the Supplemental Fig. S1.

Other tested endpoints with conforming positive data included the Birmingham EB severity score (BEBS), patient-reported pain, itch and quality of life (Children’s Dermatology Life Quality Index, CDLQI), and a morphometric scoring instrument of pseudosyndactyly progression. Similar to the EBDASI score, the BEBS (assessed in 28 patients) suggested a more prominent losartan effect in severe RDEB than in moderate RDEB (in severe RDEB reduction of −4.06 points (95%-CI: −8.30 to 0.18, P = 0.05) from initially mean 28.94, while in moderate RDEB reduction of −1.58 points (95%-CI: −5.47 to 2.30, P = 0.38) from initially mean 18.33; for the totality of patients mean reduction of −3 points, 95%-CI: −0.21 to −5,79, P = 0.036). A specifically interesting observation we made was improved sleep. As evaluated in the CDLQI (assessed in 28 patients), sleep improved in 12 patients, but was worse in 3 children (P = 0.01) (Fig. 3).

The analysis of the changes in the Wong-Baker FACES Scale for Pain (ranging from 0 = best to 10 = worst) between baseline and at month 9 revealed an improvement of at least one level for 9 of 28 patients, i.e., 32.1% (95%-CI: 15.9%–52.4%; P = 0.57). The change in the total score (0 = best, 51 = worst) of the Quality of Life in EB (QOLEB) during the study was assessed by the parents of the children. At month 9, five of 28 patients showed an improvement in the total scale of at least one level i.e., 17.9% (95%-CI: 6.1%–36.9%; P = 0.71). The comparison of the Itch assessment scale for the paediatric burn patients, ranging from 0 = best to 4 = worst, between baseline and month 9 showed an improvement in nearly half of the patients. After 9 months of treatment, an improvement of at least one level could be observed in 12 of 28 patients, i.e., 42.9% (95%-CI: 24.5%–62.8%; P = 0.24). The MDQ-30 was used to assess oesophageal involvement. This validated 28-item tool measures oesophageal dysphagia within the last 30 days before the visit and assesses improvement of swallowing and eating during treatment. Overall, no relevant difference was observed at 9 months after treatment start, as compared to baseline. Looking on the categories of the dysphagia score (0–15 = negative, 16–39 = indeterminate, 40–100 = positive), 5 of 28 patients, i.e., 17.9% (95%-CI: 6.1%–36.9%; P = 0.55) showed an improvement of at least one level.

Using a historical untreated control group for comparison,^8^ sex- and age-specific weight and height data showed greater growth under losartan treatment–highly relevant outcome in this patient population (weight percentiles increased from mean 57.3 at baseline to mean 61.1 at week 40, difference 3.71, 95%-CI: −0.31 to 7.74, P = 0.06; height percentiles rose from mean 49.00 to 51.21, difference 2.21, 95%-CI: −0.70 to 5.13, P = 0.13). Finally, we assessed losartan’s fibrosis-modulating effect via a morphometric system measuring the change in the span between the thumb and index finger (assessed in 13 children, due to challenges in compliance with smaller children). The children demonstrated the ability to span their hands better after 40 weeks of losartan (right hand: 11 patients showed longer span, while 2 remained stable; left hand: 8 patients had longer span, 4 remained stable and one worsened), as Fig. 4B and C illustrate. Specifically, with ratios to baseline of 0.98, 0.94, 95%-CI [0.88, 1.01, P = 0.11], at month 9 the shortening of the index and middle fingers was only mild. Regarding the maximal distance between thumb and index finger a slight improvement could be observed at month 9, i.e., it was increased by 6.92 mm, 95%-CI [3.48, 10.37] P = 0.0009, from a mean value of 87.12 mm–94.04 mm. The comparison of the Hand function assessment score of Colville and Terrill, ranging from 0 = best to 3 = worst, between baseline and the post-treatment time point month 9 revealed no change in most of the patients. An improvement of at least one level was documented for 3 of 28 patients, i.e., 10.7% (95%-CI: 2.3%–28.2%; P = 0.63). The interpretation of this result hast to take into account that 22 patients were at level 0 (no fusion) at baseline, so an improvement on this scale was not possible.

**Fig. 5:**
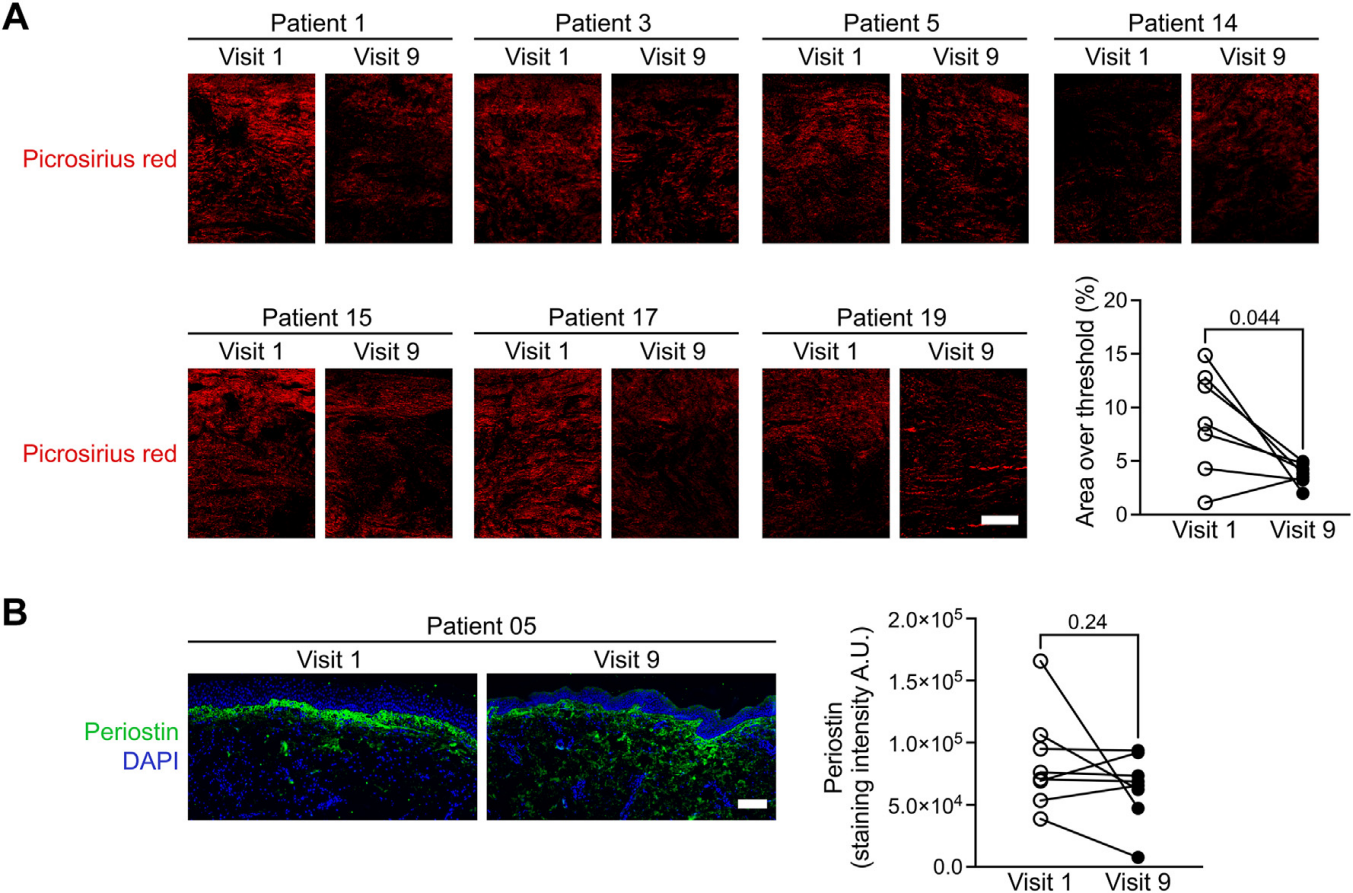
**Analyses of skin fibrosis markers.** Sections from 7 children with moderate to severe RDEB were stained with picrosirius red (A) and antibodies against periostin (B). Picrosirius red was visualised under polarising light, while for periostin fluorescence microscopy was used (scale bar, 100 μm). A decrease in both stainings is observed at the skin biopsies taken after losartan treatment. P values were obtained with the use of Student’s t test.

Additionally, several inflammation markers in the patients’ blood either remained stable or decreased, unlike data from natural history studies.^8^^,^^9^ Specifically, TNF decreased from mean 14.83 at baseline to mean 13.54 at week 40 (ratio 0.89, 95%-CI: 0.80–0.99, P = 0.04) (Supplemental Table S2). We analysed several markers for inflammation and fibrosis in skin in a smaller number of patients who had provided skin biopsies. Most strikingly, we observed a reduced picrosirius red staining in 6/7 patients, indicating ameliorated extracellular matrix remodelling in RDEB skin (Fig. 5A–B). Similarly, staining of periostin, a marker linked to the tissue remodelling (which is highly expressed under fibrotic conditions and scleroderma),^14^ was also reduced in 6/7 patients (Fig. 5C). Staining for immune cells in the skin showed no changes in CD68, CD4 or CD8 cells. We detected an increase in the CD45RO+ (peripheral memory T cells)/CD45RA+ (effector T cells) ratio in treated DEB skin (Supplemental Fig. S2). A lower ratio of these markers is known to be a determinant of poor wound healing in DEB.^15^

## Discussion

Because of its anti-inflammatory and anti-fibrotic role, losartan has been tested as a symptom-relief treatment for rare disorders including focal segmental glomerulosclerosis,^16^ renal interstitial fibrosis,^17^ Marfan syndrome,^18^ Ehlers Danlos syndrome,^19^ Alport syndrome,^20^ liver fibrosis^21^ and others. In topical formulations use of angiotensin II receptor inhibitors ensued improvement of hypertrophic scars^22^ and chronic wounds.^23^ In general, the results have varied, since the biological effects of AT1R interference are strongly context- and disease-dependent. That is why its inhibition must be tested for each disease separately. Given the previous work on losartan in paediatric populations, its safety profile and the existing clear recommendations for use in children, we chose it over other potential AT1Rs for the initial preclinical studies^2^ and subsequently for the here presented clinical trial.

In case of RDEB, preclinical research involving a collagen VII-hypomorphic, RDEB mouse model uncovered injury and inflammation-associated damage response in supporting progression of the fibrotic phenotypes,^2^ which, including formation of pseudosyndactylies, could be effectively prevented through oral losartan.^2^ These studies also suggested that already established fibrosis cannot be sufficiently reversed.^2^ Therefore, the earlier the treatment starts, the more likely we are to inhibit advancing disease manifestations. This was our rationale for conducting a trial with losartan on children with RDEB.

The REFLECT trial provides evidence that losartan is safe and well tolerated in children with moderate to severe RDEB, revealing no detrimental impacts on blood pressure and cardiac health, while losartan consistently showed beneficial effects in several efficacy outcome parameters. Although due to the study design a placebo group was not included, we nevertheless compared several efficacy parameters to historical natural history data, which demonstrated progressing RDEB signs and symptoms.^8^ Similarly, initial data on EBDASI scores’ evolution over time showed that such symptoms do not improve spontaneously.^24^ Contrastingly, losartan disrupted progressive increase of many such parameters or even improved clinical scores used to assess the RDEB burden, including relevant preservation of hand functionality. Our data indicate that losartan is able to attenuate fibrosis or even to some extent reverse it. Thus, it holds the promise to delay formation of mitten deformities.

Losartan is not a curative treatment for RDEB, but it promises substantial benefits as a disease-modifying therapy. Importantly it is a systemic treatment. Our trial data highlight that systemic losartan may prevent disease progression, reduce inflammation, and improve the clinical picture. In line with this evidence, three pilot case reports observed improved skin involvement and higher quality of life scores with losartan.^25^, ^26^, ^27^ Unlike those small studies involving both adults and children, we evaluated losartan’s effects only in children presenting a molecular RDEB diagnosis, in order to observe its effects on disease progression. Our reliance on a larger, uniform patient population demonstrated improved hand-function scores, thus indicating slower progress in soft tissue fibrosis of the hands and better function during losartan treatment. On the other hand, as we recruited only children, the baseline scores were lower in the scoring systems assessing cutaneous and extracutaneous manifestation in RDEB (baseline median score: EBDASI total 171, BEBS 25), thus restricting the potential improvement likely originating from the treatment, and making the improvements in EBDASI and BEBS scores we observed all the more remarkable.

A study limitation is the absence of a control group, and the open-label design carrying the inherent risk of observer bias in outcome assessments. However, the remarkable improvements in investigator-reported outcomes, especially EBDASI and BEBS, are reinforced by our results from objective, quantitative measurement tools such as the hand morphometric scoring tool, bodyweight and height, all of which revealed improvements unlike natural history data.^8^

The benefit of targeting the renin-angiotensin system is that we target multiple pathways dysregulated in DEB by limiting TGFβ, inflammation and fibroblast activation. Following this, given that the renin-angiotensin system simplified has 2 axes: one pro-fibrotic axis mediated by angiotensin II type 1 receptor and one fibrosis-limiting axis mediated by the angiotensin II type 2 receptor, MAS receptors, we are convinced that a wider approach is safer and more efficacious than targeting single pathways. Based on our previous work in Bernasconi et al.,^4^ we believe that using angiotensin type II receptor 1 blockers is more efficacious than using angiotensin converting enzyme in inhibitors, as the anti-fibrotic axis is maintained. Losartan also holds promise for combination therapy when taking curative approaches, since by preventing inflammation and fibrosis it improves the tissue microenvironment, thereby facilitating the take and sustainability of gene-, protein- and cell-based therapeutics. Developing sustainable, efficacious therapies for RDEB have proven to be challenging. Different approaches to replace missing or non-functional collagen VII have shown promise at the *in vitro* or the preclinical level and led to clinical trials, including bone marrow transplantation,^28^ topical injections of allogeneic fibroblasts^29^ or systemic infusions of allogeneic bone marrow derived mesenchymal stromal cells.^30^ However, the risks and/or adverse effects outweighed the benefits. Gene therapy approaches are promising, but at least initially, target limited skin areas and require repeated applications.^31^^,^^32^ In this context, it is important that the treated RDEB skin’s structure be as normal as possible in order to enable a supportive microenvironment for gene-treated or gene-modified keratinocytes and fibroblasts for collagen VII production and the formation of anchoring fibrils attaching the epidermis to the dermis. Inflamed and fibrotic dermis can provide only suboptimal support for cell adhesion and functions and may heighten immunogenicity of therapeutic agents, thus hampering sustainable therapeutic benefits. Here, by mitigating inflammation and fibrosis via losartan, we can expect twice the benefit–first, by improving patients’ quality of life and, second by optimising the skin structure in preparation for a curative treatment.

In conclusion, our trial sets the stage for a randomised, phase III clinical trial to assess the effects of losartan in people with RDEB.

## Contributors

DK: conceptualisation, formal analysis, investigation, methodology, supervision, resources, validation, visualisation, writing—original draft, and writing—review & editing.

FS: investigation, validation, writing—review & editing.

SG: investigation, writing—review & editing.

KR: investigation, writing—review & editing.

ART: investigation, writing—review & editing.

ASB: investigation, writing—review & editing.

HO: investigation, writing—review & editing.

CS: data curation, formal analysis, methodology, validation, visualisation, writing—review & editing.

OG: data curation, methodology, project administration, validation, writing—review & editing.

DM: validation, writing—review & editing, served as member of the Data Safety Review Board.

BS: formal analysis, investigation, methodology, validation, writing—review & editing.

TZ: validation, writing—review & editing.

AN: Formal analysis, Investigation, Methodology, Validation, visualisation, writing—review & editing.

LBT: conceptualisation, funding acquisition, resources, writing—review & editing.

DK, CS, OG, AN and LBT had access to and verified the underlying study data. DK, AN and LBT had the final responsibility to submit for publication.

## Data sharing statement

The full trial protocol can be obtained upon request to the corresponding author. Anonymised data will be made available for reasonable requests to the corresponding author with scientific rationale and sound methodology.

## Declaration of interests

DK, TZ and LBT are founders of Crowd Pharma Losartan GmbH & Co. KG, a company established in 2021 to pursue development of losartan for epidermolysis bullosa. AN has received payments for invited talks by Amryt Pharma. AN, BS, DK and LBT have filed a patent for the use of losartan for the treatment of fibrotic diseases, in particular epidermolysis bullosa (EP 4 081 300 B1). All other authors declare no competing interests.
