# Supplementary material for: Safety and tolerability of losartan to treat recessive dystrophic epidermolysis bullosa in children (REFLECT): an open-label, single-arm, phase 1/2 trial
Source: eClinicalMedicine. 2024 Oct 30;77:102900. doi: 10.1016/j.eclinm.2024.102900 (PMC11558043; doi:10.1016/j.eclinm.2024.102900)
Supplement: Supplemental Figs. S1 and S2 and Tables S1 and S2 [file mmc1.pdf]

Supplemental data

Supplemental Table 1: Routine laboratory data – Ratio to Baseline

Parameter	Visit	Measurement	N	Mean	STD	Median	95%-CI lower limit	95%-CI upper limit	p-value
Hemoglobin	Baseline	Pre	28	11.01	1.15	11.15	-	-	-
	Month 9	Post	28	10.63	1.38	10.45	-	-	-
		Ratio	28	0.96	-	0.96	0.94	0.99	0.0083
	Baseline	Pre	28	110.85	71.10	87.00	-	-	-
NT-proBNP	Month 9	Post	28	123.40	79.41	105.00	-	-	-
		Ratio	28	1.08	-	1.00	0.86	1.37	0.4890
	Baseline	Pre	28	28.64	60.37	5.55	-	-	-
CRP	Month 9	Post	28	26.28	55.67	7.20	-	-	-
		Ratio	28	1.15	-	1.00	0.82	1.62	0.3935
	Baseline	Pre	28	30.50	54.63	16.50	-	-	-
Ferritin	Month 9	Post	28	76.00	220.51	20.50	-	-	-
		Ratio	28	1.46	-	1.26	1.15	1.83	0.0026

Supplemental Table 2: Fibrotic and Inflammatory Markers in Serum – Ratio to Baseline

Parameter	Visit	Measure-ment	N	Mean	STD	Median	95%-CI lower limit	95%-CI upper limit	p-value
IL6	Baseline	Pre	26	39.89	90.39	16.10	-	-	-
	Month 9	Post	26	33.20	65.86	13.25	-	-	-
		Ratio	26	0.96	-	1.00	0.70	1.32	0.8174
	Baseline	Pre	28	14.83	5.77	12.50	-	-	-
TNF	Month 9	Post	28	13.54	6.09	12.00	-	-	-
		Ratio	28	0.89	-	0.90	0.80	0.99	0.0395
	Baseline	Pre	28	117.18	261.00	11.50	-	-	-
SAA	Month 9	Post	28	89.84	216.44	23.00	-	-	-
		Ratio	28	0.90	-	0.87	0.44	1.87	0.7778
	Baseline	Pre	26	104083	39536	95368	-	-	-
TGFβ	Month 9	Post	26	103208	31715	96506	-	-	-
		Ratio	26	1.02	-	0.94	0.90	1.15	0.7950
PINP	Baseline	Pre	26	97.07	88.96	64.19	-	-	-
	Month 9	Post	26	105.37	79.21	73.00	-	-	-
		Ratio	26	1.16	-	1.26	0.92	1.47	0.1929
PIIINP	Baseline	Pre	27	315.83	205.07	265.67	-	-	-
	Month 9	Post	27	309.19	201.71	298.50	-	-	-
		Ratio	27	0.97	-	1.02	0.84	1.12	0.6463

Figure legend: IL6, interleukin 6; TNF, Tumor necrosis factor; SAA, serum amyloid; TGFβ, Transforming growth factor beta; PINP, procollagen I amino-terminal peptide; PIIINP, procollagen III amino-terminal peptide

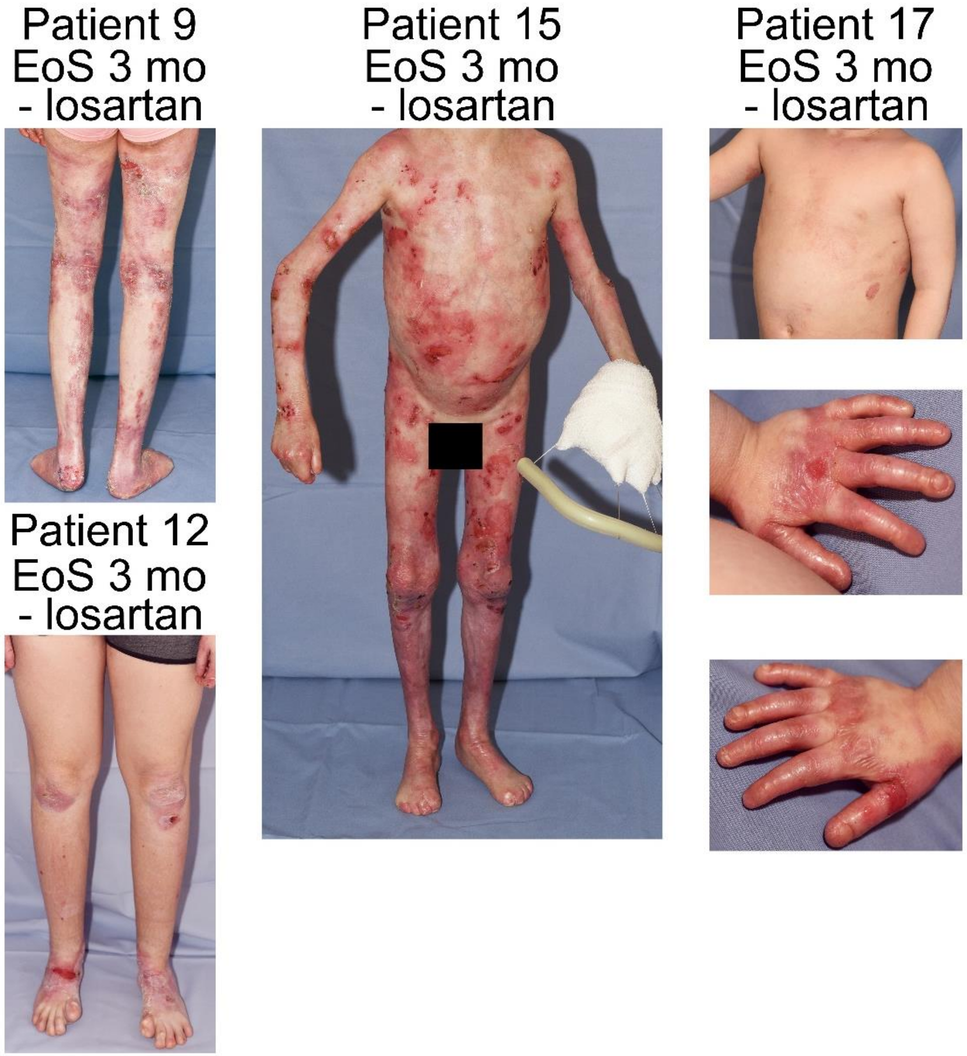

Supplemental Figure 1. **Examples of the patients shown in Figure 3 at the end of study visit (after 3 months of losartan withdrawal).** In general, gradual worsening of the skin condition with elevated wounding is present after discontinuation of losartan for 3 months.

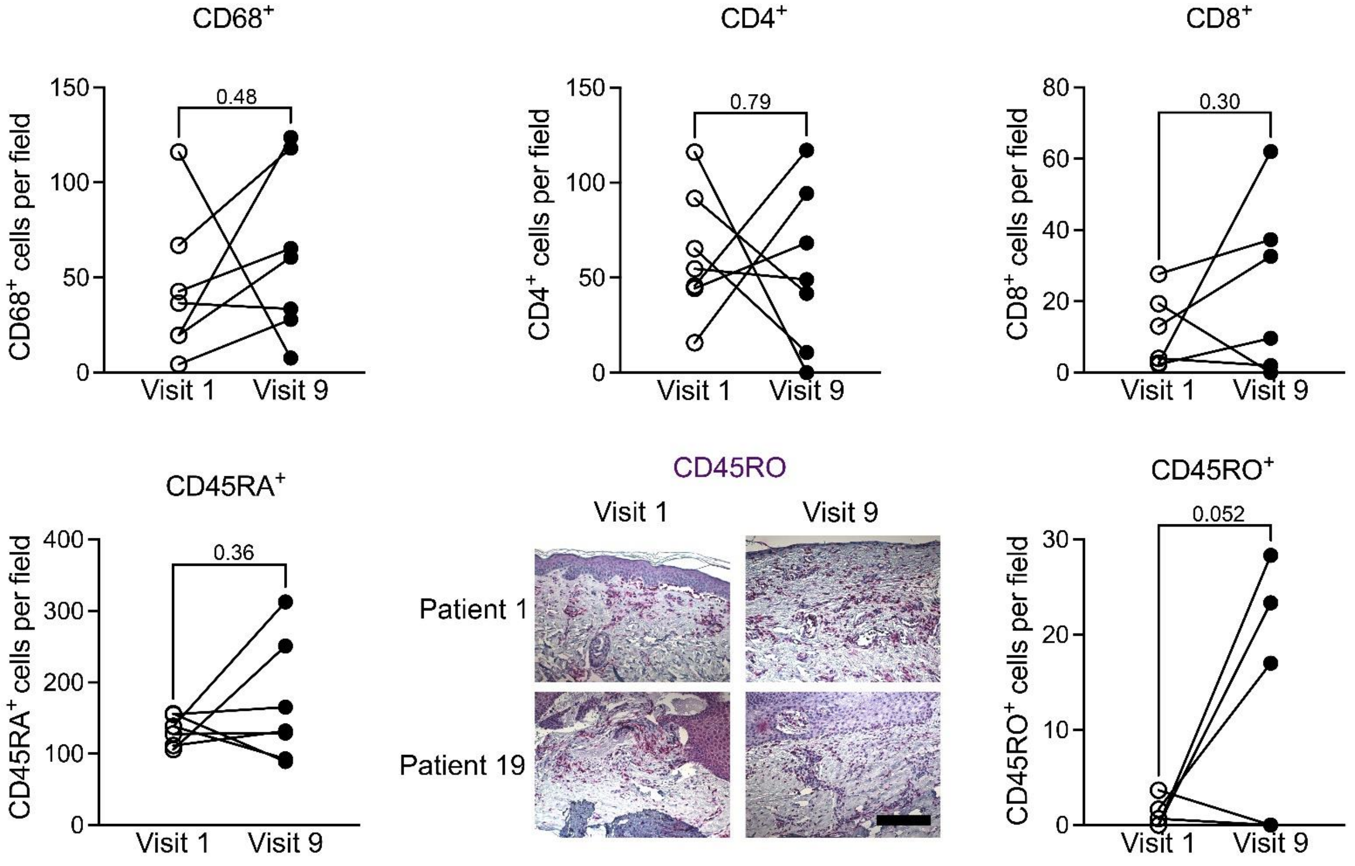

Supplemental Figure 2. **Analyses of skin inflammation markers**

Quantification after stainings against the respective cell surface markers. P values were obtained with the use of Student’s t test. Examples of representation CD45RO stainings are shown at visit 1 and 9 (scale bar, 200 µm).

Clinical Trial Protocol

A dual-center prospective phase I/II trial to establish safety, tolerability and to obtain first data on efficacy of losartan in children with recessive dystrophic epidermolysis bullosa (RDEB)

REFLECT

(symptom-**RE**lie**F** with **L**osartan – **E**pidermolysis bullosa **C**linical **T**rial)

**EudraCT No.**	2015-003670-32
**DRKS -No.**	DRKS00009269
**Internal Protocol ID No.**	P000980
**Protocol Version**	Amendment 1 / 18th January 2017
**Revision chronology**	Clinical Trial Protocol (CTP) Final V 2 / 20th July 2016
**Development Phase**	Phase I/II
**Sponsor**	**Medical Center - University of Freiburg**<br>represented by the Chief Medical Officer (CMO)<br>(Leitender Ärztlicher Direktor)<br>Hugstetter Str. 49<br>D-79106 Freiburg
**Coordinating Investigator**<br>*(LKP in accordance with German Drug Law)*	Dr. Dimitra Kiritsi<br>Department of Dermatology<br>Medical Center - University of Freiburg
**Trial Coordination**	Clinical Trials Unit<br>Medical Center - University of Freiburg

This Clinical Trial Protocol contains confidential information. Circulation of this material to individuals who are not involved in the carrying out of the study or any kind of publication requires the approval of the sponsor. These limitations similarly relate to all confidential information and data which will be obtained in the future.

Approval of the Clinical Trial Protocol

A dual-center prospective phase I/II trial to establish safety, tolerability and to obtain first data on efficacy of losartan in children with recessive dystrophic epidermolysis bullosa (RDEB)

EudraCT No.: 2015-003670-32

Protocol Version No: CTP Amendment 1 / 18th January 2017

Coordinating Investigator

"Leiter Klinische Prüfung/ LKP"
(in accordance with German Drug Law)
Dr. Dimitra Kiritsi — 02.02.2017 — Date — Signature

Medical Trial Coordinator

Dr. Franziska Schauer — 02.02.17 — Date — Signature

Biostatistician

Dr. Claudia Schmoor — 03.02.17 — Date — Signature

Sponsor (representative of the sponsor)

Dr. Dimitra Kiritsi — 02.02.2017 — Date — Signature

Investigator Statement

**Protocol Short Title:**	REFLECT
**EudraCT No.:**	2015-003670-32
**Protocol Version No:**	CTP Amendment 1 / 18th January 2017
**Trial Center:**	<Center No. and Name of Trial Center>
**Investigator:**	<Name of Investigator>

I confirm that I have read the Clinical Trial Protocol and hereby commit myself to adhere to all actions and terms as specified in the relevant sections of the clinical, ethical and general paragraphs.

I confirm that I and my colleagues will abide by the local legislation (in Germany, the German Pharmaceutical Law with the appropriate amendments). I further confirm that the Clinical Trial will be carried out in compliance with the Declaration of Helsinki and ICH-GCP guidelines.

I acknowledge that all confidential information in this document, apart from the evaluation of the Clinical Trial will not be used or circulated without the prior written consent of the Sponsor.

Under my supervision I put copies of this Clinical Trial Protocol and possible updates as well as access to all information regarding the carrying out of this Clinical Trial at the disposal of my colleagues; in particular I will promptly forward all information from the Sponsor in relation to Pharmaceutical Safety (SUSAR, SmPC and IB updates, if applicable) to my colleagues.

I confirm that I and my colleagues were informed by a responsible scientist about the results and expected risks of the pharmacological and toxicological examination associated with the clinical trial.

I will discuss this Clinical Trial Protocol in detail with my colleagues and ensure that they are comprehensively informed about the trial compound/preparation and the execution of the trial.

Furthermore I commit myself not to commence patient enrolment before the approval of the authorities and acceptance by the relevant/responsible Ethics Committee.

Date	Name of Investigator	Signature of Investigator

Table of Contents

Approval of the Clinical Trial Protocol **2**
Investigator Statement **3**
Table of Contents **4**
List of figures **9**
List of Tables **9**
List of Abbreviations **10**
Synopsis **12**
Responsibilities **19**
1 Background and rationale **21**
1.1 Summary 21
1.2 Scientific background 21
1.3 Overview of investigational medicinal product (IMP), losartan 23
1.4 Trial purpose and rationale 23
1.5 Rationale for dose selection 26
1.6 Available treatments for RDEB 26
1.7 Risk-Benefit assessment 27
1.8 Pharmacoeconomics 28
2 Objectives and endpoints **28**
2.1 Primary objective and endpoint 30
3 Clinical trial plan **30**
3.1 Trial design 30
3.2 Treatment arms 30
3.3 Treatment duration 30
3.4 Trial timetable 31
3.5 Participating sites 31
3.6 Number of patients 31
3.7 Recruitment strategy and planned rate 31
4 Trial population and selection criteria **32**
4.1 Target population / main diagnosis 32
4.1.1 Target population 32
4.1.2 Gender distribution 32
4.2 Inclusion criteria 32
4.3 Exclusion criteria 32
5 Enrolment and patient registration **33**
5.1 Patient eligibility 33
5.2 Patient registration 34
6 Treatment plan and procedure **34**
6.1 Dosing regimen and investigational medicinal product administration 35
6.2 Dose modification and dose delay 36
6.3 Permitted concomitant treatment/medication 37

6.3.1 Permitted rescue medication ... 37
6.4 Prohibited concomitant therapy ... 37
6.5 Hormonal contraception allowed ... 37
6.6 Unblinding of treatment assignment ... 37
7 Visit schedule and assessments ... 38
7.1 Flow and visit schedule ... 38
7.2 Visit and assessment windows ... 38
7.3 Screening and registration ... 38
7.3.1 Pre-screening (optional) ... 38
7.3.2 Screening ... 39
7.3.3 Data to be collected on screening failures ... 39
7.3.4 Assessments at screening (Day -14 until day -1) ... 39
7.3.5 Registration (Clinic-Day 1) ... 40
7.4 Treatment phase ... 40
7.4.1 Visit 1 (Clinic- Day 1) ... 40
7.4.2 Visit 2 (Phone- Week 4) ... 40
7.4.3 Visit 3 (Clinic- Week 8) ... 41
7.4.4 Visit 4 (Phone- Week 12) ... 41
7.4.5 Visit 5 (Clinic – Week 16) ... 41
7.4.6 Visit 6 (Home- Week 24) ... 42
7.4.7 Visit 7 (Phone- Week 28) ... 42
7.4.8 Visit 8 (Phone- Week 34) ... 42
7.4.9 Visit 9 (Clinic- Week 40) ... 42
7.4.10 End of treatment (EOT) visit (Phone- Week 44) ... 43
7.5 End of study (EOS) visit (Clinic- Week 57) ... 43
7.6 Description of examinations during the trial ... 44
7.6.1 Patient demographics ... 44
7.6.2 Medical and RDEB disease history ... 44
7.6.3 Pregnancy test ... 44
7.6.4 Physical examination ... 44
7.6.5 Height and weight ... 44
7.6.6 Pulse rate and blood pressure ... 45
7.6.7 Documentation of fibrosis and disease burden (scores) ... 45
7.6.8 Morphometric scoring instrument of pseudosyndactyly progression ... 46
7.6.9 Laboratory tests (local assessment) ... 46
7.6.10 Fibrotic and inflammatory markers in serum (central assessment) ... 46
7.6.11 Electrocardiogram (ECG) and echocardiography ... 47
7.6.12 Skin biopsy (central) ... 47
7.6.13 Itch, pain, QOL and exacerbating ... 47

7.7 Treatment after end of the trial ... 48
8 Discontinuation criteria ... 48
8.1 Premature termination of the entire trial ... 48
8.2 Premature termination of the trial at one of the trial centres ... 49
8.3 Discontinuation criteria for individual trial patients ... 50
8.3.1 Premature discontinuation of trial treatment ... 50
8.3.2 Premature termination of trial participation ... 51
9 Investigational medicinal product (IMP) ... 51
9.1 Pharmacokinetics ... 51
9.2 Side effects of IMP ... 52
9.3 IMP specifications ... 52
9.4 Packaging, labelling, supply and ordering ... 53
9.5 Receipt and storage ... 53
9.6 Dispensing ... 53
9.7 Return and destruction ... 53
9.8 Drug compliance and accountability ... 53
9.9 Treatment adherence ... 54
10 Safety monitoring and reporting ... 55
10.1 Adverse events (AEs) ... 55
10.1.1 Definition of AEs ... 55
10.1.2 Documentation of AEs ... 56
10.2 Serious Adverse Events (SAEs) ... 56
10.2.1 Definition of SAEs ... 56
10.2.2 Documentation of SAEs ... 57
10.2.3 Investigator reporting requirements ... 57
10.2.3.1 SAE reporting policy 57
10.2.3.2 Reporting of patient death 57
10.2.3.3 Reporting of premature treatment/study discontinuation 58
10.2.4 Sponsor reporting requirements ... 58
10.2.4.1 Definition of SUSARs 58
10.2.4.2 SUSAR/ circumstance requiring a review of the benefit/risk evaluation 58
10.2.4.3 Development Safety Update Report (DSUR) 58
10.2.5 Pregnancies ... 59
11 Data handling and data management ... 59
11.1 Data confidentiality ... 59
11.2 Documentation of trial data ... 59
11.2.1 Documentation in medical records ... 59
11.2.2 Documentation in CRF ... 60
11.3 Data management ... 60
11.4 Data coding ... 61
12 Quality assurance ... 61

12.1 Monitoring procedure 61
12.2 Source data verification (SDV) 62
12.3 Auditing procedures and inspections 62
13 Biostatistical planning and analysis 62
13.1 Trial design........ 63
13.2 Objectives and endpoints 63
13.3 Sample size calculation 63
13.4 Definition of populations included in the analyses........ 63
13.5 Methods of analysis........ 63
13.5.1 Patient demographics/other baseline characteristics 63
13.5.2 Treatments (trial treatment and compliance, concomitant medication) 64
13.5.2.1 Trial medication 64
13.5.2.2 Concomitant medication 64
13.5.3 Primary endpoint (safety) 64
13.5.4 Secondary endpoints for efficacy........ 64
13.5.5 Secondary endpoints for safety 65
13.5.5.1 Adverse events 65
13.5.5.2 Laboratory data 65
13.6 Interim analysis 65
14 Data safety monitoring committee (DSMC) 65
15 Ethical and legal principles 66
15.1 Regulatory and ethical compliance........ 66
15.2 Responsibilities of the investigator and IEC........ 66
15.3 Informed consent procedure........ 66
15.4 Patient insurance........ 68
15.5 Confidentiality of trial documents and patient records........ 68
15.6 Financial disclosure........ 68
16 Trial documents and archiving........ 68
16.1 Trial documents/investigator site file........ 68
16.2 Archiving 69
17 Protocol adherence and amendments........ 69
17.1 Protocol adherence 69
17.2 Amendments to the protocol........ 69
17.3 Protocol deviations........ 70
18 Administrative Agreements........ 70
18.1 Financing of the trial and role of the funder........ 70
18.1.1 Trial agreement/investigator compensation 70
18.1.2 Reimbursement of trial patients........ 70
18.2 Trial reports........ 70
18.3 Clinical trials registry 71
18.4 Publication of trial protocol and results 71

19 References..72
Appendices..74
Appendix 1 Relevant Guidelines and Laws..74
Appendix 2 The Physician Global Assessment (PGA)..75
Appendix 3 The Birmingham Epidermolysis Bullosa Severity Score (BEBS)......76
Appendix 4 The Epidermolysis Bullosa Activity and Scarring Index (EBDASI)....78
Appendix 5 Hand Function Assessment Score by Colville & Terill *...................82
Appendix 6 The Mayo Dysphagia Questionnaires-day 30 (MDQ-30)..................83
Appendix 7 Itch assessment scale for the pediatric burn patients.......................90
Appendix 8 Wong Baker Faces Scale for pain..91
Appendix 9 QoLEB...92
Appendix 10 Children’s Dermatology Life Quality Index (CDLQI).........................93
Appendix 11 Exacerbating factors...95
Appendix 12 Morphometric pseudosyndactyly scoring..96
Appendix 13 Fibrotic and inflammatory markers in skin biopsy specimens...........97
Appendix 14 The fibrotic and inflammatory markers in serum..............................98

List of figures

Figure 1 Losartan treatment delays pseudosyndactyly formation in RDEB mice 26
Figure 2 Dosing visit flowchart 35

List of Tables

Table 1 Objectives and related endpoints 28
Table 2 Treatment schedule 36

List of Abbreviations

AE	Adverse Event
AMG	German Drug Law (Arzneimittelgesetz)
AT1R	Angiotensin II Type 1 Receptor
BEBS	Birmingham Epidermolysis Bullosa Severity Score
Beta-hCG	Beta-human Chorionic Gonadotropin
BID	Twice a day
BP	Blood Pressure
CA	Competent Authority
CDLQI	Chirdren's Dermatology Life Quality Index
Clinic	Refers to the day clinic setting
CONSORT	Consolidated Standards of Reporting Trials
CRA	Clinical Research Associate (Monitor)
CRF	Case Report Form
CRP	C-reactive protein
CT	Computerised Tomography
CTCAE	Common Terminology Criteria for Adverse Events
CTU	Clinical Trials Unit
DAMAST	SAS®-based data management system
DEB	Dystrophic epidermolysis bullosa
DEBRA	Dystrophic Epidermolysis Bullosa Research Association
DMBA	7,12-Dimethylbenzaanthracene
DRKS	Deutsches Register Klinischer Studien (German Clinical Trials Register)
DSMC	Data Safety Monitoring Committee
DSUR	Development Safety Update Report
EB	Epidermolysis Bullosa
EBDASI	Epidermolysis Bullosa Disease Activity and Scarring Index
ECG	Electrocardiogram
ELISA	Enzyme Linked Immunosorbent Assay
EMA	European Medicines Agency
EOT	End of Treatment
EOS	End of Study
EudraCT	European Union Drug Regulating Authorities Clinical Trials
FDA	Food and Drug Administration
FPFV	First Patient First Visit
FU	Follow Up
GCP-V	German Decree of 09-Aug-2004 on the Use of Good Clinical Practices
GMP	Good Manufactoring Practice
GP	General Practitioner
Hb	Hemoglobin
HBV	Hepatitis-B-Virus
HCV	Hepatitis-C-Virus
HIV	Human Immunodeficiency Virus
i.v.	Intravenous(ly)
IB	Investigator's Brochure
ICF	Informed Consent Form
ICH-GCP	ICH Topic E6: Guideline for Good Clinical Practice (GCP)
IEC	Independent Ethics Committee
IL6	Interleukin 6
IMP	Investigational Medicinal Product /study medication
LPLV	Last Patient Last Visit

MDQ-30	Mayo Dysphagia Questionnaire-day 30
MSC	Mesenchymal Stromal Cells
NCT No	National Clinical Trial (NCT) number, another term for the ClinicalTrials.gov registry
NT-proBNP	N-terminal propeptid Brain (B-type) Natriuretic Peptide
NSAIDS	Non-steroidal anti-inflammatory drugs
pFAK	Phosphorylated Focal Adhesion Kinase
PGA	Physician Global Assessment
PHI	Protected Health Information
PI	Principal Investigator
PIIINP	Procollagen III N-terminal propeptide
PINP	Procollagen type I N-terminal propeptide
PP	Per-Protocol
PR	Pulse Rate
QOL	Quality of Life Questionnaire
RDEB	Recessive Dystrophic Epidermolysis Bullosa
RSI	Reference Safety Information (current SmPC or/and current IB)
SAE	Serious Adverse Event
SAP	Statistical Analysis Plan
SAR	Serious Adverse Reaction
SAS	Statistical Analysis System
SCC	Squamous Cell Carincoma
SDV	Source Data Verification
SMAD	Sma and Mad related Proteins
SmPC	Summary of Product Characteristics (Fachinformation)
SOP	Standard Operating Procedure
SUSAR	Suspected Unexpected Serious Adverse Reaction
TPA	Tissue Polypeptid Antigen
TGFβ	Transforming growth factor beta
TNFα	Tumor necrosis factor alpha
WHO	World Health Organization

Synopsis

**TITLE OF TRIAL**	A dual-center prospective phase I/II trial to establish safety, tolerability and to obtain first data on efficacy of losartan in children with recessive dystrophic epidermolysis bullosa (RDEB)
**SHORT TITLE**	**REFLECT**
**PROTOCOL NO**	Internal Protocol Number: P000980
**EUDRACT NO**	2015-003670-32
**MAIN DIAGNOSIS**	Recessive dystrophic epidermolysis bullosa (RDEB)
**PHASE**	Phase I/II
**OBJECTIVES**	To establish tolerability and safety of losartan in children with moderate to severe RDEB.<br>The secondary objective is to obtain first information on the efficacy of losartan in improving the disease manifestations and quality of life, and reducing inflammation and fibrosis in moderate to severe RDEB over a period of 9 months
**INTERVENTION**	Experimental intervention: Losartan dose escalation from 0.4 to 1.4 mg/kg within 16 weeks, the dose of 1.4 mg/kg within 24 weeks and dose tapering within 4 weeks<br>Control intervention: not applicable<br>Duration of intervention per patient: 10 months<br>Follow-up per patient: 3 months

**INCLUSION CRITERIA**	1. Written informed consent of parents or legal guardians obtained according to international guidelines and local laws;<br>2. Patient's assent (if applicable according to patient's age and understanding);<br>3. Male or female patients from 2 to 16 years (age of > 25 months);<br>4. Molecularly confirmed diagnosis of <u>moderate to severe RDEB</u>. If the patient is completely collagen VII-deficient, as shown by negative collagen VII immunofluorescence staining of a skin biopsy, no genetic confirmation of the diagnosis will be required for inclusion in the study. In case of residual collagen VII expression, the *COL7A1* gene will be analyzed for mutations, to confirm the diagnosis of RDEB;<br>5. Ability of the patient (if applicable according to patient's age and understanding), parents or legal guardians to understand the nature of the trial and trial-related procedures and to comply with them;<br>6. Able to travel to trial site for all clinic visits;
**EXCLUSION CRITERIA**	1. Simultaneous or previous participation in any interventional trial within the past 3 months before entering this trial; participation in simultaneous registry and diagnostic trials during the trials is allowed;<br>2. Anemia with hemoglobin < 8 g/dl;<br>3. Hypotension (defined as age-related systolic blood pressure under the 5th percentile);<br>4. Cardiologic contraindications, such as severe heart failure with ejection fraction < 35%;<br>5. Patient requires any medications that are likely to cause interactions with losartan, e.g. rifampicin, ACE-inhibitors;<br>6. Renal artery stenosis or renal insufficiency with creatinine clearance < 30 ml/min;<br>7. Severe liver failure;<br>8. Severe, untreated electrolyte disturbances;<br>9. History of cancer or chronic viral infections (HBV, HCV, HIV);<br>10. Hypersensitivity to losartan or any of the excipients;<br>11. Known or persistent abuse of medication, drugs or alcohol;<br>12. Persons who are in a relationship of dependence/employment with the sponsor or the investigator.<br>13. Current pregnancy or nursing period;<br>14. For female patients with menarche: unwillingness to use adequate contraception or to stay sexually abstinent during the course of the trial;

**PRIMARY ENDPOINT**	Primary endpoint is defined as occurrence of a serious safety concern, specified as one of the following side effects of losartan:<br>1) clinically relevant severe hypotension i.e. the patient experiences continuous dizziness, headaches and signs of peripheral shut down owing to the low blood pressure, leading to interruption of study medication<br>2) immediate hypersensitivity reactions to the drug<br>3) clinical relevant sever hypo- and hyperkalemia
**SECONDARY ENDPOINTS**	(Serious) adverse events, evaluated by monitoring heart rate and function and blood pressure, using echocardiography, home blood pressure monitoring devices, and blood tests throughout the study.<br>Efficacy will be assessed using validated scoring systems for the clinical manifestations of RDEB:<br>• Physician Global Assessment (PGA)<br>• Birmingham Epidermolysis Bullosa Severity Score (BEBS)<br>• Epidermolysis Bullosa Disease Activity and Scarring Index (EBDASI)<br>• Score of Colville and Terrill<br>• Our own morphometric scoring instrument of pseudosyndactyly progression<br>• Mayo Dysphagia Questionnaire-day 30 (MDQ-30)<br>• Itch Assessment Scale for the Pediatric Burn Patients<br>• Wong-Baker FACES Scale for Pain<br>• Quality Of Life in EB (QOLEB) questionnaire<br>• Children's Dermatology Life Quality Index (CDLQI)<br>• Assessment of fibrotic and inflammatory markers in skin and blood
**TRIAL DESIGN**	Open-label, non-randomized, single arm, bi-center study

<table>
<tr><td rowspan="2">STATISTICAL ANALYSIS</td><td colspan="2"><u>Sample size calculation:</u>
Sample size calculation is based on the primary endpoint occurrence of severe complication leading to a serious safety concern. A total of 30 trial participants is necessary to achieve sufficient power for an assessment of the primary endpoint. If none of the 30 participants develops a severe complication leading to a serious safety concern, it can be concluded that, with a probability of 95%, the severe complication rate is below 10%.
<u>Population included in the analyses:</u>
All analyses (safety and efficacy) will be performed in the safety population. Patients are included in the safety population, if they received at least one dose of trial medication with losartan.
<u>Primary endpoint analysis (safety):</u>
All severe complication leading to a serious safety concern will be listed by patient with the given dose of trial medication. The incidence of these severe complications will be calculated as the number of patients who experienced at least one severe complication in percentage of the total number of patients in the safety population. Additionally, the 90%- and the 95%-confidence intervals of the incidence will be calculated. If in none of 30 patients a severe complication leading to a serious safety concern occurs, it can be concluded that, with a probability of 95%, the severe complication rate is below 10%.
<u>Secondary endpoints for efficacy:</u>
All patients will receive losartan; First data on efficacy will be assessed in comparison to their own baseline values before start of therapy. Baseline can be regarded as adequate control at this stage, since a spontaneous improvement of inflammation, fibrosis and mitten deformities cannot be expected and therefore an improvement can be regarded as success of the treatment. The efficacy endpoints will be analyzed by comparing post-treatment measurements after 8 weeks and after 9 months with pre-treatment measurements and by calculating 95% confidence intervals for the differences.
<u>Secondary endpoints for safety (descriptive analyses):</u>
All adverse events (AEs) will be listed by center and patient and displayed in summary tables according to MedDRA. Laboratory data will be presented in shift tables for all parameters.
<u>Interim analysis:</u>
Not applicable</td></tr>
<tr></tr>
<tr><td rowspan="2">SAMPLE SIZE</td><td>To be assessed for eligibility:</td><td>n = 50</td></tr>
<tr><td>To be allocated to trial:</td><td>n = 30</td></tr>
</table>

	To be analyzed:	n = 30
**TRIAL DURATION**	Recruitment period (months):	18 months
	First patient in to last patient out (months):	32 months
	Duration of the entire trial (months):	44 months
	Treatment duration per patient (months):	10 months
**TIMETABLE**	Enrolment of first patient (FPFV) (registration)	1st quarter 2017
	Enrolment of last patient (registration)	3rd quarter 2018
	End of trial for last patient (LPLV)	4th quarter 2019
	Final statistical analysis	2nd quarter 2020
	Planned interim analysis	Not applicable
	DSMC phone meeting	After visit 3 of 15th patient
**PARTICIPATING CENTERS**	Two sites: Freiburg (Germany) and Salzburg (Austria)	
**FUNDER**	DEBRA International / Registered Charity No: ZVR 932762489.	

Visit schedule and assessments – Flowchart

		Pre-screening	SCREENING	TREATMENT									Tape ring	End of Study (EOS)
	Duration	*14 days*	*14 days*	*9 months*									*4 weeks*	*3 months*
	Visits	*(optional)*	*Screening*[1]	*Visit 1*[1] *(clinic)*	*Visit 2 (phone)*	*Visit 3 (clinic)*	*Visit 4 (phone)*	*Visit 5 (clinic)*	*Visit 6 (home)*	*Visit 7 (phone)*	*Visit 8 (phone)*	*Visit 9 (clinic)*	*EOT*[3] *(phone)*	*(clinic)*
**Assessments**	Time	*Day-28 until day -15*	*Day -14 until day -1*	***Day 1*** *(Week 1)*	*Day 28* ***(Week 4)*** *(± 5 days)*	*Day 56* ***(Week 8)*** *(± 7 days)*	*Day 84* ***(Week 12)*** *(± 7 days)*	*Day 112* ***(Week 16)*** *(± 7 days)*	*Day 168* ***(Week 24)*** *(± 7 days)*	*Day 196* ***(Week 28)*** *(± 7 days)*	*Day 238* ***(week 34)*** *(± 7 days)*	*Day 280* ***(Week 40)*** *(± 7 days)*	*Day 309* ***(Week 44)*** *(± 7 days)*	*EOT+ 90 days* ***(Week 57)*** *(± 7 days)*
**Informed consent**			X											
**Inclusion / exclusion criteria**			X											
**Demographics, medical history**			X											
**Urine pregnancy test (if applicable)**			(X)	(X)		(X)		(X)				(X)		
**Serology (HBV, HCV, HIV)**		X*	(X)											
**Hemoglobin (Hb)**		X*	(X)	X		X		X				X		X
**Registration (central)**				X										
**Physical examination** [2]			X	X		X		X				X		X
**Body height**			X			X		X				X		X
**Body weight**			X	X**	X	X	X	X	X	X	X	X	X	X
**Heart rate and blood pressure** [3]			X	X	X*	X	X*	X	X*	X	X	X	X*	X
**Documentation of fibrosis and disease burden (scores)**			X	X**		X		X				X		X
**Morphometric pseudosyndactyly scoring (central)**			X			X		X				X		X
**Laboratory tests (local)** [4]			X	X**		X		X				X		X
**Markers of inflammation and fibrosis in serum (central)** [5]			X	X**		X		X				X		X
**Electrocardiogram (ECG)**			X	X**		X		X				X		X
**Echocardiography**			X	X**		X		X				X		X
**Skin biopsy (central)** [6]				X***								X***		
**Itch, pain, QOL scores**			X	X**		X		X				X		X
**Exacerbating factors**			X	X**	X	X	X	X	X	X	X	X	X	X
**IMP administration**					X (daily from day 1 until the day before EOT)									
**IMP dispensation**				X	X	X	X	X	X	X	X	X	X	
**Concomitant medication**			X	Continuously, see section 6.3										X
**Adverse events**				Continuously, see section 10										X

EOT = End of Treatment; FU = Follow Up; IMP = Investigational Medicinal Product; X = once; (X) = if, applicable; local= tests will be performed at study site; central= samples have to be sent to the central assessment (for details see study specific laboratory manual)

* Assessments recommended to be performed, no documentation in the CRF
** Assessment has only to be performed if the time elapsed between screening Visit and Visit 1 is longer than 7 days
*** Optional skin biopsy to obtain first data on inflammational pattern in EB affected skin

(1) If screening and visit 1 are done on the same day, registration and the administration of IMP have to be done subsequently to all screening evaluations.

(2) Physical examination is recommended to be performed according to the flow chart; detailed findings concerning these examinations must only be documented in the CRF at screening. At other visits, in case of clinically relevant abnormal findings, the investigator has to document an AE on the AE-page in the CRF.

(3) Heart rate and blood pressure will be measured daily, either during clinic visits or BID at the patient's home (documented in a patient's diary). Results will be checked by investigator during clinic and phone visits.

(4) Laboratory tests (local assessment) will be performed according to clinical praxis and comprise: blood electrolytes, urea, creatinine, GOT, GPT, LDH, cholinesterase, full cell blood count, NT-proBNP, CRP and ferritin. In the CRF only the following parameters will be documented: hemoglobin, NT-proBNP, CRP and ferritin.

(5) Markers of inflammation and fibrosis in blood (central assessment) comprise: amyloid, TGFβ, IL6, TNFα, PINP, PIIINP (for details see Appendix 14). At the end of the study, the central results will be converged with the clinical database.

(6) The use of topical corticosteroids is not permitted at the biopsy sites (hands or feet) one month before visit 1 and 9, where the skin biopsies will be taken. The results of the staining of skin biopsies will be assessed centrally and will not be documented in the CRF. At the end of the study, they will be converged with the clinical database

Responsibilities

**Sponsor**	Name:	**Medical Center-University of Freiburg**
	Institution:	Department of Dermatology
	Address:	Hauptstrasse 7, 79104 Freiburg
	Telephone:	+49 761 270-67 141
**Coordinating Investigator**	Name:	**Dimitra Kiritsi**
"Leiter der Klinischen Prüfung / LKP" (in accordance with German Drug Law)	Institution:	Department of Dermatology, Medical Center-University of Freiburg
	Address:	Hauptstrasse 7, 79104 Freiburg
	Telephone:	+49 761 270-67 141
	Fax:	+49 761 270-67 141
	E-Mail:	dimitra.kiritsi@uniklinik-freiburg.de
**Medical Trial Coordinator**	Name:	**Franziska Schauer**
	Institution:	Department of Dermatology, Medical Center-University of Freiburg
	Address:	Hauptstrasse 7, 79104 Freiburg
	Telephone:	+49 761 270-69 950
	Fax:	+49 761 270-67 141
	E-Mail:	franziska.schauer@uniklinik-freiburg.de
**Biostatistician**	Name:	**Claudia Schmoor**
	Institution:	Clinical Trials Unit Freiburg
	Address:	Elsaesser Str. 2, 79110 Freiburg, Germany
	Telephone:	+49 761 270-73 710
	Fax:	+49 761 270-73 770
	E-Mail:	claudia.schmoor@uniklinik-freiburg.de
**Registration Office**	Institution:	Clinical Trials Unit Freiburg
	Address:	Elsaesser Str. 2, 79110 Freiburg, Germany
	Fax:	+49 761 270-74 390
**Pharmacovigilance**	Institution:	Clinical Trials Unit Freiburg
**SAE-Management**	Address:	Elsaesser Str. 2, 79110 Freiburg, Germany
	Fax:	+49 761 270- 74 390
	E-Mail:	stuz-pv@uniklinik-freiburg.de
**Project Coordinator**	Name:	**Ralf Tostmann**
	Institution:	Clinical Trials Unit Freiburg
	Address:	Elsaesser Str. 2, 79110 Freiburg, Germany
	Telephone:	+49 761 270- 77 110
	Fax:	+49 761 270- 73 730
	E-Mail:	ralf.tostmann@uniklinik-freiburg.de
**Monitoring**	Institution:	Clinical Trials Unit Freiburg
	Address:	Elsaesser Str. 2, 79110 Freiburg, Germany
	Telephone:	+49 761 270-77 780
	Fax:	+49 761 270-73 730
**Data Management**	Institution:	Clinical Trials Unit Freiburg
	Address:	Elsaesser Str. 2, 79110 Freiburg, Germany
	Telephone:	+49 761 270- 77 970
	Fax:	+49 761 270- 74 390
**Other Medical/ Technical Departments/ Institutes**	**Institution 1:**	Department of Dermatology, Medical Center- University of Freiburg

	Name:	**Alexander Nyström**
	Address:	Hauptstrasse 7, 79104 Freiburg
	Telephone:	+49 761 270-68 950
	Fax:	+49 761 270-67 200
	E-Mail:	alexander.nystroem@uniklinik-freiburg.de
	**Institution 2:**	Department for Congenital Heart Disease<br>University Heart Center Freiburg
	Name:	**Brigitte Stiller**
	Telephone:	+49 761 270-43 230
	Fax:	+49 761 270-44 680
	E-Mail:	brigitte.stiller@universitaets-herzzentrum.de
	**Institution 3:**	Medical Center –University of Freiburg<br>Institute for Clinical Chemistry and Laboratory Medicine
	Name:	**Christine Contini**
	Telephone:	+49 (0)761 270 36170
	Fax:	+49 (0)761 270 73940
	E-Mail:	christine.contini@uniklinik-freiburg.de
**Data Safety Monitoring Committee (DSMC)**	Name:	**Dedee Murrell**
	Institution	Department of Dermatology, St George Hospital, School of Medicine, University of New South Wales
	Profession	Director of Dermatology
	Address	Sydney, New South Wales, Australia
	Telephone:	+61 (2)9113 2543
	Fax:	+61 (2)9113 2906
	E-Mail:	d.murrell@unsw.edu.au
	Name:	**Andreas Gamillscheg**
	Institution:	Division of Pediatric Cardiology, Department of Pediatrics, Medical University Graz
	Profession:	Pediatric cardiologist
	Address:	Auenbruggerplatz 30<br>A-8036 Graz, Austria
	Telephone:	+43 (0) 316 385 82677
	Fax:	+43 (0) 316 385 3675
	E-Mail:	andreas.gamillscheg@medunigraz.at
	Name:	**Anja Sander, M.Sc.**
	Institution:	Institut für Medizinische Biometrie und Informatik, Universität Heidelberg
	Profession:	Statistician
	Address:	Im Neuenheimer Feld 305<br>69120 Heidelberg
	Telephone:	+49 6221 56 5859
	E-Mail:	sander@imbi.uni-heidelberg.de
**Central Pharmacy**	Name:	**Marija Tubic-Grozdanis**
	Institution:	Universitätsmedizin der Johannes Gutenberg-Universität Mainz Apotheke; Abteilung für klinische Studien
	Address:	Langenbeckstr. 1 55131 Mainz
	Telephone:	+49 6131-17-5359
	Fax:	+49 6131-17-3439
	E-Mail:	tubic-grozdanis@unimedizin-mainz.de

1 Background and rationale

1.1 Summary

Recessive dystrophic EB (RDEB) is a severe, chronically disabling genetic skin disorder with urgent need for efficacious treatments. Mechanically induced skin blistering, painful wounds and excessive scarring progressing to fibrosis limit functionality and reduce the quality of life of affected individuals. In spite of numerous research efforts worldwide, a cure for RDEB remains very challenging and cannot be implemented soon. Based on new knowledge on disease mechanisms and highly encouraging preclinical studies in the RDEB mouse model, we propose to test losartan as symptom-relief therapy for moderate to severe RDEB. The rationale is based on inhibition of excessive TGFβ activity in injury-driven inflammation and fibrosis by losartan, an orally administered drug already approved for the treatment of hypertension. Losartan prevents progressive fibrosis also in humans, as shown in children with another genetic disorder, the Marfan syndrome. All these data strongly suggest that losartan has the potential to reduce inflammation, excessive scarring and fibrosis in RDEB. This trial is a prospective, open, single-arm phase I/II clinical study with the primary objective to establish safety and tolerability of losartan in moderate to severe RDEB. A secondary objective is to obtain first information on the efficacy of losartan in improving the disease manifestations and quality of life, and reducing inflammation and fibrosis. The target population comprises patients of 2 - 16 years of age with molecularly confirmed diagnosis of moderate to severe RDEB, who do not yet exhibit severe scarring. It is important to start the treatment as early as possible in order to prevent the progression of scarring to fibrosis, and to maintain functionality. Our main hypothesis is that the young children with RDEB will profit from losartan treatment the most, and that the efficacy data in this population will be most tangible.

1.2 Scientific background

Dystrophic epidermolysis bullosa (DEB) is a monogenic skin fragility disorder caused by mutations in the *COL7A1* gene. This gene encodes collagen VII – a specialized extracellular matrix protein and the major component of anchoring fibrils at the dermal-epidermal junction. Reduced functionality of collagen VII results in diminished epidermal-dermal adhesion and decreased resistance of the skin against frictional forces, visible as skin blistering. DEB is genetically and clinically heterogeneous, both autosomal dominant (DDEB) and recessive (RDEB) subtypes exist. The disease spectrum ranges from localized blisters to severe generalized phenotypes with multi-organ involvement. Severe RDEB is a devastating disorder with skin and mucosal involvement, soft tissue fibrosis (e.g. esophageal stenosis), formation of pseudosyndactylies and mitten deformities and high risk of aggressive squamous cell carcinoma (Intong and Murrell, 2012). In spite of worldwide efforts to develop causative therapies for RDEB, including cell-, gene- and protein-based therapies, large-scale clinical implementation of such therapies is still in the distant future as major hurdles involving efficiency and safety have to be overcome. Consequently, the unmet medical need remains high.

In a wider perspective, RDEB is a “collagen disorder” and belongs to the group of hereditary connective tissue diseases (Klett and Diegelmann, 1997). These are commonly caused by mutations in genes encoding structural extracellular matrix proteins. The mutations frequently disrupt the tissue microarchitecture and alter its biophysical performance. Conceptually

important are recent research findings suggesting that the pathology of many connective tissue diseases is based on the loss of the instructive and regulatory functions of the matrix, instead of its structural properties. It has become evident that secondary disease mechanisms govern phenotypic manifestations in connective tissue disorders via e.g. unfolded protein response, dysregulated autophagy, or alteration of TGFβ bioavailability (Boot-Handford and Briggs, 2010; Neill et al., 2014; Wheeler et al., 2014). Intriguingly, targeting these mechanisms may offer an opportunity to treat the disorders without correcting the underlying genetic defect.

A number of observations indicate that mechanisms secondary to the causal *COL7A1* mutations control disease manifestations and evolution of RDEB, too. First, although there is a tendency that individuals expressing partially functional collagen VII have milder phenotypes than individuals completely devoid of collagen VII, this does not apply to all patients (Varki et al., 2007). Second, the disorder and in particular scarring and fibrosis, is progressive, indicating that additional mechanisms guide disease evolution (Fine and Mellerio, 2009). Using patient tissue samples and genetic RDEB mouse models we and others have observed upregulation of TGFβ activity in injured collagen VII-deficient skin. TGFβ activity was also identified as a natural disease modifier in monozygotic twins differently affected by RDEB: milder clinical manifestations were linked to endogenous upregulation of decorin, a TGFβ- sequestering and neutralizing small proteoglycan (Odorisio et al., 2014). The reason for excessive TGFβ activity in injured RDEB skin is not completely understood, but it involves a number of factors, such as its elevated expression, and increased release from dermal microstructures or from infiltrating inflammatory cells.

TGFβ signaling is complex. TGFβ is secreted from cells as a latent complex, which is sequestered in the extracellular matrix. To be active, TGFβ needs to be released from the latent complexes, e.g. by tractional forces from integrins and thrombospondin-1 or through proteolysis (Dietz, 2015). Active TGFβ signals through TGFβ receptor I and II. Upon binding of TGFβ, the receptors become phosphorylated and transmit signals through both the canonical TGFβ signaling pathway, involving SMAD proteins, and the non-canonical pathway, involving PI3K, JNK and ERK (Zhang, 2009). Physiologically, the TGFβ signaling pathway is activated during development, and in adulthood in processes like tissue regeneration and regulation of immune responses. Pathological, dysregulated activation occurs in cancer and fibroproliferative disorders, creating strong incentives for pharmacological modulation of TGFβ signaling. However, the tissue- and context-specificity of TGFβ signaling has challenged the development of clinically useful TGFβ inhibitors. In principle, interfering with expression/activation, or sequestering and neutralization of ligands, or inhibition of receptors or downstream signaling proteins can block the activity. However, a problem has been posed by the fact that TGFβ signaling has a remarkable ability to be restored through compensatory upregulation of pathway components (Dietz, 2015). This suggests that the more levels of the TGFβ pathway that are targeted by one compound, the better the chances of effective interference. One compound with multi-level interference is losartan, an antagonist of the renin-angiotensin system, which is connected to TGFβ signaling.

We assessed losartan as treatment for RDEB in a preclinical setting in a mouse model and showed that it effectively lowered elevated TGFβ levels in serum and injured skin, attenuated fibrosis in injured forepaws, reduced joint contraction and development of mitten deformities. Losartan also effectively and rapidly resolved tissue inflammation.

Based on the above considerations, losartan seems an ideal drug for safe and efficient long-term targeting of the TGFβ signaling pathway, since it antagonizes TGFβ activity on multiple levels by reducing expression of TGFβ ligands, activators and receptors in parallel. Although

not a cure, such a treatment should ameliorate clinical features, symptoms and improves functionality and quality of life. Losartan is available in all European pharmaceutical markets, is inexpensive and has been safely used in adults and children for more than two decades. Moreover, repurposing approved drugs to treat a disease offers a way to faster and safer clinical implementation than developing new drugs.

1.3 Overview of investigational medicinal product (IMP), losartan

The drug tested in this trial is losartan, an antagonist of the renin-angiotensin system, which is connected to TGFβ signaling (Zhang, 2009). Losartan is an AT1R antagonist that is FDA/EMA approved for treatment of hypertension. Apart from lowering blood pressure, blockage of AT1R signaling also silences TGFβ activity (Habashi et al., 2011).

In preclinical studies losartan reduced fibrosis in a spectrum of disorders linked to dysregulated TGFβ bioavailability like Marfan syndrome, muscular dystrophies, myocardial, pulmonary and renal fibrosis. In clinical trials losartan has been effective in slowing down fibrosis and reducing circulating TGFβ levels in several fibrotic conditions, which are directly dependent on TGFβ activity (Ramirez and Rifkin, 2012). In contrast, the effect in Marfan syndrome has been harder to assess. Although losartan slows aortic root dilatation in Marfan syndrome, the effect may not be superior to other antihypertensive drugs, suggesting that the primary effect of losartan in Marfan syndrome may be on reduction of blood pressure and dampened mechanosignaling in the aortic root (Lacro et al., 2014; Cook et al., 2014). On the other side, it has been heavily discussed whether the above studied patient cohort had in average an already too advanced disease to be efficiently targeted by losartan. This was also supported by another trial with younger Marfan syndrome patients, which showed a better response to therapy when started at an earlier age and with longer therapy duration (Pees et al, 2013). This demonstrates once again that the actions of TGFβ are context-dependent, and that one approach to target its signaling cannot be generalized, but has to be carefully evaluated for each disease separately.

The therapeutic approach of this trial is to employ losartan to reduce inflammation and progressive scarring that follow skin blistering and abnormal unrestrained wound healing in RDEB. Losartan will target pathological mechanisms downstream of the disease-causing *COL7A1* mutations and the loss of functional collagen VII protein. Thus, it will not aim at a cure, but will offer a low-risk high-benefit symptom-relief therapy.

1.4 Trial purpose and rationale

Dystrophic EB (DEB) is a monogenic skin fragility disorder caused by mutations in the *COL7A1* gene. After defining *COL7A1* mutations and genotype-phenotype correlations in numerous families with RDEB (Has and Bruckner-Tuderman, 2014; Laimer et al., 2015a) and clinical research on EB (Nischler et al., 2009; Wally et al., 2013; Laimer et al. 2015b), we have concentrated on understanding disease mechanisms in RDEB as a basis for novel therapies in the past few years. The collagen VII hypomorphic mouse model, which recapitulates severe human RDEB, including mitten deformities (Fritsch et al., 2008), is an excellent model for such studies. Fibroblast-, iPSC- and MSC-based topical treatments were tested in this model as straight-forward therapeutic approaches (Kern et al., 2009; Wenzel et al., 2014; Kühl et al., 2015), but it became evident that despite successful restoration of collagen VII and its functions locally in

the injected skin areas, the positive effects remained only very localized and of temporary nature. Therefore, it was important to understand the complexity of RDEB at molecular and cellular level.

Role of TGFβ in RDEB: Quantitative proteomics, biochemistry, cell and molecular biology techniques were employed to elucidate the molecular pathology of chronic wounds and progressive soft tissue fibrosis in human and murine RDEB skin and cells. Unbiased global proteomics uncovered new disease mechanisms by showing that loss of collagen VII is associated with a number of proteome changes, including lower abundance of basement membrane proteins and higher abundance of interstitial extracellular matrix components (Küttner et al., 2013, 2014). Several lines of evidence pointed to excessive TGFβ activity in wounded RDEB skin and in RDEB fibroblasts grown under wound-like conditions (Fritsch et al., 2008, Küttner et al., 2013; Nystrom et al., 2013), a reason for excessive fibrosis in tissues. Using different genetic mouse models for RDEB (collagen VII hypomorphic/ conditional knockout mice), we showed that collagen VII is instrumental for skin wound closure by two interconnected mechanisms: it is required both for re-epithelialization via organization of the laminin-332/integrin α6β4 signaling axis that guides keratinocyte migration, and supporting dermal fibroblast migration and cytokine production in the granulation tissue. Loss of collagen VII perturbs both aspects of wound healing, and explains chronic wounds in patients (Nyström et al., 2013).

Role of TGFβ in RDEB-SCC: TGFβ increase also plays an important role in RDEB-SCC development. Our preliminary analysis of patient samples suggested that premalignant changes of the dermal microenvironment drive tumor progression (Mittapalli et al., 2015). Subsequent chemical carcinogenesis in RDEB mice induced invasive tumors phenocopying human RDEB-SCC, whereas wild-type mice developed papillomas. Since chemical carcinogenesis using DMBA / TPA application results in rather homogenous Hras mutations, the different tumor types in control and RDEB mice indicated that the behavior of RDEB-SCC is mutation-independent. Instead, the structural instability of RDEB dermis, combined with repeated injury, led to higher bioavailability of TGFβ which, in turn, promoted extracellular matrix production, increased maturation of fibrillar collagens, cross-linking and thickening of dermal fibrils with the end result of tissue stiffening. The biophysically altered dermis increased myofibroblast activity and integrin-β1/pFAK/pAKT mechanosignaling in tumor cells, demonstrating that in RDEB tumor progression is governed by pre-existing injury-driven changes in the dermal microenvironment. In organotypic RDEB skin cultures, application of inhibitors of TGFβ signaling, lysyl oxidase, or β1-integrin-mediated mechanosignaling, reduced tissue stiffness or its perception and limited tumor cell invasion, thus identifying druggable targets. Several approved drugs already exist for these targets, e.g. losartan to target TGFβ activity and initial fibrotic processes, β-aminopropionitrile or lysyl oxidase antibodies to reduce crosslinking, and small molecule FAK inhibitors to correct mechanosensing in tumor cells and keratinocytes.

Preclinical studies on losartan treatment in RDEB demonstrate high potential for favorable disease modulation: Losartan is an AT1R antagonist that effectively diminishes TGFβ activity by reducing expression of TGFβ ligands, activators and receptors. Given the clear fibrotic component and dysregulation of TGFβ in the pathology of RDEB, we postulated that losartan is effective in delaying disease progression of RDEB. - In recently published work (Nyström et al., 2015) we demonstrated that losartan significantly lowered elevated TGFβ, collagen I, and thrombospondin-1 (a TGFβ activator) expression in human RDEB fibroblasts in vitro. Preclinical evaluation of losartan treatment in RDEB mice unveiled positive therapeutic effects. The treatment was started when the mice were 6.5-week-old. The treatment group was given losartan in drinking water following previous recommendations, and the control group received normal tap

water. The treated group displayed no discomfort or visible signs of adverse effects. The mice were followed for 7 weeks, until significant toe fusion/loss had occurred in the control group, but clearly less so in the treatment group. Longer treatment periods were not allowed due to severe incapacity of the untreated mice caused by the mitten deformities (as decided by the animal research authority). To quantitate the protective effect of losartan, the reduction of the length of the two most pronounced toes on the forepaw was measured over time. The treatment of RDEB mice drastically halted progression of mitten deformities. The predicted average time to complete digit loss was 11.8 ± 0.8 weeks for control group, and 33.6 ± 2.1 weeks for the losartan treated group (see Fig. 1). On the histological and molecular level fibrotic remodeling was significantly less visible and individual markers of dermal fibrosis such as tenascin-C, fibronectin and α-smooth muscle actin were effectively reduced in losartan-treated RDEB mouse forepaws (Nyström et al., 2015).

To gain insight into additional targets of TGFβ signaling inhibition in RDEB we performed global, label-free proteomics on back skin of losartan- vs. vehicle-treated RDEB mice. The analyses revealed that losartan inhibited early fibrotic remodeling and uncovered changes in multiple proteins related to tissue inflammation as losartan targets. In line with this, the degree of inflammation was closely linked to progression of RDEB and variation in disease severity in RDEB mice, and tissue validation showed reduced inflammation and diminished TNFα and IL6 expression in losartan-treated forepaws.

Scarring and progressive fibrosis occur progressively in all DEB forms, leading to the development of pseudosyndactylies in patients with RDEB at very young age. A careful investigation of our RDEB cohort revealed that this starts already around the age of 2 years (data not shown). Our preclinical data in the RDEB mouse model show that losartan reduces and delays development of fibrosis, but it may not reverse already existing severe scarring. Therefore, it is important to start the treatment as early as possible. At this point, the trial is not interesting for adults, but rather for children who have not yet developed severe fusion of fingers or toes, or mitten deformities. This population will profit from therapy the most, and data on efficacy will be most representable. In analogy, in the large clinical trial of atenolol versus losartan in children and young adults with the genodermatosis Marfan syndrome, which is not accompanied by hypertension, the 608 trial participants were above the age of 6 months (Larco et al., 2014).

Taken together, inhibition of TGFβ activity by losartan significantly ameliorates RDEB-specific signs and improves the phenotype in a preclinical model. The estimation is that the delay of mitten deformity formation by a relatively short 7 week-treatment in the preclinical model roughly corresponds to a delay of 2 years in patients with moderate to severe RDEB. Our data also changed the concept of RDEB physiopathology by demonstrating that it is not only a skin fragility disorder, but also a systemic, chronic inflammatory fibrotic disease, which can be ameliorated by anti-inflammatory effects. Thus, it seems feasible that - until a cure becomes possible - losartan can provide urgently needed attenuation of disease progression and serve as a first-in-line disease modulating therapy for RDEB, either as a continuous long-term treatment or as an interval-therapy, to delay fibrotic changes.

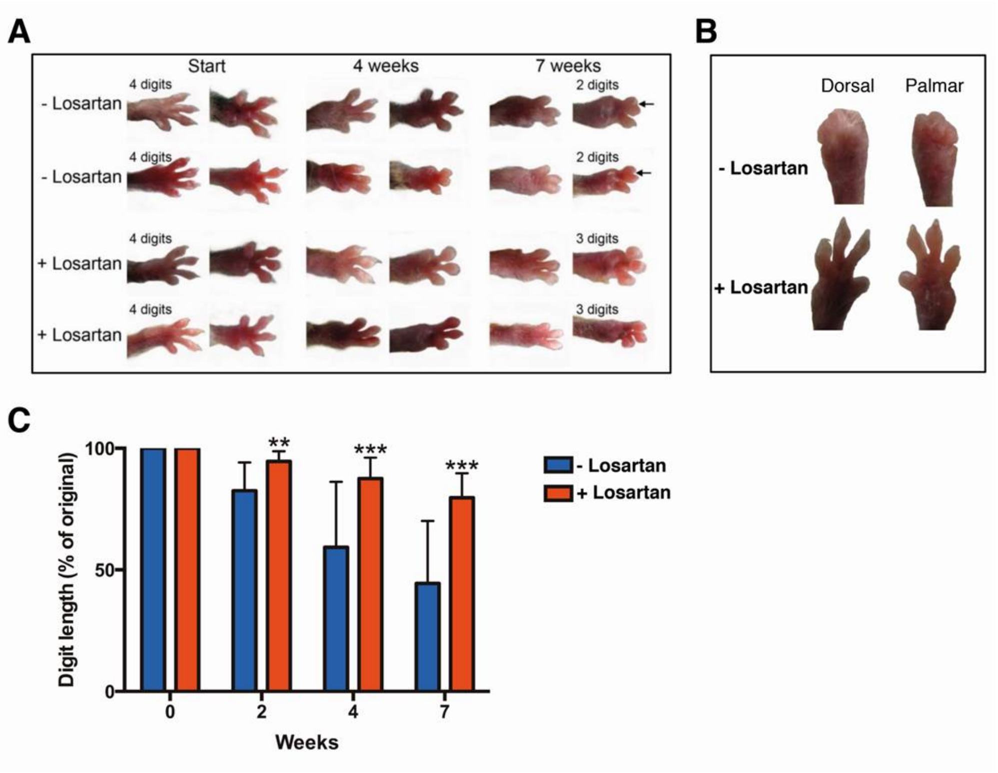

Figure 1 Losartan treatment delays pseudosyndactyly formation in RDEB mice

A, Dorsal and palmar view of the right forepaw of 4 age-matched RDEB mice; the two upper panels: untreated RDEB mice, the two lower panels: RDEB mice treated with 0.6 g losartan per liter drinking water. Shown are photographs at start, after 4 weeks and at the conclusion of the experiment after 7 weeks. The arrows indicate pseudosyndactylies. B, Dorsal and palmar views of the forepaws after 7 weeks treatment +/- losartan. Shown are a good losartan responder and a mouse from the control group with rapid pseudosyndactyly formation, which is fibrosis-driven. C, Bar graph of the relative length of the two most pronounced forepaw digits in age-matched untreated (blue) vs. losartan-treated (red) RDEB mice after 2, 4 and 7 weeks of treatment. The length of digits at start of experiment (0) was set to 100% and length in subsequent measurement expressed as the percentage of this value. Values represent mean ± S.D. *P* values for 2 weeks **P = 0.0021 and for 4 and 7 weeks ***P < 0.001. Figure from Nystrom et al., EMBO Mol Med 2015; figure legend adapted from the publication.

1.5 Rationale for dose selection

The dose of losartan was chosen based on previous studies and FDA recommendations. The maximum FDA recommended dose is 1.4 mg/kg body weight (corresponds to 100 mg/day for an average 70 kg adult). The maximum dose of 100 mg/day will not be exceeded. Importantly, already 50 mg/day (corresponding to 0.7 mg/kg body weight) significantly reduced circulating TGFβ and ameliorated fibrosis in patients with renal or hepatic fibrosis.

1.6 Available treatments for RDEB

No cure exists for RDEB, and the unmet medical need remains very high. So far, different gene-, protein- and cell-based therapy strategies were examined, with the aim to replace the missing or

non-functional collagen VII. Some of them showed promise at the in vitro or the preclinical level and led to small pilot trials, in particular cell-based therapies. Bone marrow transplantation showed modest and transient clinical benefits in some individuals, but the risk-benefit ratio remained very high, and several patients succumbed to complications of the procedure. Topical injections of allogeneic fibroblasts facilitated wound healing in some, but not all patients, and painfulness of the injections was a major limitation of this approach. Systemic infusions of allogeneic bone marrow derived mesenchymal stromal cells (MSC) reduced itch and promoted general well-being in single individuals, but did not increase collagen VII in the skin. Thus, in general, efforts to achieve safe and effective causal treatments still face substantial hurdles and their clinical implementation remains elusive. Additionally, the cost of these curative therapies for RDEB is not yet known and will vary depending on the type and method. In any case, based on the experiences so far, the cost for GMP-quality therapeutics is expected to be very high. Thus far, the cost of the pilot therapies has ranged from a few thousand euros for cell injections (fibroblasts or MSC) to several hundred thousand euros for bone marrow transplantation.

So far standard treatment modalities for all patients include antiseptic, anti-inflammatory wound and finger separating dressings, symptomatic therapies to compensate nutritional deficiencies, numerous checkups in various disciplines like pediatrics, dentistry, gastroenterology, nephrology, cardiology and osteology as well as dermatology and physiotherapeutic support to stimulate the development of the child. In spite of these extraordinary efforts the clinical course remains deteriorating.

1.7 Risk-Benefit assessment

In RDEB, life-long skin fragility and multi-organ involvement cause a tremendous disease burden. Soft tissue scarring follows skin blistering and wound healing. The recurrent blistering is also associated with corneal and esophageal scarring, resulting in vision impairment and esophageal strictures. Among the many symptoms, joint contractures and mitten deformities of hands and feet cause severe disability. The fibrosis of the dermis increases tissue stiffness and favors development of squamous cell carcinoma (SCC) in adult age, a severe disease complication. No cure exists for RDEB, and the unmet medical need remains very high as the symptoms get worse continuously with advancing age. Therefore the individual load situation of patients is expected to be high that additional exacerbating factors need to be carefully considered.
The benefit of this potential symptom-relief therapy with losartan is that - although not a cure and after verification of safety and tolerability - it has a high potential to reduce inflammation and fibrosis and thereby benefits patients by reducing pain and itch and if possible increases functionality of hands, feet and other tissues and simultaneous improves quality of life significantly. The development of mitten deformities, joint contractures and esophageal stenosis is expected to be delayed;. Finally, a number of people with RDEB suffer from heart problems (dilated cardiomyopathy). We expect that losartan possibly has a positive effect on the heart in these individuals andplays a preventive role in other patients with RDEB in future.

The risks are low: Losartan is commonly used to treat high blood pressure and is in general a safe drug, also in children, but we will carefully monitor blood pressure and heart rate of the trial participants. In the recently published report on the use of losartan in Marfan syndrome, Larco et al. showed that the blood pressure was on average only 1-2 % lower than before treatment. Some participants reported mild side effects, such as headache, fatigue, or muscle cramps. Only

fewer than 5% had bothersome chest pain or dizziness. Since losartan´s target, the angiotensin-renin system, is important for embryonic development, pregnant women should not take the drug due to the risk of fetal toxicity. An obvious advantage of this small molecule drug is that in the event of unexpected adverse events the drug administration can be discontinued and the compound allowed to clear. The half-life of losartan and its main metabolite is estimated to be approximately 6 hours, meaning that > 90 % of the drug is eliminated from the body within 24 hours after the medication has been discontinued. Although losartan has been approved by the FDA as an antihypertensive drug for children >6 years of age, it is mentioned in the “Fourth report on the Diagnosis, Evaluation and Treatment of High Blood Pressure in Children and Adolescents” by the U.S. Department of Health and Human Services for the treatment of hypertension already at the age of 1 year. Additionally, clinical trials have been published with losartan used for hypertensive children aged> 6 months and losartan was well-tolerated at a dosage up to 1.4 mg/kg per day (Webb et al., 2014).
Use of a small molecule such as losartan can therefore generally be viewed as safer and easier to dose and control than e.g. gene-, cell-, and protein-based therapy approaches which development to broad safe clinical application is expected to take years.

The immediate availability, easy and safe use, and low cost of losartan therapy represent substantial advantages when compared to curative therapy approaches for RDEB.
During losartan therapy exacerbating factors will be regularly checked on a daily and weekly base to verify the impairment of every days life of the patients. The diary logs of the patients or parents will be checked by study nurses and trial physicians during telephone visits.

1.8 Pharmacoeconomics

We expect losartan to be beneficial in reducing the need for topical therapies, as well as pain and itch medication for EB patients. Additionally, low cost of losartan therapy might represent a substantial advantage when compared to therapy approaches for RDEB treatment that are currently being developed. We expect that the hospitalizations will be reduced due to the Losartan-treatment.

During the trial, the number of nights in hospital during 9 months before and after the 9 months treatment with losartan will be registered in order to reveal a possible positive effect of losartan on frequency/duration of hospitalizations.

2 Objectives and endpoints

Table 1 Objectives and related endpoints

	Objective	Endpoint
**Primary Safety**	Establish tolerability and safety of losartan in children with moderate to severe RDEB.	Occurrence of a serious safety concern, defined as one of the following side effects of losartan:<br>1) clinically relevant severe hypotension (for definitions see section 2.1);<br>2) immediate hypersensitivity reactions to the drug<br>3) clinical relevant severe hypo- und hyperkalemia (for definitions see section 8.3.1);

	Objective	Endpoint
**Secondary Safety**		(Serious) adverse events, evaluated by monitoring heart rate and function and blood pressure, using echocardiography, home blood pressure monitoring devices, and blood tests throughout the study.
**Secondary Efficacy**	Obtain first information on the efficacy of losartan in improving the disease manifestations and quality of life, and reducing inflammation and fibrosis in moderate to severe RDEB over a period of 9 months	Efficacy will be assessed using validated scoring systems for the clinical manifestations of RDEB :<br>• Physician Global Assessment (PGA)<br>• Birmingham Epidermolysis Bullosa Severity Score (BEBS)<br>• Epidermolysis Bullosa Disease Activity and Scarring Index (EBDASI)<br>• Score of Colville and Terrill<br>• Our own morphometric scoring instrument of pseudosyndactyly progression<br>• Mayo Dysphagia Questionnaire-day 30 (MDQ-30)<br>• Itch Assessment Scale for the Pediatric Burn Patients<br>• Wong-Baker FACES Scale for Pain<br>• Quality Of Life in EB (QOLEB) questionnaire<br>• Children’s Dermatology Life Quality Index (CDLQI)
	Assessment of fibrotic and inflammatory markers in skin and blood over a period of 9 months	• Efficacy will be assessed using fibrotic and inflammatory markers:<br>• TGFβ activity in skin as per staining for TGFβ protein, TGFβ receptor II and pSMAD2/3<br>• Inflammatory markers in skin as per staining for CD3 (mature T-cells), CD4 (mainly T-helper cells), CD8 (mainly cytotoxic T-cells), CD68 (monocytes/macrophages), TNFα and IL6<br>• Dermal fibrosis as per histology, H&E staining and picrosirus red staining<br>• TGFβ levels in serum as per TGFβ-ELISAs<br>• Inflammatory markers in serum as per TNFα-, CRP-, ferritin-, IL6-, amyloid-ELISAs<br>• Fibrotic markers in serum as per ELISAs for procollagen I amino-terminal peptide (PINP) and procollagen III amino-terminal peptide (PIIINP)<br>For details see Appendix 12 - Appendix 14

Additional aim of the study is to assess the suitability of questionnaires / scoring instruments used in this trial and the study procedure for future multi-center trials on efficacy of losartan. In addition, the frequency / number of nights in hospital during the 9 months before and after the first losartan dose will be collected.

2.1 Primary objective and endpoint

The primary endpoint is tolerability and safety of losartan in children with RDEB, meaning that our primary interest is to detect possible adverse drug reactions of losartan. As severe complications resulting in a serious safety concern we define the following side effects of losartan:

1) clinically relevant severe hypotension, i.e. the patient experiences continuous dizziness and headaches owing to the low blood pressure, leading to interruption of study medication;
2) immediate hypersensitivity reaction to the drug;
3) clinical relevant severe hypo- und hyperkalemia (for definitions see section 8.3.1)

The tolerability of losartan will be evaluated by monitoring heart rate and function using electrocardiography and echocardiography at every clinic visit, as well as daily blood pressure measurements at home recorded in a patient diary and evaluated during all visits. Additionally, laboratory tests will be done at every clinic visit (for details see section 7.6.9).

3 Clinical trial plan

3.1 Trial design

This is a prospective, open, single-arm phase I/II clinical study with the primary objective to establish safety and tolerability of losartan in moderate to severe RDEB. A secondary objective is to obtain first information on the efficacy of losartan in improving the disease manifestations and quality of life, and reducing inflammation and fibrosis in moderate to severe RDEB. Efficacy will be assessed in comparison to their own baseline values before start of the treatment. Baseline can be regarded as adequate control at this stage, since a spontaneous improvement of inflammation, fibrosis and mitten deformities cannot be expected, and improvement can be regarded as treatment success.

3.2 Treatment arms

This is a single arm study. All patients will receive losartan.

3.3 Treatment duration

Treatment duration with losartan comprises 10 months, encompassing 4 months (ca. 16 weeks) up-dosing of losartan, 5 months (ca. 24 weeks) full dose losartan (final target dose of 1.4 mg/kg), and 1 month (ca. 4 weeks) losartan tapering.

Patients will continue on therapy with oral losartan until discontinuation due to intolerable toxicity, withdrawal of consent, death or termination of the trial.

3.4 Trial timetable

Enrolment of first patient (FPFV)	1st quarter 2017
Enrolment of last patient (registration)	3rd quarter 2018
End of trial for last patient (LPLV)	4th quarter 2019
Final statistical analysis	2nd quarter 2020
Treatment duration and follow up per patient	10 months treatment followed by<br>3 months follow up
Planned interim analysis	Not applicable

3.5 Participating sites

Two sites are planned: 1 in Germany (EB Center Freiburg) and 1 in Austria (EB House Austria, Salzburg). Both sites meet the structural and personnel requirements for performing the planned regular trial-related investigations. The Centers are geographically close, and since both are in German-speaking countries, there will be no language barrier between the patients and trial personnel. The Centers have collaborated since 2003 within the German network of excellence “EB-Network” (www.netzwerk-eb.de).

3.6 Number of patients

The bi-center trial will enroll 30 children with moderate to severe RDEB (age 2 – 16 years, starting from the 25th month of life).

3.7 Recruitment strategy and planned rate

The number of 30 children with RDEB to be enrolled is feasible, since approximately 150 patients with molecularly confirmed RDEB in the age range of 2-16 years are included in the EB registries in Freiburg and Salzburg. We have already contacted 25 families and 24 were interested in participating in the study. We will work intensively with the German and Austrian EB patient organizations (DEBRAs), to notify as many people as possible about the trial per webpages, newsletter, email and social media. Nevertheless, in case of difficulties in enrolling a sufficient number of patients in one EB Center, the other Center will enroll more patients to reach the goal of 30 trial participants. We will also approach the more than 20 clinicians and hospitals associated with the German EB-network and the EB House Austria in order to recruit new individuals with EB, who may not have been included in the EB registries so far. Furthermore, the diagnostic units at both EB Centers continually disclose *COL7A1* mutations in new individuals, about 10 cases in Freiburg and 2-4 cases in Salzburg each year, and we can identify new potential trial participants among these individuals. Finally, if necessary, we will extend the recruitment period for 6 more months.

4 Trial population and selection criteria

4.1 Target population / main diagnosis

4.1.1 Target population

Patients from 2 to 16 years (starting from the 25th month of life) with molecularly confirmed diagnosis of moderate to severe RDEB of both genders will be enrolled into this trial. Patients will only be allowed to enter the trial if they and/or their parents or legal guardians provide written informed consent about their participation (following full explanation of the trial) and if the physician has verified that the patient meets all of the inclusion criteria and none of the exclusion criteria.

4.1.2 Gender distribution

No gender ratio has been stipulated in this trial as the results of the preclinical and clinical studies did not indicate any difference in the effect of the trial treatment in terms of efficacy and safety.

4.2 Inclusion criteria

Patients eligible for inclusion in this trial must meet all of the following criteria:

1. Written informed consent of parents or legal guardians obtained according to international guidelines and local laws;
2. Patient's assent (if applicable according to patient's age and understanding);
3. Male or female patients from 2 to 16 years (age of > 25 months);
4. Molecularly confirmed diagnosis of moderate to severe RDEB. If the patient is completely collagen VII-deficient, as shown by negative collagen VII immunofluorescence staining of a skin biopsy, no genetic confirmation of the diagnosis will be required for inclusion in the study. In case of residual collagen VII expression, the *COL7A1* gene will be analyzed for mutations, to confirm the diagnosis of RDEB;
5. Ability of the patient (if applicable according to patient's age and understanding), parents or legal guardians to understand the nature of the trial and trial-related procedures and to comply with them;
6. Able to travel to trial site for all clinic visits;

4.3 Exclusion criteria

Patients eligible for this trial must **not** meet any of the following criteria:

1. Simultaneous or previous participation in any interventional trial within the past 3 months before entering this trial; participation in simultaneous registry and diagnostic trials during the trials is allowed;
2. Anemia with hemoglobin < 8 g/dl;
3. Hypotension (defined as age-related systolic blood pressure under the 5th percentile);
4. Cardiologic contraindications, such as severe heart failure with ejection fraction < 35%;

5. Patient requires any medications that are likely to cause interactions with losartan, e.g. rifampicin, ACE-inhibitors;
6. Renal artery stenosis or renal insufficiency with creatinine clearance < 30 ml/min;
7. Severe liver failure;
8. Severe, untreated electrolyte disturbances;
9. History of cancer or chronic viral infections (HBV, HCV, HIV);
10. Hypersensitivity to losartan or any of the excipients;
11. Known or persistent abuse of medication, drugs or alcohol;
12. Persons who are in a relationship of dependence/employment with the sponsor or the investigator.
13. Current pregnancy or nursing period;
14. For female patients with menarche: unwillingness to use adequate contraception or to stay sexually abstinent during the course of the trial;

Women with menarche can only take part in this trial if the risk of becoming pregnant is absolutely minimized. Save contraception methods comprise: a) combined oral estrogen and progestogen containing hormonal contraception or other hormonal contraceptions in combination with a mechanical method of contraception, or female condoms, diaphragm or coil, each used in combination with spermicide or intra-uterine device. b) sexual abstinence. Sexual abstinence is considered a highly effective method only if defined as refraining from heterosexual intercourse during the entire period of risk associated with the study treatments (Recommendations related to contraception and pregnancy testing in clinical trials, Clinical Trial Facilitation group, 2014).

5 Enrolment and patient registration

5.1 Patient eligibility

If a patient appears to be eligible for the trial, the investigator will inform the patient/ legal guardians about the trial and ask the patient/ legal guardians for the written consent. It is imperative that written consent is obtained prior to any trial-specific procedures. The investigator will then record the details of these trial patients on the following trial-specific lists:

- **Subject Screening log**: for the documentation of the trial patients who were checked for eligibility before the clinical trial. The following will be entered: the identification code, the dates of written consent, screening and details on whether and when the patient was registered in the trial and, if not, the reason for not enrolling the patient.

- **Subject identification log**: A confidential log of the names of all trial patients with the identification code assigned to each patient at the time of enrolment in the clinical trial. With this list, the identity of each patient can be revealed. The list must be kept confidential and must not leave the institution. It must remain at the trial center and must not be copied or otherwise passed on monitors, auditors and representatives of authorities must be allowed to inspect the list on request.

Patient identification code is a unique trial-specific identification number which identifies the patient and consists of two parts: one corresponds to the site number and the second stands for a consecutive number of the patient enrolled at a particular site, so that each patient is numbered uniquely across the entire database.

5.2 Patient registration

The patient identification code assigned for the trial will be entered on the registration form and the questions on a birth date (month and year), age range (2-5, 6-16 years), inclusion/exclusion criteria, and whether patient's study card/ diary/ blood pressure monitoring devices handed out (yes/no) and sex on the form will be answered. The fully completed form will then be faxed to the central trial office (CTU) for registration:

Clinical Trials Unit
Medical Center - University of Freiburg
Fax: +49 761 270-74 390

Registration times:
Monday to Friday from 9:00 to 16:30

The CTU will review the patient's details on the registration fax and then confirm the patient's enrolment in the trial by fax. The treatment can be initiated according to the protocol.

The coordinating investigator will include and treat children between 2 and 5 years only after prior enrolment of 5 children between 6 and 16 years, which did not show any safety concerns until phone Visit 2 (week 4).

6 Treatment plan and procedure

The investigator will instruct the patient and the patient's parents/ legal guardians to take the IMP as per protocol at every clinical visit based on a demo medication kit A dose change takes only place at the clinical visits All dosages prescribed and dispensed to the patient and any dose change or interruption must be recorded in patients chart and paper CRFs, as appropriate.

The trial visits are delineated below:

Clinic visit: For a clinic visit, the trial participant travels to the trial site. The visits will be organized and coordinated by the study nurse and include assessment of safety and tolerability, physical examination by the trial physicians (dermatology and cardiology) and collection of adverse events, laboratory tests and skin biopsy, concomitant medication and EB scores as described in sections 7.3 and 7.4.

Home visit: For the home visit, the study personnel (trial physician or study nurse) travel to patients' home for physical examination, collection of adverse events, concomitant medications, verification of exacerbating factors and EB scores as described in sections 7.3 and 7.4.

Phone visit: The study nurse/person designated by the investigator will interview the patient by phone regarding drug tolerability (AEs), blood pressure and pulse rate values, weight modifications, changes in concomitant medication and verification of exacerbating factors. The

data collected by the study nurse will be reviewed by trial physicians (dermatology and cardiology). Based on these data, the investigator will state the dose to be taken and the study nurse will inform the patient/ patient's parents/ legal guardians correspondingly.

If the higher dose was not tolerated, losartan will be reduced to the highest tolerated dose. If no adverse events occur, the patient will continue taking this dosage, until the end of the treatment phase.

Measures at home: To ensure continuous blood pressure and pulse rate monitoring, home blood pressure monitoring devices will be distributed to every trial participant, together with a diary for recording the blood pressure and pulse twice daily. Possible symptoms that could represent an adverse event will also be recorded, and the trial participant will be asked to inform the EB Center immediately in such a case. Patient's weight will be measured on day preceding each phone visit.

6.1 Dosing regimen and investigational medicinal product administration

Figure 2 Dosing visit flowchart

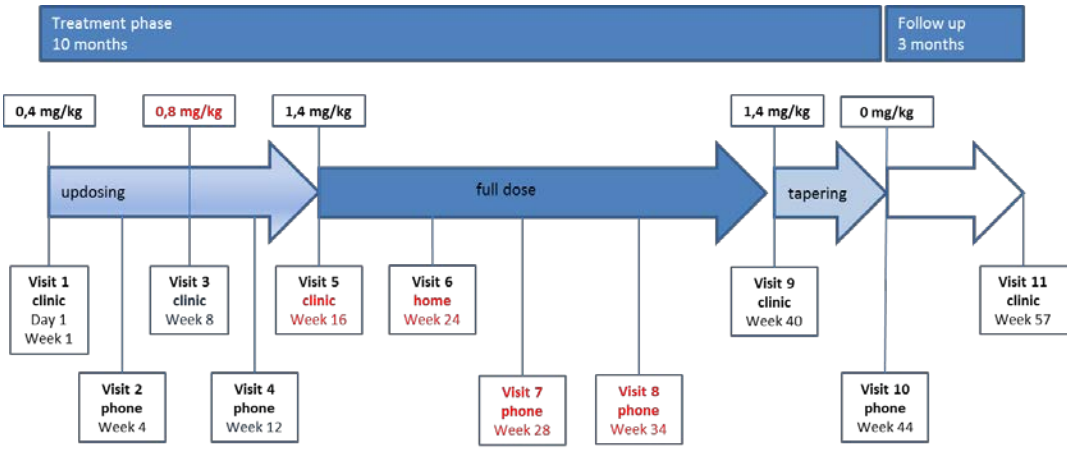

The first dose of losartan (0.4 mg/kg) will be given in a day-clinic setting (about 8 hour stay in the hospital) with careful monitoring by the pediatric cardiologists (Visit 1- clinic). If no adverse effects occur the patient will continue taking losartan at this dose for 4 weeks. At this point, a phone visit will take place to interview the patient/ parents/ legal guardians about her/his well-being (Visit 2- phone). The patient will then visit the respective trial site for clinical assessment at week 8 (Visit 3- clinic), and the losartan dose will be raised to 0.8 mg/kg for 8 weeks. At week 12 (Visit 4- phone), the patient/ parents/ legal guardians will be interviewed by phone. At week 16 (Visit 5- clinic) the patient will visit the respective trial site for clinical assessment. During this visit, the losartan dose will be raised to the final target dose of 1.4 mg/kg, which the patient will take for 24 weeks in total. At week 24 the patient will be visited at home by the study personnel (trial physician or study nurse) (Visit 6- home). At week 28 and 34 the patient/parents/legal guardians will be interviewed by phone again (Visit 7- phone and Visit 8- phone). The final target dose of losartan will be continued until week 40, when the patient will be subjected to a thorough dermatological and cardiological investigation in a day-clinic setting (Visit 9- clinic; about 8 hours in the hospital). During the next 4 weeks losartan will be tapered slowly to 0 mg/kg, in order to avoid adverse effects from drug withdrawal. At week 44, a phone interview will take place (Visit

10- phone), and after a follow-up period, the last clinic visit will be at week 57 (Visit 11- clinic) to ensure that no adverse events have arisen after completion of the therapy regime.

Table 2 Treatment schedule

Treatment phase	Daily losartan dose [mg/kg]	Pharmaceutical form and route of administration	Daily* administration morning-midday-afternoon	Duration in weeks	Start of dose after corresponding Study Visit **
**Up dosing**	0,4	Oral	0-0-1	8	Visit 1 (clinic) Day 1
	0,8	Oral	0-0-1	8	Visit 3 (clinic) Day 56 (week 8)
**Full dose treatment**	1,4	Oral	0-0-1	24	Visit 5 (clinic) Day 112 (week 16)
**Dose tapering**	1,12	Oral	0-0-1	1	Visit 9 (clinic) Day 280 (week 40)
	0,84	Oral	0-0-1	1	
	0,56	Oral	0-0-1	1	
	0,28	Oral	0-0-1	1	

* Since blood pressure reduction is not the aim of this study, losartan suspension will be administered orally once daily at night; the total daily dose will not exceed 100 mg.

** Dose escalation will be only performed in case of no adverse reactions reported at clinic visits or phone.

6.2 Dose modification and dose delay

If a patient does not tolerate the final target dose of losartan she/he will continue taking the highest tolerated dose until completion of the trial, and the secondary endpoints will be evaluated based on that dose. The dose adjustment has to be previously discussed with the Coordinating Investigator unless it concerns patient's safety.

IMP intolerance is defined as follows:

Cardiovascular parameters: if at the first clinical visit with intake of the IMP or with up-dosing of the IMP the blood pressure is lowered >25% compared to the initial value, or symptoms like dizziness, signs of peripheral shutdown, tachycardia) occurs, the patient has to be monitored for at least 3 hours. If there is no normalization of the mentioned parameters, no intake or up-dosing of the IMP will occur.

In case one dose was forgotten, the next intake of the IMP has to take place as scheduled on the next day.

All dose changes or interruptions must be recorded in the patient's diary and on the appropriate CRF pages.

6.3 Permitted concomitant treatment/medication

The patient/ parents/ legal guardians must notify the investigational site about any new medications he/she takes after the start of the trial medication. All medications (other than IMP) and significant non-drug therapies (including physical therapy and blood transfusions) administered after the patient starts treatment with IMP must be listed in the CRF.

Administration of the following medications is acceptable and will not result in the withdrawal of the patient from the trial:

- Systemic or topical antihistamines
- Systemic or topical antibiotics used for less than 3 months continuously
- Systemic corticosteroids used for less than one month continuously
  For details on topical corticosteroids see section 6.4
- Immunosuppressive agents used for less than one month continuously
- Non-steroidal anti-inflammatory drugs (NSAIDs)
- Morphine or other narcotic pain relievers
- Vitamins

6.3.1 Permitted rescue medication

Patients should receive treatment/medication appropriate to their clinical condition in an emergency. In case of emergency the patients will receive standard care in the hospital.

6.4 Prohibited concomitant therapy

During the trial the patient is not permitted to use any medications that are likely to cause interactions with losartan, e.g. rifampicin, ACE-inhibitors. If such agents are required for a patient, the patient discontinues the trial treatment and the given prohibited therapy has to be recorded in the CRF.

The use of topical corticosteroids is not permitted at the biopsy sites (hands or feet) one month before Visit 1 and 9, where the skin biopsies will be taken.

6.5 Hormonal contraception allowed

The use of oral contraceptives and implantable hormonal contraception is allowed during the trial (see exclusion criteria section 4.3).

6.6 Unblinding of treatment assignment

Not applicable.

7 Visit schedule and assessments

7.1 Flow and visit schedule

A detailed Flowchart (visit schedule and assessments) is provided in the synopsis (see page 17). The schedule of assessment lists all of the assessments and indicates with an “X” the visits when they have to be performed. All data obtained from these assessments must be supported in the patient’s source documentation.

7.2 Visit and assessment windows

Screening evaluations have to be performed within 14 days prior to the first dose of trial treatment.

During the course of the trial visits and test procedures should occur on schedule whenever possible; visits that occur ± 7 days from the scheduled date will not constitute protocol deviation.

7.3 Screening and registration

The investigator is obliged to give the patient thorough information about the trial and the trial related assessments, and the patient and parents or legal guardians should be given ample time to consider his or her participation. The investigator must not start any trial specific procedure before Informed Consent Form (ICF) is signed and dated by both parents or legal guardians (as well as patient if applicable) and investigator.

7.3.1 Pre-screening (optional)

As patients with orphan diseases are geographically dispersed, it is intended to collect the following pre-screening data prior to patient’s assigning to one of the study sites in order to avoid needless patients’ trip:

- Serology regarding HBV, HCV, and HIV
- Hemoglobin value

These data will not be collected in the CRF. The patients/ parents/ legal guardians will fax the results of the above examinations to the study site approximately 3 days prior to the planned screening date.

Details on in informed consent procedure prior to patient’s inclusion into the study are provided in section 15.3.

7.3.2 Screening

After the patients/ parents/ legal guardians having been informed about the trial and after having given their written informed consent, patients have to pass the examinations listed in section 7.3.4 prior to registration. Results of examinations routinely performed due to manifestations of EB will be accepted, if they were done within 2 weeks prior to registration, except for serology tests which do not have to be repeated if they were done within 4 weeks prior to registration.

Patients must meet all inclusion criteria and none of the exclusion criteria to be considered eligible and will be registered to the trial as described in section 5.2.

If screening and Visit 1 are done on the same day, all screening evaluations have to be done before the registration. Only after these are completed and the patient is registered the administration of IMP can follow.

7.3.3 Data to be collected on screening failures

Screening failures are defined as patients with the signed ICF who failed to be registered in the study for any reason. These patients have to be documented on the subject screening log (see section 5.1). For these patients the screening CRFs and CRF pages with inclusion/exclusion criteria have to be completed.

7.3.4 Assessments at screening (Day -14 until day -1)

The data that will be collected at screening include the following (please refer to section 7.6 for a precise definition of assessments):

- Informed consent
- Verification of inclusion / exclusion criteria
- Demographic data
- Medical history
- Urine pregnancy test, if applicable
- Serology (HBV, HCV, HIV), if older than 4 weeks prior to registration
- Hemoglobin
- Physical examination
- Height
- Weight
- Heart rate and blood pressure
- Documentation of fibrosis and disease burden (scores)
- Morphometric pseudosyndactyly scoring (central)
- Laboratory tests (see Flowchart)
- Markers of inflammation and fibrosis in blood
- Electrocardiogram (ECG)
- Echocardiography
- Itch, pain and QOL scores
- Exacerbating factors
- Concomitant medication

7.3.5 Registration (Clinic-Day 1)

Patients considered eligible by the investigator once all screening procedures are complete will be registered to the trial (see section 5.2).

7.4 Treatment phase

Following registration and initiation of trial treatment (Visit 1), the patient should visit the trial site at weeks 8, 16, 40 and 57. Phone visits will take place at weeks 4, 12, 28, 34 and 44. For details see sections below. The home visit takes place at week 24.

7.4.1 Visit 1 (Clinic- Day 1)

In addition to application and dispensation of the IMP the following assessments have to be performed:

- Hemoglobin (Hb)
- Physical examination
- Heart rate and blood pressure
- Skin biopsy
- Concomitant medication
- Urine pregnancy test (if applicable)

If the time elapsed between screening visit and Visit 1 is longer than 7 days, the following assessments have to be repeated:

- Weight
- Documentation of fibrosis and disease burden (scores)
- Laboratory tests (see Flowchart) including urine pregnancy test (if applicable).
- Markers of inflammation and fibrosis in blood
- Electrocardiogram (ECG)
- Echocardiography
- Itch, pain and QOL scores
- Exacerbating factors

7.4.2 Visit 2 (Phone- Week 4)

The following assessments have to be performed (for details on assessments see section 7.6):

- Weight
- Heart rate and blood pressure check (as documented in patient diary)
- Concomitant medication
- Adverse Events
- Exacerbating factors

7.4.3 Visit 3 (Clinic- Week 8)

The following assessments have to be performed, in addition to application and dispensation of the IMP (for details on assessments see section 7.6):

- Urine pregnancy test, if applicable
- Hemoglobin (Hb)
- Physical examination
- Height
- Weight
- Heart rate and blood pressure
- Documentation of fibrosis and disease burden (scores)
- Morphometric pseudosyndactyly scoring (central)
- Laboratory tests (see Flowchart)
- Markers of inflammation and fibrosis in blood
- Electrocardiogram (ECG)
- Echocardiography
- Itch, pain and QOL scores
- Exacerbating factors
- Concomitant medication
- Adverse Events

If the drug is tolerated, the losartan dose will be raised to 0,8 mg/kg.

7.4.4 Visit 4 (Phone- Week 12)

The following assessments have to be performed (for details on assessments see section 7.6):

- Weight
- Heart rate and blood pressure check (as documented in patient diary)
- Exacerbating factors
- Concomitant medication
- Adverse Events

7.4.5 Visit 5 (Clinic – Week 16)

The following assessments have to be performed, in addition to application and dispensation of the IMP (for details on assessments see section 7.6):

- Urine pregnancy test
- Hemoglobin (Hb)
- Physical examination
- Height
- Weight
- Heart rate and blood pressure
- Documentation of fibrosis and disease burden (scores)
- Morphometric pseudosyndactyly scoring (central)
- Laboratory tests (see Flowchart)
- Markers of inflammation and fibrosis in blood
- Electrocardiogram (ECG)
- Echocardiography
- Itch, pain and QOL scores

- Exacerbating factors
- Concomitant medication
- Adverse Events

If the drug is tolerated, the losartan dose will be raised to the final target dose of 1.4 mg/kg.

7.4.6 Visit 6 (Home- Week 24)

The following assessments have to be performed (for details on assessments see section 7.6):

- Weight
- Heart rate and blood pressure check (as documented in patient diary)
- Concomitant medication
- Adverse Events
- Exacerbating factors

7.4.7 Visit 7 (Phone- Week 28)

The following assessments have to be performed (for details on assessments see section 7.6):

- Weight
- Heart rate and blood pressure check (as documented in patient diary)
- Exacerbating factors
- Concomitant medication
- Adverse Events

7.4.8 Visit 8 (Phone- Week 34)

The following assessments have to be performed (for details on assessments see section 7.6):

- Weight
- Heart rate and blood pressure check (as documented in patient diary)
- Exacerbating factors
- Concomitant medication
- Adverse Events

7.4.9 Visit 9 (Clinic- Week 40)

The following assessments in addition to application and dispensation of the IMP have to be performed (for details on assessments see section 7.6):

- Urine pregnancy test, if applicable
- Hemoglobin (Hb)
- Physical examination
- Height
- Weight
- Heart rate and blood pressure
- Documentation of fibrosis and disease burden (scores)
- Morphometric pseudosyndactyly scoring (central)

- Laboratory tests (see Flowchart)
- Markers of inflammation and fibrosis in blood
- Electrocardiogram (ECG)
- Echocardiography
- Skin biopsy
- Itch, pain and QOL scores
- Exacerbating factors
- Concomitant medication
- Adverse Events

After this visit, the losartan dose will be tapered to 0 mg/kg within 4 weeks (each week tapering of 1/5 of the dose, in order to avoid adverse effects from drug withdrawal, for details see section 6.1).

7.4.10 End of treatment (EOT) visit (Phone- Week 44)

The following assessments have to be performed (for details on assessments see section 7.6):

- Weight
- Heart rate and blood pressure check (as documented in patient diary)
- Exacerbating factors
- Concomitant medication
- Adverse Events

After this visit, the IMP will be discontinued.

7.5 End of study (EOS) visit (Clinic- Week 57)

At final visit the following assessments have to be performed (for details on assessments see section 7.6):

- Hemoglobin (Hb)
- Physical examination
- Height
- Weight
- Heart rate and blood pressure
- Documentation of fibrosis and disease burden (scores)
- Morphometric pseudosyndactyly scoring (central)
- Laboratory tests (see Flowchart) including urine pregnancy test
- Markers of inflammation and fibrosis in blood
- Electrocardiogram (ECG)
- Echocardiography
- Itch, pain and QOL scores
- Exacerbating factors
- Concomitant medication
- Adverse Events (see section 10.1.2)
- Occurrence of squamous cell carcinoma during the study (yes/no)

7.6 Description of examinations during the trial

7.6.1 Patient demographics

Demographic data that will be collected on patient characteristics at screening include: date of birth (month and year), sex, and childbearing potential.

7.6.2 Medical and RDEB disease history

At screening relevant past medical history including the molecularly-confirmed diagnosis (moderate or severe generalized RDEB), and assessments of any further current medical condition have to be documented in the CRF.

7.6.3 Pregnancy test

All female patients of childbearing potential must undergo a urine pregnancy test at screening to confirm eligibility in the trial. Additional pregnancy tests are described in the schedule of assessments.

In case of pregnancy, the patient must immediately be withdrawn from the trial, and the pregnancy must be reported to the sponsor on the appropriate form.

7.6.4 Physical examination

Thorough physical/medical examination includes, but is not limited to skin, cardiovascular, gastrointestinal, hepatobiliary, respiratory, musculoskeletal, genitourinary/renal and other organ systems.

Physical examinations are recommended to be performed according to the flowchart; relevant findings concerning these examinations will only be documented in the CRF at screening. At other visits in case of clinically relevant abnormal findings, the investigator has to document an AE (please refer to section 10.1.1 for definitions) on the corresponding CRF-page.

7.6.5 Height and weight

Height (cm) and weight (kg) must be taken at screening and all clinic visits, weight additionally the day before the phone visits take place. Results must be present on the patient’s chart and recorded correspondingly onto the CRF pages.

The body weight has to be checked at clinic visits, preferably prior meal; at home measurement of weight has to be performed in the morning and without clothes, on days preceding the phone visits. The weight taken at home and its modification will be communicated to the study nurse at phone visits in order to adjust the IMP dose.

7.6.6 Pulse rate and blood pressure

Pulse rate and blood pressure will be measured at every clinic visit according to the National Institutes of Health, National Heart, Lung, and Blood Institute Guidelines [NIH 1997] with the following standardized techniques:

- Patients are seated in a chair; blood pressure measurement begins after at least 5 minutes of rest,
- The appropriate cuff size is used to ensure accurate measurement,
- Only one reading is required.

Measurements of blood pressure and pulse rate will also take place at home in the morning and afternoon with blood pressure monitoring devices that will be provided by the trial centers and will be suitable for children. The blood pressure and pulse rate will be measured at home BID and recorded in the patient diary. The data of the diary will be evaluated at each clinic and phone visit.

7.6.7 Documentation of fibrosis and disease burden (scores)

The following data will be collected by the investigator at clinic visits:

- **The Physician Global Assessment (PGA)**, a ten-point visual analog scale ranging from 0 (perfect health) to 10 (worst skin condition imaginable). This validated score allows physicians to rate disease activity by their overall impression of the patient's condition. It has been successfully used for inflammatory dermatoses such as psoriasis, eczema or dermatomyositis. Since there is no gold-standard for assessing general RDEB activity, we will include the PGA expecting its positive correlation with the two EB-specific scores below. For details on PGA see Appendix 2.

- **The Birmingham Epidermolysis Bullosa Severity Score (BEBS)**, a validated severity score for all subtypes of EB at all ages that was shown to be simple, valid, sensitive and reliable. Eleven items are scored: area of damaged skin, involvement of nails, mouth, eyes, larynx and esophagus, scarring of hands, skin cancer, chronic wounds, alopecia and nutritional compromise. The area of damaged skin is allocated 50 points, and the 10 other items 5 points each, giving a maximum score of 100. The BEBS does not differentiate between active lesions and chronically damaged skin, and it has not yet been shown to detect changes with treatment. Therefore, an additional EB-specific scoring instrument will be used. For details see Appendix 3.

  All investigators will be provided with the BEBS in form of Excel file or in paper version. In the CRF only summary score will be entered. Each score assessment has to be printed out and filed in the corresponding patient's CRF folder.

- **The Epidermolysis Bullosa Disease Activity and Scarring Index (EBDASI)**, a validated scoring instrument for EB that assesses response to therapy separately from chronic damage, and is therefore suitable for interventional trials. EBDASI has demonstrated significant inter-investigator reliability and validity. It includes an activity and damage component for: 1. skin; 2.

scalp; 3. mucous membranes; 4. nails; 5. other epithelialized surfaces (incl. larynx, esophagus, genitourinary), hands-pseudosyndactyly and skin cancer. For details see Appendix 4.

- The progress of finger contractures and pseudosyndactylies will be evaluated by the validated hand function assessment **score of Colville and Terrill**, which assesses the extent of pseudosyndactylies and provides a rough estimation of the hand function. For details see Appendix 5.

- The Mayo Dysphagia Questionnaire-day 30 (MDQ-30) will be used to assess esophageal involvement. This validated 28-item tool measures esophageal dysphagia within the last 30 days before the visit and will assess improvement of swallowing and eating during treatment. For details see Appendix 6.

7.6.8 Morphometric scoring instrument of pseudosyndactyly progression

A morphometric scoring instrument of pseudosyndactyly progression will be applied (for details on this assessment see Appendix 11). The study staff will take volar, dorsal and lateral photographs of both hands along with a centimeter scale. The length of the lower arm and the body height as internal reference has to be measured at the same clinic visit. Additionally the hand contours will be drawn using a perpendicular pen, and the shadow area will be determined. Details on how to take photographs, draw hand contours, process and send the files/data for central assessment are given in laboratory manual. At the end of the study, the central results will be converged with the clinical database.

7.6.9 Laboratory tests (local assessment)

It is recommended to perform laboratory tests according to clinical praxis (for details see Flowchart) and as clinically indicated. In the CRF only the following parameters will be collected: hemoglobin, NT-proBNP, CRP and ferritin.

7.6.10 Fibrotic and inflammatory markers in serum (central assessment)

A central laboratory will be used for analysis of all specimens collected. Details on all laboratory procedures, collections, shipment of samples and reporting of results, alerting of extreme values and notable values by the central laboratory will be provided to investigators in the laboratory manual. The central laboratory will provide the sponsor with a copy of the laboratory certification and tabulation of the reference ranges, if applicable.
Markers of inflammation and fibrosis in blood which will be assessed centrally comprise: amyloid, TGFβ, IL6, TNFα, PINP and PIIINP. A blood sample for serum analysis will be collected at all clinic visits. The samples have to be processed as described in laboratory manual. For further details please refer also to Appendix 13.
Complete instruction for sample collection, processing, handling and shipment will be provided in the laboratory manual. At the end of the study, the central results will be converged with the clinical database.

7.6.11 Electrocardiogram (ECG) and echocardiography

A 12-lead ECG will be performed at screening and at every subsequent clinic visit according to the flow chart. The electrodes for the ECG should be applied very carefully on the patients' skin, with the adhesive site carefully trimed with scissors. The electrodes can be placed on the limbs and non-adhesive tape can be used to wrap the limbs, fixing the electrodes to the skin. The ECG electrodes will be tucked under Mepilex transfer dressing to be fixed on the chest skin.

An echocardiography to monitor the heart function will also be performed at every clinic visit.

Both the ECG and echocardiography will be performed and evaluated by the pediatric cardiologists that are participating in the trial. Only clinical significant abnormalities will be reported as AE in the CRF.

7.6.12 Skin biopsy (central)

Two optional skin biopsy specimens will be obtained, one at clinic Visit 1 and one at the end of full-dose losartan treatment (Visit 9- clinic, week 40).

A 4-mm punch biopsy will be taken from clinically unaffected skin, 1-2 cm from the border of scarred/fibrotic area at a similar site on the hands or feet, in order to increase reproducibility and to minimize variation arising from acute blistering-associated inflammation. The biopsies will be fixed in formalin immediately after collection and processed for (immuno)histological analysis.

For further details please refer to Appendix 13.

Complete instruction for sample collection, processing, handling and shipment will be provided in the laboratory manual. At the end of the study, the central results will be converged with the clinical database.

7.6.13 Itch, pain, QOL and exacerbating

Specific scores to visualize the patients' burden of itch, pain and quality of life will be filled out at screening and all subsequent clinic visits. To that end the following scoring systems will be used:

- The **Itch Assessment Scale for the Pediatric Burn Patients**, a validated instrument for assessing skin burns (Morris et al., 2012), which are similar to RDEB. The scale is based on the Itch Man Scale developed by Blakeney and Marvin (2000), and will be used to evaluate the intensity of itch of the trial participants. Since itch is also a sign of inflammation, we expect to observe reduction of itch after losartan initiation. For details see Appendix 7.
- The **Wong-Baker FACES Scale for Pain** that uses happy or sad faces shown in the score (http://wongbakerfaces.org/) is the preferred method of pain reporting by children (Wong and Baker, 2001). This validated tool is widely used to rate pain severity in children with both

chronic and acute pain. The scale shows a series of faces ranging from a happy face at 0, "no hurt" to a crying face at 10, "hurts worst". For details see Appendix 8.

- The **Quality Of Life in EB (QOLEB) questionnaire** was developed specifically to measure the quality of life of adults and children with EB and has been shown to be statistically reliable and valid for this group (Frew and Murrell, 2010). Notably, in the published study only children above the age of 11 were able to complete it without parental assistance. Therefore, we will have children under the age of 10 years include their parents' advice when completing the questionnaires, and for children under 8 years of age or unable to read, have the parents complete the questionnaire. For details see Appendix 9.

- The **Children's Dermatology Life Quality Index (CDLQI)** will be used in addition, since it has a version for young children, age of $\geq$ 4, using cartoon images. It consists of 10 questions regarding how the skin disease has affected the patient's quality of life over the past 1 week, in each of 10 domains, with 4 possible responses graded 0–3 (range of scores 0–30). For details see Appendix 10.Checklist of **exacerbating factors** to verify the impairment of every days life of the patients will be performed on a weekly base and queried during phone visits. It consists of 8 questions regarding normal, increased or decreased performance, need to sleep, mobility behavior, anxiety, sadness, weeping, nausea, appetite and other individual factors. For details see Appendix 11.

All these scores will be filled out by the patient him/herself or with help of the parents/ legal guardians in younger children (below that age of 11). It is important that the investigator is not influencing the patient in any way.

7.7 Treatment after end of the trial

After end of the trial, the patients will be treated according to the standards of care. The currently best-available existing treatment for RDEB relies on the symptomatic treatment of blisters, wounds and systemic complications (El Hachem et al., 2014).

In case the patient does have a benefit under IMP administration, further treatment with losartan suspension after end of the trial will be offered to the patient as an off-label use, after thoroughly informing the patient's parents/ legal guardians. The patient's parents/ legal guardians will be trained how to survey the patient's blood pressure and pulse rate. The patient's blood pressure and heart rate would then have to be recorded at least once yearly by his pediatrician.

8 Discontinuation criteria

8.1 Premature termination of the entire trial

The sponsor/coordinating investigator is under obligation to monitor the progress of the clinical trial with regard to safety-relevant developments and, if necessary, initiate the premature termination of the entire clinical trial. The sponsor/coordinating investigator will be supported in this responsibility by the DMC, if necessary.

The entire clinical trial must be terminated prematurely if:

- the benefit-to-risk ratio for the patient changes markedly,
- the sponsor/ coordinating investigator (German LKP) or the DMC considers that the termination of the trial is necessary,
- indications arise that the trial patients' safety is no longer guaranteed,
- the question(s) addressed in the trial can be clearly answered on the basis of an interim analysis,
- the questions(s) addressed in the trial can be clearly answered on the basis of results of another trial on the same subjects,
- an insufficient recruitment rate makes a successful conclusion of the clinical trial appear impossible.

If the clinical trial is prematurely terminated or suspended for any reason, the investigator should promptly inform the trial patients and ensure appropriate therapy and follow-up for the patients. Where required by the applicable regulatory requirements, the competent authority(ies) and the ethic committee(s) will also be informed by the sponsor.

8.2 Premature termination of the trial at one of the trial centres

Both the investigator and the sponsor have the right to terminate the trial at one of the centres.

The clinical trial can be terminated prematurely at the centre by the investigator after consultation with the coordinating investigator if, for instance unforeseeable circumstances have arisen at the trial centre which preclude the continuation of the clinical trial, the investigator considers that the resources for continuation are no longer available, the investigator considers that the continuation of the trial is no longer ethically or medically justifiable.

The Sponsor can initiate the exclusion of a centre from further participation if, for instance, patient recruitment is inadequate, serious problems arise with regard to the quality of the collected data which cannot be resolved.

Premature termination at one of the trial centres does not automatically mean a termination of the trial for already enrolled trial patients. A separate decision on further treatment must be made for each patient, depending on the overall situation. Adequate further treatment and follow-up of already enrolled trial patients must be ensured. The documentation of already enrolled trial patients will be reviewed for completeness and plausibility. Queries may be raised for further clarification before the centre is closed. These queries must be answered properly by the centre. The competent authority(ies) and ethics committee(s) must be duly notified of the centre's closure, including reasons, within the specified period. The trial centre concerned will be closed in stages by the CRA when a decision has been made on the further treatment of the patients concerned.

8.3 Discontinuation criteria for individual trial patients

It has to be distinguished if trial treatment of a patient has been stopped prematurely or if the trial participation of a patient was stopped prematurely.

In case the trial treatment of a patient has been stopped prematurely, further follow-up visits and the assessment of the trial endpoints are essential to enable an analysis of the full analysis set according to the intention-to-treat principle. Further visits, follow-up and documentation should always be striven for/ensured in this case. This includes the follow-up of AEs, the time of termination, the results available at that time and, if known, the documentation of the termination of treatment on the CRF and in the medical record, giving reasons, a final examination and documentation according to the protocol (if possible).

In case the trial participation of a patient was stopped prematurely, the conduct of further follow-up visits is no longer possible.

The documentation should be completed as far as possible under these circumstances, e.g. a final examination and documentation according to the protocol (if possible), a documentation of the premature trial termination on the CRF and in the medical record, giving reasons, appropriate further treatment and follow-up outside the trial should be ensured; inform GP of the termination, if necessary (provided that the patient agrees).

8.3.1 Premature discontinuation of trial treatment

The trial patient can have his/her trial treatment terminated prematurely at any time, without having to give reasons.

The investigator responsible for the trial has the right to terminate the treatment of a patient according to the following conditions:

- Adverse events (including intercurrent illnesses) which preclude further treatment with the IMP or make further participation in the clinical trial inadvisable because the informational value of the trial results is impaired.

- Cardiovascular parameter: if a patient has repeated and clinical relevant arterial hypotension despite sufficient drinking quantity/daily amount of fluid intake. This is the case if there is hypotonic blood pressure (below the 3rd. percentile) in combination with clinical relevant symptoms (for details see 6.2). Before the trial treatment is stopped, a comprehensive investigation (including echocardiography and echocardiogram) in the pediatric cardiology of the trial site shall take place.

- Laboratory values: if liver – or kidney values increase more than threefold compared to the limit value, or if there is an existing electrolyte displacement (potassium <3.0 or > 6.0 mmol/l; sodium <130 or >150mmol/l) despite adjusted fluid balance during repeated controls.

- Premature termination of the trial treatment is considered to be medically indicated, e.g. because it is subsequently found that inclusion/exclusion criteria were violated.

- Continuation of the trial treatment is unacceptable when the risks outweigh the benefits.
- Pregnancy.
- Significant violations of the trial protocol or lack of compliance on the part of the patient (e.g. taking prohibited medication).

8.3.2 Premature termination of trial participation

The trial patient can withdraw his/her consent at any time, without having to give reasons, and have his/her entire trial participation terminated prematurely. However, the prerequisite for this is that the patient actively terminates trial participation by withdrawing his/her consent for the follow-up and documentation.

The responsible investigator may only withdraw a patient from participation in the trial for the following reasons:

- Loss of contact.
- Extreme circumstances arise which make any trial-relevant follow-up impossible.

9 Investigational medicinal product (IMP)

Losartan was developed in the 1990’s by Merck and then sold for the treatment of high blood pressure. It is a synthetic oral selective angiotensin-II receptor (type AT1) antagonist which blocks all physiologically relevant actions of angiotensin II, including vasoconstriction, the release of aldosterone and smooth muscle cell proliferation.

9.1 Pharmacokinetics

Following oral administration, losartan is well absorbed and undergoes first-pass metabolism, forming an active carboxylic acid metabolite and other inactive metabolites. The systemic bioavailability of losartan tablets is approximately 33 %. Mean peak concentrations of losartan and its active metabolite are reached in 1 hour and in 3-4 hours, respectively.

Following oral administration, plasma concentrations of losartan and its active metabolite decline poly-exponentially with a terminal half-life of about 2 hours and 6-9 hours, respectively. During once-daily dosing with 100 mg, neither losartan nor its active metabolite accumulates significantly in plasma.

The pharmacokinetics of losartan has been investigated in 50 hypertensive pediatric patients > 1 month to < 16 years of age following once daily oral administration of approximately 0.54 to 0.77 mg/kg of losartan (mean doses). The results showed that the active metabolite is formed from losartan in all age groups. The results showed roughly similar pharmacokinetic parameters of

losartan following oral administration in infants and toddlers, preschool children, school-age children and adolescents. The pharmacokinetic parameters for the metabolite differed to a greater extent between the age groups. When comparing preschool children with adolescents these differences became statistically significant. Exposure in infants/ toddlers was comparatively high. For further detail please refer to the current SmPC.

9.2 Side effects of IMP

Losartan seems to have the same safety profile in pediatric population as in adults.

The most common adverse reactions are the following: dizziness, low blood pressure, dose-related orthostatic effects, fatigue, headaches, hypoglycaemia, hyperkalaemia, kidney failure, anaemia, and increase in blood urea, serum creatinine and serum potassium in patients with heart failure, muscle cramps, chest pain.

For further details on adverse drug reactions please refer to the current version of the SmPC.

9.3 IMP specifications

In this trial treatment is defined by active substance. Any brand of the IMP in form of tablets can be used. The IMP characteristics given below originate from current SmPC chosen by the sponsor as Reference Safety Information (RSI) for assessment of expectedness of serious adverse reactions (SARs).

The IMP used in this trial is characterized as follows:

Proprietary name:	Losartan HEXAL ®
Name of substance:	**Losartan potassium**
Manufacturer:	Hexal
Approved indications:	- High blood pressure (hypertension) in adults and in children and adolescents 6 – 18 years of age<br>- Kidney protection in hypertensive type 2 diabetes<br>- Chronic heart failure<br>- Reduction of risk of stroke
Dosage form:	Film-coated tablets
Strength:	50 mg
Maximum daily dose:	1.4 mg/kg

The film-coated losartan tablets above will be used for preparation of the losartan suspension (2.5 mg/ml) according to manufacturing procedure of the central pharmacy (see responsibilities) included in a separate document.

9.4 Packaging, labelling, supply and ordering

Losartan suspension will be packaged in amber glass bottles, labeled at the central pharmacy (see responsibilities). Supply and ordering will be described in the IMP handling manual/instruction.

9.5 Receipt and storage

Trial medication will be received by a designated person at the investigational site/ site pharmacy or by a patient's physician/ patient's home pharmacy or by the patient/ patient's parents/ legal guardians and stored safely and properly, and kept in a secured location. Detailed procedure will be described in the IMP handling manual/instruction.

The suspension has to be stored in a refrigerator (at 2°C to 8°C) for up to 4 weeks. The patient/ patient's parents/ legal guardians will be thoroughly instructed about storage conditions.

The investigator will be responsible for ensuring the correct storage and sufficient stocks of the IMP at the site. Where allowed/required, the investigator may/should entrust the IMP, in whole or in part, to an appropriate pharmacist (to be designated in advance) or another appropriate individual who is under the supervision of the investigator.

The investigator or a pharmacist or another appropriate individual who is designated by the investigator, should maintain records of the delivery of the IMP and the stocks at the study site.

9.6 Dispensing

Due to short expiry date (four weeks) the manufactured losartan suspension will be delivered (between clinic visits) to the patent's preferred pharmacy/ patient's physician or to the patient/ patient's parents/ legal guardians. Further details will be described in the IMP handling manual/instruction.

9.7 Return and destruction

The IMP supply can be destroyed at the study site, as appropriate according to SOP and / or local regulations.

9.8 Drug compliance and accountability

Compliance will be assessed by the investigator and/or trial personnel, as at each clinic visit this information will be collected on the appropriate drug accountability forms. This information must be entered in the source document at each patient clinic visit to accurately determine the patient's drug exposure throughout the trial. Trial treatment compliance will be assessed by the investigator or designee at each clinic visit by means of drug accountability.

The investigator or designee must maintain an accurate record of the shipment and dispensing of IMP in a drug accountability log. Drug accountability will be noted by the field monitor during site visits and at the completion of the trial.

Patients will be asked to return all used/ empty/ unsealed/ damaged and/or unused IMP and packaging at every clinic visit, at the end of the trial or at the time of drug discontinuation. Used IMP kits may be destroyed and have not be brought to the clinic visits, the corresponding documentation in the patients diary must be thoroughly done.

The investigator or a pharmacist, or another appropriate individual who is designated by the investigator, should maintain records of the delivery of the IMP, the stocks at the study site, the use by the individual trial patients, and the return of IMP to the sponsor or their disposal. The investigator should ensure that the IMP is only used according to this protocol.

- The investigator bears the responsibility for the proper storage in an appropriate place to which unauthorized persons have no access.

- The investigator may only dispense the IMP to patient's parents/ legal guardians who have been enrolled in the study. The dispensing of the IMP to patients outside of this clinical trial is not permitted.

- The investigator, or an individual who is designated by the investigator, should explain the correct use of the IMP to each trial patient and check at regular intervals that each patient is following the instructions correctly.

- The investigator should take notice of the IMP handling manual/instruction.

9.9 Treatment adherence

Face-to-face adherence reminder sessions (patients/ legal guardians and investigator or another appropriate individual who is designated by the investigator) will take place at the initial product dispensing and each study visit thereafter. This session will include:

- The importance of following study guidelines for adherence to once daily IMP.
- Instructions about taking IMP including timing, storage, and importance of taking suspension, and what to do in the event of a missed dose.
- Notification that there will be a IMP count at every study visit (used/ empty/ unsealed/ damaged and/or unused bottles have to be shown to the investigator at each clinic visit)
- Importance of calling the clinic if experiencing problems possibly related to study product such as symptoms or loss or damage of bottles.
- Completion of patient's diary will be explained.

Subsequent sessions will occur at the follow-up visits. Participants will be asked about any problems they are having taking their study suspension. There will be brief discussion of reasons for missed doses and simple strategies for enhancing adherence, e.g. linking suspension taking

to meals or other daily activities. Participants will have an opportunity to ask questions and key messages from the initial session will be reviewed as needed.

10 Safety monitoring and reporting

10.1 Adverse events (AEs)

10.1.1 Definition of AEs

An adverse event (AE) is any untoward medical occurrence in a patient administered any dose of an IMP and which does not necessarily have to have a causal relationship with the use of the product. An AE can therefore be any unfavourable and unintended sign (including an abnormal laboratory finding), symptom, or disease temporally associated with the use of an IMP, whether or not related to the product.

- In order to monitor the conditions of the patients from the time the patients receive the first dose of IMP the investigator is requested to report any untoward clinical event on the AE-page of the CRF. Any untoward medical occurrence, which occurs after the period of patient follow-up defined in the protocol, is not considered an AE.

- Irrespective of any causal relationship, all AEs spontaneously reported by the patient or observed by the investigator will be continuously documented in the medical record and on the designated case report form (AE CRF page).

- All AEs must be described by diagnosis or, if an underlying diagnosis is not known, by symptoms or medically significant laboratory or instrumental abnormalities. The AEs will be documented as shown in section 10.1.2). Please note that medical or surgical procedures (e.g., tooth extraction, surgery) performed are not AEs *per se*; the medical condition that leads to the procedure is an AE (with the exception of the diagnosis of EB).

- Symptoms, medically significant laboratory, or instrumental (e.g. electrocardiographic) abnormalities of a pre-existing disease are not to be considered an AE. Occurrences of new symptoms or laboratory or instrumental abnormalities, as well as worsening of pre-existing ones, are considered AEs.

- All AEs, no matter how intense, are to be followed up by the investigator in accordance with ICH-GCP until resolved or judged no longer clinically relevant, or in case of a chronic condition, until it is fully characterized.

- Overdose without clinical sequelae, is not to be considered an AE.

- As in this trial the participants suffer from RDEB, which is associated with significant multi-organ involvement and often necessitates routine multidisciplinary care, AEs judged by the investigator to belong to the disease course (e.g. development of infected wounds) do not have to be documented in the CRF.

10.1.2 Documentation of AEs

Adverse events have to be documented in the CRF starting from the first administration and until 30 days after the last administration of IMP.

- Characterization of the event
- Onset
- End
- Severity according to the current version of CTCAE
- Relationship to the IMP

  Note:

  According to the CIOMS VI Working group the causal relationship between the investigational product and the adverse event should be characterized as "related" or "not related" (the various gradients of relatedness offer little or no advantages in data analysis or regulatory reporting).
- Serious or non-serious
- Action taken with IMP
- Outcome

10.2 Serious Adverse Events (SAEs)

10.2.1 Definition of SAEs

A Serious Adverse Event (SAE) is any untoward medical occurrence that results in any of the following outcomes:

- Death,
- Life-threatening situation (patient is at <u>immediate</u> risk of death),
- Inpatient hospitalization or prolongation of existing hospitalization (excluding those for study therapy and/or assessments, routine treatment of RDEB or additional diagnostics procedures, placement of an indwelling catheter, elective or pre-planned treatment/surgery, social/convenience admissions, and respite care)
- Persistent or significant disability/incapacity,
- Congenital anomaly/birth defect,
- Other, <u>medically important condition</u>: conditions which, in the investigator's opinion, may not be immediately life-threatening or result in hospitalization, but may jeopardize the patient's safety or may require intervention to prevent one of the other outcomes listed in the definition above, may also be considered serious. Examples of such conditions include: allergic bronchospasm requiring treatment in an emergency room or at home, unexpected convulsions (i.e. convulsions which cannot be explained by the underlying illness) that do not result in hospitalization, development of IMP dependency or drug abuse, suspected transmission of infectious agents by medicinal product, etc.

<u>Clarification of SAEs:</u>

- NOTE: The term “life-threatening” in the definition of “serious” refers to an event/reaction in which the patient was at risk of death at the time of the event/reaction; it does not refer to an event/ reaction which hypothetically might have caused death if it were more severe.
- As in this trial the participants suffer from RDEB, which is associated with significant multi-organ involvement and often necessitates in-hospital stay for multidisciplinary care, hospitalization *per se* will not be considered as criterion for expedited reporting. This excludes hospitalization or prolongation of hospitalization judged by the investigator to be unanticipated with regard to disease course.

10.2.2 Documentation of SAEs

All SAEs that occur starting from the first administration and until 30 days after the last administration of the IMP will be documented in the CRF and on the provided SAE reporting form.

The SAE reporting form will be processed as described in the section below.

10.2.3 Investigator reporting requirements

10.2.3.1 SAE reporting policy

All SAEs must be reported by fax to the following address within 24 hours after knowledge by the investigator:

Pharmacovigilance
Clinical Trials Unit
Medical Center - University of Freiburg
Elsaesser Str. 2, 79110 Freiburg

SAE Fax No.

+49 761 270-74 390

If only limited data are initially available, a follow-up report is required. If new information including outcome becomes available or relationship to IMP is reconsidered, a SAE follow-up report should be sent within 24 hours using the same procedure as for transmitting the initial SAE report (details will be provided in SAE reporting manual).

10.2.3.2 Reporting of patient death

Please note that “death” is usually an SAE outcome and not an SAE *per se*. Only in cases where the clinical circumstances before the death are unknown (i.e. patient died without a determinable cause of death), then the diagnosis “death” itself should be reported as an SAE. In case of fatal outcome of an already-registered SAE, a follow-up notification must be done.

If a patient dies, the CRF page designated for death documentation must be faxed to the Pharmacovigilance CTU within 3 working days after knowledge.

According to section 12, subsection 6 GCP-V, in case of patient’s death the investigator must submit on demand all information to the competent IEC, the other IEC(s) involved, the CA and the sponsor, that is required for the fulfilment of their duties (note that personal data must be transmitted using the trial-specific patient identification number, i.e. in pseudonymised form).

10.2.3.3 Reporting of premature treatment/study discontinuation

The investigator must fax the CRF page designated for premature treatment/study discontinuation to the Pharmacovigilance CTU within 3 working days after knowledge to enable the sponsor to survey continuously safety of study participants and to fulfil legal reporting requirements.

10.2.4 Sponsor reporting requirements

The sponsor's reporting requirements are divided into expedited reporting and reporting that must be performed on request or annually.

10.2.4.1 Definition of SUSARs

The sponsor's expedited reporting requirements are particularly relevant to suspected unexpected serious adverse reactions (SUSARs). The definition is a combination of the definitions of serious adverse reaction (for seriousness criteria see section 10.2.1) and unexpected adverse reaction (adverse reaction: the nature or severity of which is not consistent with the applicable RSI (SmPC and/or IB) for the IMP).

10.2.4.2 SUSAR/ circumstance requiring a review of the benefit/risk evaluation

The sponsor's expedited reporting requirements comprise the following:

- All SUSARs must be reported within 15 days after knowledge (section 13, subsection 2 GCP-V),
- All SUSARs that are life-threatening or result in death must be reported within 7 days after knowledge (section 13, subsection 3 GCP-V),
- All circumstances requiring a review of the benefit/risk evaluation of the IMP must be reported within 15 days after knowledge (e.g. expected serious adverse reaction with unexpected outcome, increased incidence of expected serious adverse reactions, SUSARs after the end of the patient's participation in the clinical trial, events in connection with the trial conduct or the development of the IMP which may affect the safety of the trial patients) (section 13, subsection 4 GCP-V).

10.2.4.3 Development Safety Update Report (DSUR)

In addition to the expedited reporting, the sponsor shall submit an annual report once a year or on request throughout the clinical trial period, according to section 13, subsection 6 GCP-V and ICH

guideline E2F. The aim of the DSUR is to concisely describe all new safety information relevant for one or several clinical trial(s), to assess the safety conditions of subjects included in the concerned trial(s) and to evaluate whether the benefit / risk ratio is still favourable.

10.2.5 Pregnancies

Any pregnancy (female trial participant or female partner of male trial participant) that occurs during trial participation must be reported. To ensure patient safety each pregnancy must be reported to Pharmacovigilance CTU on the pregnancy reporting form within 24 hours of learning of its occurrence. The pregnancy should be followed up to determine outcome, including spontaneous or voluntary termination, details of birth, and the presence/absence of any birth defects, congenital abnormalities or maternal and new-born complications.

11 Data handling and data management

11.1 Data confidentiality

Information about trial patients will be kept confidential and managed under the applicable laws and regulations. Those regulations require a signed patient authorization informing the patient of the following:

- what protected health information (PHI) will be collected from patients in this trial;
- who will have access to that information and why;
- who will use or disclose that information;
- the rights of a research patient to revoke their authorization for use of their PHI.

In the event that a patient revokes authorization to collect or use PHI, the investigator, by regulation, retains the ability to use all information collected prior to the revocation of patient authorization. For patients that have revoked authorization to collect or use PHI, attempts should be made to obtain permission to collect at least vital status (i.e. that the patient is alive) at the end of their scheduled trial phase.

The data collection system for this trial uses built-in security features to prevent unauthorized access to confidential participant information. Access to the system will be controlled by individually assigned user identification codes and passwords, made available only to authorized personnel who have completed prerequisite training.

11.2 Documentation of trial data

11.2.1 Documentation in medical records

The investigator will record the participation in the trial, informed consent procedure, frequency of the trial visits, relevant medical data, concomitant treatment and the occurrence of adverse events in the medical record/patient’ chart of each trial patient.

11.2.2 Documentation in CRF

The investigator, or a deputy who is designated by the investigator, will document the trial data on a trial-specific case report form (CRF) as promptly as possible. The following CRF data are stated to be the source data in this study:

- severity of AEs and
- relationship of AEs to the IMP.

These data will be directly reported on the CRF pages.

Hard copy CRFs will be used in this trial. The hard copy CRFs consist of 2-layer Non-Carbon-Required paper (NCR paper). Corrections and subsequent changes to CRF pages must be made according to the ICH-GCP guidelines provided in the CRF completion instructions at the beginning of each CRF-folder.

Any queries on the part of Data Management staff will be sent to the site on special forms for subsequent correction (via the CRA, if necessary). The query forms should be completed close to the times of the planned monitoring visits or at the times specified by the trial coordinator. The query forms which contain the corrections must be confirmed by the dated signature of the investigator (not the Study Nurse) in the designated places. They will be collected by the CRA at the visits or sent directly to the Data Management.

11.3 Data management

The data management will be performed with DAMAST Version 03-02, a proprietary data management system based on the software package SAS, which is developed, validated and maintained by the CTU.

Details on data management (procedures, responsibilities, data corrections, if any, which may be made by Data Management, staff themselves, etc.) will be described in a data management manual prior to the trial. The Data Management Manual is a working document which will be continuously updated and maintained during the trial, i.e. the performance of data management and any deviations from the first version of the data management manual will be documented therein, as well.

Before any data entry is performed, the trial database will be validated and the technical specifications of the database will be documented. Double data entry will be performed by two different persons (with the exception of free text). The comparison of both entries and the resolution of discrepancies is only performed by trained staff. An audit trail will be created to provide an electronic record of which data were entered or subsequently changed, by whom and when.

SAS software will be used to review the data for completeness, consistency and plausibility, and regarding protocol violations and other distinctive problems (e.g. cumulative missings). The checks to be programmed will be specified beforehand in a data validation plan. After running the check programs, the resulting queries will be sent to the investigator for correction or verification of his/her data. Answered queries will also be entered twice, verified and the updated data will then be transferred to the database. All programs which can be used to influence the data or the

data quality will be validated (e.g. check programs, programs used for the import of external data, etc.).

11.4 Data coding

Concomitant medication will be coded using the WHO Drug dictionary. Adverse events will be coded using the Medical dictionary for regulatory activities (MedDRA) terminology.

12 Quality assurance

The sponsor is responsible for implementing and maintaining quality assurance and quality control systems with written SOPs to ensure that trials are conducted and data are generated, documented (recorded), and reported in compliance with the protocol, GCP, and the applicable regulatory requirement(s).

12.1 Monitoring procedure

The monitoring is performed by the CRAs of the CTU. Risk-based monitoring will be done according to ICH-GCP E6 and standard operating procedures (SOP) to verify that patients' rights and wellbeing are protected, reported trial data are accurate, complete and verifiable from source documents and that the trial is conducted in compliance with the currently approved protocol/amendment, with GCP and with the applicable regulatory requirements to ensure safety and integrity of clinical trial data.

The investigator will accept monitoring visits before, during and after the clinical trial. Prior to the trial, a site initiation visit at each site is conducted in order to train and introduce the investigators and their staff to the trial protocol, essential documents, handling of IMP and related trial specific procedures, ICH-GCP and national/local regulatory requirements.

During the trial, the monitor will visit the site regularly depending on the recruitment rate and quality of data. During these on-site visits, the monitor verifies that the trial is conducted according to the trial protocol, trial specific procedures, ICH-GCP and national/local regulatory requirements. The presence of signed ICFs, eligibility of patients, primary endpoint, handling of IMP and documentation/reporting of safety data (e.g. AE/SAE) will be verified by the monitor. The monitor is also performing source data verification to ensure clinical trial data are recorded and documented in the source data and CRFs are complete and accurate. Extent of source data verification and monitor visit frequency will be adapted for individual sites in case of lack of data quality or a high number of protocol violations. All trial specific monitoring procedures, monitoring visit frequency and extent of SDV will be predefined in a trial specific monitoring manual. The investigator must maintain source documents for each patient in the trial, consisting of case and visit notes (hospital or clinic medical records) containing demographic and medical information, laboratory data, electrocardiograms, and the results of any other tests or assessments (see section 7). All information recorded on CRFs must be traceable to source documents in the patient's file. The investigator must also keep the original signed ICF (a signed copy is given to the patient).

The investigator must give the monitor access to all relevant source documents to confirm their consistency with the CRF entries.

12.2 Source data verification (SDV)

Source data as defined by ICH-GCP include original documents, data, and records such as hospital records, clinical and office charts, laboratory notes, memoranda, patients' diaries or evaluation checklists, pharmacy dispensing records, recorded data from automated instruments, copies or transcriptions certified after verification as being accurate copies, microfiches, photographic negatives, microfilm or magnetic media, X-rays, and records kept at the pharmacy, at the laboratories and at medico-technical departments involved in the clinical trial.

Source data verification will be performed in order to verify the accuracy and completeness of the entries on the case report form (CRF) by comparing them with the source data, and to ensure and increase the quality of the data. All data which are subject to SDV must have been entered in the medical record or, in the case of source documents, enclosed with the medical record. The investigators will afford the CRA access to the medical records for the performance of SDV. Extent of Source Data verification will be specified in the in the monitoring manual.

12.3 Auditing procedures and inspections

According to the ICH-GCP guidelines, audits will be performed in frame of a quality assurance system. Audits and/or inspections may be conducted by the sponsor, authority(is) or an independent external party.

The purpose of a sponsor's audit, which is independent of and separate from routine monitoring or quality control functions, should be to evaluate trial conduct and compliance with the protocol, SOPs, GCP, and the applicable regulatory requirements. All persons who conduct an audit undertake in writing to treat all data related to medical secrecy or which could reveal the patient's identity in absolute confidence, and to restrict the use of such data to the purposes agreed by the patient in writing.

Proposed dates for sponsor's audit, characteristics of the selected patients and further information will be transmitted to the investigator by the CRA in a timely manner.

The investigator will inform the CTU immediately of an inspection requested by a regulatory authority. The investigator is responsible for availability of source data/documents to audit/inspections.

13 Biostatistical planning and analysis

Before the start of the final analysis a statistical analysis plan (SAP) will be prepared. This will be completed during the 'blind review' of the data, at the latest. This blind review, i.e. a checking and assessment of the data, will be performed after the end of the recruitment period and the planned follow-up period. If the SAP contains any changes to the analyses outlined in the trial protocol, they will be marked as such, and reasons for amendments will be given.

All statistical programming for analysis will be performed with the Statistical Analysis System (SAS).

13.1 Trial design

For details on trial design see section 3.1 of the protocol.

13.2 Objectives and endpoints

For details on endpoints see section 2 of the protocol.

13.3 Sample size calculation

Sample size calculation is based on the primary endpoint occurrence of severe complication leading to a serious safety concern (see section 2). A total of 30 trial participants is necessary to achieve sufficient power for an assessment of the primary endpoint. If none of the 30 participants develops a severe complication leading to a serious safety concern, it can be concluded that, with a probability of 95%, the severe complication rate is below 10%.

13.4 Definition of populations included in the analyses

All analyses (safety and efficacy) will be performed in the safety population. Patients are included in the safety population, if they received at least one dose of trial medication with losartan.

The per-protocol (PP) population is a subset of the safety population and is defined as the group of patients who had no major protocol violations, received a predefined minimum dose of the trial medication and underwent the examinations required for the assessment of the efficacy endpoints at relevant, predefined times. Efficacy analyses will additionally performed in the PP population for the purpose of a sensitivity analysis.

13.5 Methods of analysis

13.5.1 Patient demographics/other baseline characteristics

Demographic and other baseline data (including disease characteristics) will be summarized descriptively in the safety population.

Continuous data will be summarized by arithmetic mean, standard deviation, minimum, 25% quantile, median, 75% quantile, maximum, and the number of complete and missing observations. If appropriate, continuous variables can also be presented in categories.

Categorical data will be summarized by the total number of patients in each category and the number of missing values. Relative frequencies are displayed as valid % (number of patients divided by the number of patients with non-missing values).

13.5.2 Treatments (trial treatment and compliance, concomitant medication)

13.5.2.1 Trial medication

Duration of trial treatment exposure, cumulative dose and dose intensity will be summarized. The number of patients with dose changes/interruptions will be presented.

13.5.2.2 Concomitant medication

The concomitant medications will be summarized by ATC level 1/3/5. In each table, patients will be counted once, if they took at least one medication from the respective ATC level. The number of patients and the percentage of the total number of patients in the safety population will be given.

13.5.3 Primary endpoint (safety)

All severe complication leading to a serious safety concern will be listed by patient with the given dose of trial medication. The incidence of these severe complications will be calculated as the number of patients who experienced at least one severe complication in percentage of the total number of patients in the safety population. Additionally, the 90%- and the 95%-confidence intervals of the incidence will be calculated. If in none of 30 patients a severe complication leading to a serious safety concern occurs, it can be concluded that, with a probability of 95%, the severe complication rate is below 10%.

13.5.4 Secondary endpoints for efficacy

All patients will receive losartan; the efficacy (secondary endpoints) will be assessed in comparison to their own baseline values before start of therapy. Baseline can be regarded as adequate control at this stage, since a spontaneous improvement of inflammation, fibrosis and mitten deformities cannot be expected and therefore an improvement can be regarded as success of the treatment.

The efficacy endpoints will be analyzed by comparing post-treatment measurements after 8 weeks and after 9 months with pre-treatment measurements and by calculating 95% confidence intervals for the differences. The following statistical tests comparing within patient measurements will be performed: For continuous data for which a normal distribution can be assumed paired t-tests will be used; for continuous data for which a normal distribution cannot be assumed paired Wilcoxon tests will be used; and for binary data the McNemar test tests will be used. Statistical tests will be performed at a significance level of 0.05, but their results will be interpreted in a descriptive sense.

13.5.5 Secondary endpoints for safety

13.5.5.1 Adverse events

All adverse events (AEs) will be listed by center and patient and displayed in summary tables.

The incidence of AEs defined by preferred term (PT) according to MedDRA will be calculated as the number of patients who experienced at least one AE with the respective PT in percentage of the total number of patients in the safety population. In the incidence tables the PTs will be grouped by system organ class (SOC) according to MedDRA. Additionally, the incidence of AEs defined by SOC will be calculated as the number of patients who experienced at least one AE in the respective SOC as percentage of the total number of patients in the safety population.

Each table will be produced for the following AE-sets:

- all AEs
- AEs being a severe complication leading to a serious safety concern
- AEs being at least severe
- Serious Adverse Events (SAEs)
- AEs possibly related to trial medication
- AEs possibly related to trial medication being at least severe (toxicity)
- SAEs possibly related to trial medication

Incidences of AEs will be calculated with 95%-confidence intervals.

13.5.5.2 Laboratory data

Laboratory data will be presented in the measured units (or in SI units, being converted from the original units, if necessary). Values outside the investigator's reference range and being clinically relevant will be flagged as above or below the reference range in the listings. Shift tables for all parameters will also be generated.

For a quantitative summary of laboratory data from laboratories with different reference ranges, the values will be standardized appropriately (Chuang-Stein, 1992).

13.6 Interim analysis

No interim analysis will be performed.

14 Data safety monitoring committee (DSMC)

An independent DSMC consisting of three experts: one in dermatology with specialization in EB, one in pediatric cardiology and one statistician, will be established. The members of the DMC are listed in section "Responsibilities".

The function of the DSMC is to monitor the course of the trial, assess change in risk-benefit ratio and if necessary to give a recommendation to the sponsor/coordinating investigator for discontinuation, modification or continuation of the trial. The underlying principles for the DSMC are ethical and safety aspects for the patients. It is the task of the DSMC to examine, whether the conduct of the trial is still ethically justifiable, whether security of the patients is ensured, and whether the process of the trial is acceptable.

The CTU will provide quarterly the DSMC with the cumulative listings including all SAEs, and premature termination of the study and once a year with DSURs.

The composition and responsibilities of the DSMC, the structure and procedures of its meetings, and its relationship to other key trial team members, will be laid down in a DSMC charter.

The DSMC will hold a phone safety meeting after the 15th enrolled patient has been examined in Visit 3-clinic for reviewing safety data and assess change in risk-benefit ratio.

15 Ethical and legal principles

15.1 Regulatory and ethical compliance

This clinical trial was designed, shall be implemented and reported in accordance with the ICH-GCP, with applicable local regulations (including European Directive 2001/20/EC), and with the ethical principles laid down in the Declaration of Helsinki.

15.2 Responsibilities of the investigator and IEC

The protocol and the proposed ICF must be reviewed and approved by a properly constituted Independent Ethics Committee (IEC) before trial start. A signed and dated statement that the protocol and ICF have been approved by the IEC must be given to sponsor before initiation of the trial. Prior to start of the trial, the investigator is required to sign a protocol signature page confirming his/her agreement to conduct the trial in accordance with these documents and all of the instructions and procedures found in this protocol and to give access to all relevant data and records to sponsor monitors, auditors, sponsor Clinical Quality Assurance representatives, designated agents of sponsor, IECs and regulatory authorities as required.

15.3 Informed consent procedure

Before enrolment in the clinical trial, the patient/ patient’s parents/ legal guardians will be informed that participation in the clinical trial is voluntary and that patient may withdraw from the clinical trial at any time without having to give reasons and without penalty or loss of benefits to which the patient is otherwise entitled.

In pre-screening phase the patients/ patient’s parents/ legal guardians will receive in advance by post an ICF in order to have time to consider participation in the study. The ICF signed by patients/ patient’s parents/ legal guardians as applicable has to be available at screening visit.

The treating physician will provide the patient/ patient's parents/ legal guardians with information about the treatment methods and the possible risks involved. At the same time, the nature, significance, implications, expected benefits and potential risks of the clinical trial and alternative treatments will be explained to the patient/ patient's parents/ legal guardians. During the informed consent discussion, the patient/ patient's parents/ legal guardians will also be informed about the insurance cover that exists and the insured's obligations. The patient/ patient's parents/ legal guardians will be given ample time and opportunity to obtain answers to any open questions. All questions relating to the clinical trial should be answered to the satisfaction of the patient/ patient's parents/ legal guardians. In addition, the patient/ patient's parents/ legal guardians will be given a patient information sheet which contains all the important information in writing.

The patient's written consent must be obtained before any trial-specific tests/treatments.

For this purpose, the written ICF will be personally dated and signed by the trial patient and the investigator conducting the informed consent discussion. *

By signing the ICF, the patient/ patient's parents/ legal guardians agree to voluntarily participate in the clinical trial and declare their intention to comply with the requirements of the clinical trial and the investigator's instructions during the clinical trial. By signing the ICF, the patient/ patient's parents/ legal guardians also declare that they agree to the recording of personal data, particularly medical data, for the trial, to their storage and codified ("pseudonymised") transmission to the sponsor, to the competent authorities, and further agree that authorised representatives of the sponsor Medical Center - University of Freiburg, who are bound to confidentiality, and national or foreign competent authorities may inspect the patient's personal data, particularly medical data, which are held by the investigator.

Signature of the both patient's parents/ legal guardians on the ICF is necessary. In case of absence of one of the parents/ legal representatives at screening visit, her/his signature has to be obtained previously and be available at the time of visit in original or as a fax copy (the original in this case must be provided as soon as possible).

After signing, the patient/ patient's parents/ legal guardians will be given one copy of the signed and dated written ICF and any other written information designated for the patient. The investigator has document the informed consent procedure in the patient's chart.

In case of substantial amendments, the patient/ patient's parents/ legal guardians must be informed with an appropriate revised patient information/consent form. Changed trial procedures can only be carried out if they have been approved by the competent authority and the leading Ethics Committee, and if the patient has been appropriately informed and has given his/her written consent.

** ICH-GCP 4.8.9: If a patient is unable to read or if a legal representative is unable to read, an impartial witness should be present during the entire informed consent discussion. After the written informed consent form and any other written information to be provided to patients is read and explained to the patient or the patient's legal representative, and after the patient or the patient's legal representative has orally consented to the patient's participation in the trial and, if capable of doing so, has signed and personally dated the informed consent form, the witness should sign and personally date the consent form. By signing the consent form, the witness attests that the information in the consent form and any other written information*

was accurately explained to, and apparently understood by, the patient or the patient's legal representative, and that informed consent was freely given by the patient or the patient's legal representative.

Fertile men and women of child bearing potential should be informed that taking the IMP may involve unknown risks to the foetus if pregnancy were to occur during the trial and agree that in order to participate in the trial they must adhere to the contraception requirement for the duration of the trial. If there is any question that the patient will not reliably comply, they should not be entered in the trial.

15.4 Patient insurance

According to article 40, section 1, subsection 8 and article 40 section 3 of AMG, subject insurance (minimum: € 500,000 per subject) has been taken out with

Policy-No. 57 010313 03014<br>HDI Global SE<br>Am Schönenkamp 45<br>40599 Düsseldorf

for all subjects participating in the clinical trial.

The investigator or designee will inform the subject of the existence of the insurance, including the obligations arising from it. The trial subjects must be afforded access to insurance documents and provided with a copy of the general conditions of insurance on request.

15.5 Confidentiality of trial documents and patient records

The investigator must ensure anonymity of the patients; patients must not be identified by names in any documents submitted to sponsor. Signed ICFs and patient identification log must be kept strictly confidential to enable patient identification at the site.

15.6 Financial disclosure

Financial disclosures should be provided by trial personnel who is directly involved in the treatment or evaluation of patients at the site - prior to trial start.

16 Trial documents and archiving

16.1 Trial documents/investigator site file

The investigator will be given an investigator site file containing all the necessary essential trial documents for the initiation of the trial at his/her site. The essential documents include a list on which the investigator will enter all appropriately qualified persons to whom he/she has delegated important trial-related tasks.

The investigator, or an individual who is designated by the investigator, will be responsible for the maintenance and completeness of the trial documents during the clinical trial. At the request of the CRA, auditor, ethics committee or competent authorities, the investigator shall make available all the requested trial-related records for direct access. Essential documents must not be removed permanently.

16.2 Archiving

After completion of the clinical trial, the essential trial documents - as defined by ICH-GCP E6 section 8 - from clinical trials will be retained at the trial site for a sufficient period so that they will be available for audits and inspections by the authorities.

The investigator will be responsible for the storage. The following retention periods will apply after the completion/termination of the clinical trial:

- The above-mentioned essential documents must be retained for at least 10 years (section 13, subsection 10 GCP-V); the identification codes of studies submitted for IMP approval must be retained for at least 15 years (2001/83/EC).
- The medical records and other source documents must be retained for the longest possible period allowed by the hospital, the institution or the private practice.

The investigator/the institution should take measures to prevent accidental or premature destruction of these documents. The sponsor will notify the investigator in writing when the trial-related essential documents are no longer required

17 Protocol adherence and amendments

17.1 Protocol adherence

Investigators ascertain they will apply due diligence to avoid protocol deviations. Under no circumstances should the investigator contact sponsor or its agents, if any, monitoring the trial to request approval of a protocol deviation, as no authorized deviations are permitted. If the investigator feels a protocol deviation would improve the conduct of the trial this must be considered a protocol amendment, and unless such an amendment is agreed upon by sponsor and approved by the IEC it cannot be implemented.

17.2 Amendments to the protocol

Any change or addition to the protocol can only be made in a written protocol amendment that must be approved by sponsor, CA where required, and the IEC.

Only changes of the protocol that are required for patient safety may be implemented prior to IEC approval. Regardless the need for approval of formal protocol amendments, the investigator is expected to take any immediate action required for the safety of any patient included in this trial,

even if this action represents a deviation from the protocol. In such cases, the sponsor should be notified as soon as possible of this action; the IEC should be informed correspondingly.

17.3 Protocol deviations

Protocol deviations will be described in the SAP. Handling with protocol deviations will be described in the monitoring manual.

18 Administrative Agreements

18.1 Financing of the trial and role of the funder

The clinical trial has been financed by DEBRA International / Registered Charity No: ZVR 932762489.

This funding source had no role in the design of this study and will not have any role during its execution, analyses, interpretation of the data, or decision to submit results.

18.1.1 Trial agreement/investigator compensation

According to ICH-GCP 4.9.6, a trial agreement on the conduct of the clinical trial and the compensation for conducting the trial will be signed between the sponsor of the clinical trial and the investigators including their heads of administration.

18.1.2 Reimbursement of trial patients

Reimbursement of travel and accommodation costs for patients/ parents/ legal guardians, as applicable, is foreseen in this study.

18.2 Trial reports

After completion of the analysis by the responsible biostatistician, the coordinating investigator will prepare and sign the final integrated medical and statistical report jointly with the biostatistician.

Except when required by law, no one will disclose a result of the clinical trial to third parties unless all parties involved have first agreed on the results of the analysis and their interpretation.

The final trial report will be written and signed in co-operation between the coordinating investigator and the CTU of Medical Center - University of Freiburg.

18.3 Clinical trials registry

The sponsor ensures that the key design elements of this protocol will be posted in the publicly accessible clinical trials registry: DRKS registry.

18.4 Publication of trial protocol and results

The sponsor assures that the key design elements of this protocol will be posted in a publicly accessible clinical trials registry (see section above). In addition, upon trial completion the results of this trial will be submitted for publication and/or posted in a publicly accessible database of clinical trial results.

Reporting guidelines will be taken into account (see www.equator-network.org), e.g. the CONSORT statement should be adhered to in the preparation of papers on the results of randomized studies.

Each publication of trial results will be in mutual agreement between the coordinating investigator, the other investigators involved and the CTU. All data collected in connection with the clinical trial will be treated in confidence by the coordinating investigator and all others involved in the trial, until publication. Interim data and final results may only be published (orally or in writing) with the agreement of the coordinating investigator and the CTU. This is indispensable for a full exchange of information between the above-named parties, which will ensure that the opinions of all parties involved have been heard before publication. The agreement, which does not include any veto right or right of censorship for any of the parties involved, may not be refused without good reason.

19 References

- Blakeney & Marvin. Copyrighted by Shriners Hospitals for Children. 2000
- Boot-Handford RP, Briggs MD. The unfolded protein response and its relevance to connective tissue diseases. *Cell Tissue Res* 339:197-211, 2010.
- Chuang-Stein C. Summarizing laboratory data with different reference ranges in multi-center clinical trials. *Drug Information Journal*.;26(1):77–84; 1992
- Cook JR, et al. Abnormal muscle mechanosignaling triggers cardiomyopathy in mice with Marfan syndrome. *J Clin Invest* 124:1329-39, 2014.
- Dietz HC. One integrin to rule them all? *Sci Transl Med* 7:288fs21, 2015.
- El Hachem M, Zambruno G, Bourdon-Lanoy E, Ciasulli A, Buisson C, Hadj-Rabia S, et al. Multicentre consensus recommendations for skin care in inherited epidermolysis bullosa. *Orphanet J Rare Dis*.;9:76, 2014
- Fine JD, Mellerio JE. Extracutaneous manifestations and complications of inherited epidermolysis bullosa: part II. Other organs. *J Am Acad Dermatol* 61:387-402, 2009.
- Frew JW, Murrell DF. Quality of life measurements in epidermolysis bullosa: tools for clinical research and patient care. *Dermatol Clin.* ;28(1):185–90; 2010
- Fritsch A, Loeckermann S, Kern JS, Braun A, Bösl MR, Bley T, Schumann H, von Elverfeldt D, Paul D, Erlacher M, Berens von Rautenfeld D, Hausser I, Fässler R, Bruckner-Tuderman L. A hypomorphic mouse model for dystrophic epidermolysis bullosa reveals disease mechanisms and responds to fibroblast therapy. *J Clin Invest* 118:1669-1679, 2008.
- Habashi JP, et al. Angiotensin II type 2 receptor signaling attenuates aortic aneurysm in mice through ERK antagonism. *Science* 332:361-5, 2011.
- Has C, Bruckner-Tuderman L. The genetics of skin fragility. *Annu Rev Genomics Hum Genet* 15:245-68, 2014.
- Intong LR, Murrell DF. Inherited epidermolysis bullosa: new diagnostic criteria and classification.*Clin Dermatol* 30:707, 2012.
- Kern JS, Loeckermann S, Fritsch A, Hausser I, Müller ML, Paul O, Ruther P, Bruckner-Tuderman L: Mechanisms of fibroblast cell therapy for dystrophic epidermolysis bullosa: high stability of collagen VII favors long-term skin integrity. *Mol Ther.* 17, 1605-15, 2009
- Klett CC Hereditary disorders of connective tissue: a review. *Wound Repair and Regeneration* 5:3-11, 1997.
- Kühl T, Mezger M, Hausser I, Handgretinger R, Bruckner-Tuderman L, Nyström A: High Local Concentrations of Intradermal MSCs Restore Skin Integrity and Facilitate Wound Healing in Dystrophic Epidermolysis Bullosa. *Mol Ther* 23:1368-1379, 2015.
- Küttner V, Mack C, Gretzmeier C, Bruckner -Tuderman L, Dengjel J: Loss of collagen VII is associated with reduced transglutaminase 2 abundance and activity. *J Invest Dermatol* 134:2381-9, 2014.
- Küttner V, Mack C, Rigbolt KTG, Kern JS, Schilling O, Busch H, Bruckner-Tuderman L, Dengjel J. Global remodeling of cellular microenvironment due to loss of collagen VII. *Mol Syst Biol 16,9:657;2013*
- Lacro RV, et al. Atenolol versus losartan in children and young adults with Marfan's syndrome. *N Engl J Med* 371:2061-71, 2014.
- Laimer M, Bauer JW, Hintner H. Dystrophic Epidermolysis bullosa. In: Murrell DF (Ed). Blistering Diseases- Clinical Features, Pathogenesis, Treatment. Springer Berlin Heidelberg New York Dondrecht London, pp. 419-30, 2015b,
- Laimer M, Prodinger C, Bauer JW. Hereditary epidermolysis bullosa. JDDG: *Journal der Deutschen Dermatologischen Gesellschaft.* ;13(11):1125–33,2015

- Mittapalli VR, Madl J, Löffek S, Kiritsi D, Kern JS, Römer W, Nyström A, Bruckner-Tuderman L. Injury-Driven Stiffening of the Dermis Expedites Skin Carcinoma Progression. *Cancer Res*. 2015
- Morris V, Murphy LM, Rosenberg M, Rosenberg L, Holzer CE 3rd, Meyer WJ 3rd. *J Burn Care Res.* 33(3):419-24, 2012
- Neill T, Schaefer L, Iozzo RV. Instructive roles of extracellular matrix on autophagy. *Am J Pathol* 184:2146-53, 2014.
- Nischler E, Klausegger A, Hüttner C, Pohla-Gubo G, Diem A, Bauer JW, et al. Diagnostic Pitfalls in Newborns and Babies with Blisters and Erosions. *Dermatology Research and Practice*.;320403, 2009
- Nyström A, Thriene K, Mittapalli K, Kern JS, Kiritsi D, Dengjel J, Bruckner-Tuderman L. Losartan ameliorates dystrophic epidermolysis bullosa and uncovers new disease mechanisms. *EMBO Mol Med.* 7:1211-28, 2015.
- Nyström A, Velati D, Mittapalli VR, Fritsch A, Kern JS, Bruckner-Tuderman L. Collagen VII plays a dual role in wound healing. *J Clin Invest* 123:3498-509, 2013.
- Odorisio T, et al. Monozygotic twins discordant for recessive dystrophic epidermolysis bullosa phenotype highlight the role of TGF-β signalling in modifying disease severity. *Hum Mol Genet* 23:3907-22, 2014.
- Pees C, Laccone F, Hagl M, Debrauwer V, Moser E, Michel-Behnke I. Usefulness of losartan on the size of the ascending aorta in an unselected cohort of children, adolescents, and young adults with Marfan syndrome. *Am J Cardiol.* 112(9):1477-83, 2013
- Ramirez F, Rifkin DB. Is losartan the drug for all seasons? *Curr Opin Pharmacol* 12:223-4, 2012.
- Terrill PJ, Mayou BJ, Pemberton J. Experience in the surgical management of the hand in dystrophic epidermolysis bullosa. Br *J Plast Surg* 45: 435–442, 1992
- Varki R, Sadowski S, Uitto J, Pfendner E.Epidermolysis bullosa. II. Type VII collagen mutations and phenotype-genotype correlations in the dystrophic subtypes. *J Med Genet* 44:181-92, 2007.
- Wally V, Kitzmueller S, Lagler F, Moder A, Hitzl W, Wolkersdorfer M, Hofbauer P, Felder TK, Dornauer M, Diem A, Eiler N, Bauer JW. Topical diacerein for epidermolysis bullosa: a randomized controlled pilot study. *Orphanet J Rare Dis*. 8:69, 2013
- Webb NJ, Wells TG, Shahinfar S, Massaad R, Dankner WM, Lam C, Santoro EP, McCrary Sisk C, Blaustein RO. A randomized, open-label, dose-response study of losartan in hypertensive children. *Clin J Am Soc Nephrol*. 9(8):1441-8, 2014
- Wenzel D, Bayerl J, Nyström A, Bruckner-Tuderman L, Meixner A, Penninger JM. Genetically corrected iPSCs as cell therapy for recessive dystrophic epidermolysis bullosa. *Sci Transl Med* 6:264ra165, 2014.
- Wheeler JB, Ikonomidis JS, Jones JA. Connective tissue disorders and cardiovascular complications: the indomitable role of transforming growth factor-beta signaling. *Adv Exp Med Biol* 802:107-27, 2014
- Wong DL, Baker CM. Smiling faces as anchor for pain intensity scales. Pain.;89(2-3):295–300, 2010
- Zhang YE. Non-Smad pathways in TGF-beta signaling. *Cell Res* 19:128–139, 2009.

Appendices

Appendix 1 Relevant Guidelines and Laws

Declaration of Helsinki	http://www.wma.net/en/30publications/10policies/b3/
ICH- Guidelines	http://www.ich.org
EMA Guidelines	http://www.ema.europa.eu/ema/index.jsp?curl=pages/regulation/landing/human_medicines_regulatory.jsp&mid=WC0b01ac058001ff89
AMG/GCP-V	http://www.gesetze-im-internet.de
Common Terminology Criteria for Adverse Events (CTCAE) version 4.0	http://ctep.cancer.gov/protocolDevelopment/electronic_applications/ctc.htm
EB-Network	www.netzwerk-eb.de

Appendix 2 The Physician Global Assessment (PGA)

0 10

Appendix 3 The Birmingham Epidermolysis Bullosa Severity Score (BEBS)

The Birmingham Epidermolysis Bullosa Severity Score Sheet (child version)

Patient's name…………………………………… DOB…………… Type of EB……………………..
Scorer's name…………………………………………………………… Date………………………...
**See overleaf for detailed instructions*

Score item	Measure	Max	Actual score
*Nails	Lost nails ÷ 4<br>Dystrophic nails ÷ 8	5	
*Area	½ × % damaged skin: blisters, erosions, scabs, healing skin, erythema, atrophic scarring;<br>**not dyspigmentation, or well-healed scars**	50	
*Mouth	0 = no mucosal involvement	5	
*Eyes	1 = occasional blisters/erosions	5	
*Larynx	2 = frequent blisters	5	
*Oesophagus	3 = persistent symptoms, early structural abnormality<br>4 = moderate structural abnormality<br>5 = severe structural abnormality<br>(see over for detailed scoring for each site)	5	
Scarring of hands	0 = no scarring<br>1 = milia and/or atrophic scars<br>2 = just detectable contractures or webbing<br>3 = obvious contractures, or proximal webbing<br>4 = between 3 and 5<br>5 = mitten formation with fingers all fused	5	
Skin cancer (SCC)	Number of skin cancers<br>+ 1 for local/regional/lymph node spread<br>+ 2 for distant metastatic spread, up to maximum score 5	5	
Chronic wounds present for > 6/12	0 = none<br>1 = < 1% body surface area (1% = palm size)<br>2 = 1–2%<br>3 = 2–5%<br>4 = 5–10%<br>5 = > 10%	5	
Scarring alopecia due to EB	0 = no alopecia<br>1 = 1–19% scalp involvement<br>2 = 20–39%<br>3 = 40–59%<br>4 = 60–79%<br>5 = 80–100%	5	
Nutritional compromise	0–5 (where 0 = normal and 5 = cachectic)	5	
Total score		**100**	

How to fill in the BEBS score sheet

Nails: enter number in each box and add up horizontally

	R hand	L hand	R foot	L foot	Subtotals A	Subtotals B	Total score
Lost nails	+	+	+	+	=	÷ 4 =	}=
Dystrophic nails	+	+	+	+	=	÷ 8 =	
Normal nails							
Total	5	5	5	5			

Area:

Please shade in affected areas on the diagram, then work out percentage for each part and fill in the numbers in the adjacent boxes.

e.g. if half of the anterior trunk is affected, then put 9% in the box on anterior trunk.

Patient's palm size area corresponds to 1% of total body surface area.

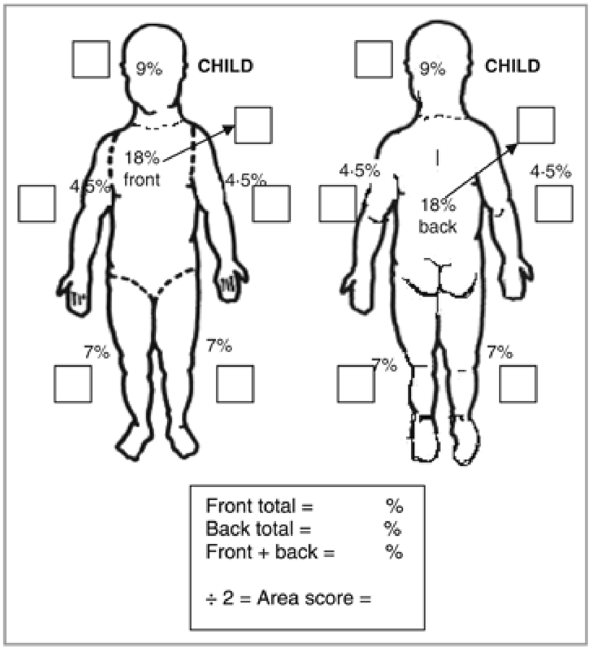

Mouth, eyes, larynx, oesophagus: detailed scoring

	Mouth	Eyes	Larynx	Oesophagus
0	No problem from EB	No problem from EB	No problem from EB	No problem from EB
1	Occasional soreness	Occasional soreness	Occasional hoarseness	Occasional dysphagia
2	Frequent soreness	Frequent soreness	Frequent hoarseness	Frequent dysphagia
3	Persistent soreness<br>Just detectable tongue tethering	Persistent soreness<br>Early visible external eye disease	Persistent hoarseness	Persistent dysphagia
4	Between 3 and 5	Between 3 and 5	Between 3 and 5	Between 3 and 5
5	Severe tongue tethering and microstomia	Bilateral sight-threatening eye disease	Life-threatening laryngeal obstruction	Difficulty swallowing solids and liquids

**C.Moss. A. Wong and P. Davies, Journal Compilation _ 2009 British Association of Dermatologists • British Journal of Dermatology 2009 160, pp1057–1065*

Appendix 4 The Epidermolysis Bullosa Activity and Scarring Index (EBDASI)

Epidermolysis Bullosa Disease Activity and Scarring Index (EBDASI)

Subject ID: ________________ EB type: _________ Date: ____/_____/_____ Rater:________________

Section I: Skin

Start time:_______________

Activity

Anatomical Location	Erosions/Blisters +/- crusting	
	0 absent<br>1 1-3 lesions, none ≥ 2 cm in any diameter<br>2 1-3 lesions, at least one lesion ≥ 2 cm in any diameter, none > 6 cm<br>3 >3 lesions, none > 6 cm in diameter<br>5 >3 lesions, and/or at least one lesion ≥ 6 cm in diameter<br>7 >3 lesions, and/or at least one lesion ≥16 cm in diameter<br>8 almost entire area involved<br>10 entire area involved	Number of lesions if < 3
Ears		
Face		
Neck		
Chest		
Abdomen		
Back		
Buttocks		
Arms		
Hands		
Legs		
Feet		
Anogenital		
**Subtotal**		
**Total skin**	**/120**	

Damage

Anatomical Location	Erythema	Post-inflammatory hyperpigmentation/ hypopigmentation (indicate colour of pigmentation)	Poikiloderma	Skin atrophy	Hyperkeratosis/ scaling(diffuse)	Scarring	Milia
	0 absent<br>1 present	0 absent<br>1 present	0 absent<br>1 present	0 absent<br>1 present	0 absent<br>1 present	0 absent<br>1 present	0 absent<br>1 present
Ears							
Face							
Neck							
Chest							
Abdomen							
Back							
Buttocks							
Arms							
Hands							
Legs							
Feet							
Anogenital							
**Subtotal**							
**Total skin**	**/84**						

Section II: Scalp

Activity

Scalp	Erosions/Blisters	Number lesions if ≤ 3
	0 absent<br>1 in one quadrant<br>2 two quadrants<br>3 three quadrants<br>4 affects four quadrants<br>8 affects four quadrants with at least one lesion > 6 cm<br><br>9 near complete scalp involvement<br><br>10 entire scalp involved	
**Subtotal**		
**Total Scalp (0-10)**	**/10**	

Damage

Post-inflammatory hyperpigmentation/ Post-inflammatory hypopigmentation or Erythema from resolving lesion or Hyperkeratosis	Scarring Alopecia
0 absent<br>1 1 quadrant involved<br>2 2 quadrants<br>3 3 quadrants<br>4 affects four quadrants<br>8 affects four quadrants with at least one lesion > 6 cm<br><br>9 near complete scalp involvement<br><br>10 entire scalp involved	0 absent<br>1 1 quadrant involved<br>2 2 quadrants<br>3 3 quadrants<br>4 affects four quadrants<br>8 affects four quadrants with at least one lesion > 6 cm<br><br>9 near complete alopecia<br><br>10 complete alopecia
/20	

Section III: Mucous Membranes

Activity

Anatomical Location	Erosions/Blisters/erythema/ mucosal atrophy/ fissures/stenosis	
	0 absent ;<br>1 1 lesion<br>2 2–3 lesions<br>5 >3 lesions or 2 lesions >2 cm<br>10 entire area	Number lesions if ≤ 3
Eyes		
Nose		
Buccal mucosa		
Hard palate		
Soft palate		
Upper gingiva		
Lower gingiva		
Tongue		
Floor of mouth		
Labial mucosa		
Posterior pharynx		
Anogenital		
**Total Mucosal Activity**	/120	

Damage

Lesions	Score (0=absent) (2=present)
**Ectropion** (inversion of eyelids)	
**Symblepharon** (fusion of conjunctival layers)	
**Visible corneal opacity**	
**Clinical microstomia** (<35mm between upper and lower incisors/alveolar processes)	
**Ankyloglossia**	
**Intraoral scars**	
**Enamel hypoplasia**	
**Anal strictures**	
**Total Mucosal Damage**	/16

Section IV: Nails

**FINGERNAILS** (please indicate the number of nails per hand affected in the respective boxes)		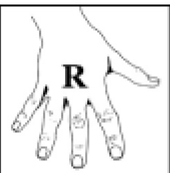	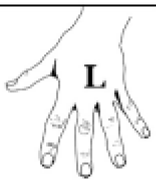	
**ACTIVITY**	blistering/ erosions/ crusting/ signs of inflammation (nail bed and nail folds)	**/5**	**/5**	Total fingernail activity score = **/10**
**DAMAGE**	Dystrophic	**/5**	**/5**	Total fingernail damage score = (no. of dystrophic nails + 3 X no. of anonychia) = **/30**
	Anonychia/ Number of amputated digits/number of digits with pseudosyndactyly	**/5**	**/5**	

**TOENAILS** (please indicate the number of nails affected in the respective boxes)		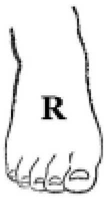	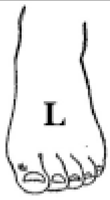	
**ACTIVITY**	blistering/ erosions/ crusting/ signs of inflammation (nail bed and nail folds)	**/5**	**/5**	Total toenail activity score = **/10**
**DAMAGE**	Dystrophic	**/5**	**/5**	Total toenail damage score = (no. of dystrophic nails + 3 X no. of anonychia) **/30**
	Anonychia/ number of amputated digits/number of digits with pseudosyndactyly	**/5**	**/5**	

**Total Nail Activity Score** = fingernail activity score + toenail activity score	**Total Nail Damage Score** = fingernail damage score + toenail damage score
**/20**	**/60**

Section V: Other epithelialized surfaces

Anatomical Location	Activity	Score	Damage	Score
**Larynx**	0 = No laryngeal involvement<br>2 = Occasional hoarseness	/2	0= no involvement<br>3 = Frequent/persistent hoarseness<br>5 = Apnoea/asphyxia episodes<br>10 = Tracheostomy	/10
**Oesophagus**	0= normal<br>2= Dysphagia	/2	0= no involvement<br>2= Stricture requiring dilatation<br>3= Recurrent stricture requiring 2-5 dilatation<br>5= Recurrent stricture requiring >5 dilatation<br>8= Insertion of nasogastric tube<br>10= Gastrostomy	/10
**Genitourinary**	0=normal<br>2= Dysuria/ Bladder spasm	/2	0= No involvement<br>3= meatal/vaginal stenosis<br>5= ureteric/urethral stenosis +/- stent<br>8= recurrent ureteric/ urethral stenosis<br>10= urostomy/PD catheter/HD catheter	/10
**Hands – pseudosyndactyly**	**NOT APPLICABLE**	**DO NOT SCORE**	0= Normal<br>1= Milia, no webbing, <25% of scarring<br>2= Milia, no webbing, 25-50% of scarring<br>3= Milia, no webbing, 50-75% of scarring<br>4= Milia, no webbing, 75-100% of scarring<br>5= Atrophic scarring(diffuse), milia, nail loss, <25% webbed<br>6= as above + 25-50% webbed<br>7= as above + 50-75% webbed<br>8= as above + 75-100% webbed<br>9= 1 hand amputated<br>10= both hands amputated	/10
**Skin Cancer(SCC)**	**NOT APPLICABLE**	**DO NOT SCORE**	0= No previous SCC<br>1=1 SCC lesion excised<br>2=2-5 SCC lesions excised<br>3=>5 SCC lesions excised<br>4= Recurrent SCC – lesions excised<br>5= Amputation of 1 limb for SCC<br>6= Amputation of >2 limbs for SCC<br>7= Metastatic SCC<br>8= Radiotherapy<br>9= Chemotherapy or targeted therapy<br>10= Death from metastatic SCC	/10
**Total score**	/6		/50	

Total Activity Score (Sections I + II + III + IV + V)	/276

Total Damage Score (Sections I + II + III + IV + V)	/230

Overall Total Score<br>*Total Activity score + Total Damage Score*	/506

Stop time: ________________ Time to complete: _____Mins _____Secs

**Lob et al., Development, reliability, and validity of a novel Epidermolysis Bullosa Disease Activity and Scarring Index (EBDASI, J Am ACAD Dermatol,2043, Vol. 70, Number 1*

Appendix 5 Hand Function Assessment Score by Colville & Terill *

Grad 0: no fusion

Grad 1: fusion extending to the proximal interphalangeal joint

Grad 2: fusion extending to the distal interphalangeal joint of finger

Grad 3: fusion extending to the tip of the digit

** Terrill PJ, Mayou BJ, Pemberton J. Experience in the surgical management of the hand in dystrophic epidermolysis bullosa. Br J Plast Surg 1992; 45: 435–442.*

Appendix 6 The Mayo Dysphagia Questionnaires-day 30 (MDQ-30)

INSTRUCTIONS: PLEASE CHECK THE APPROPRIATE BOX OR FILL IN THE BLANK AS INDICATED.

**Thank you for your willingness to participate in this important study.
Please focus on your symptoms over the past 30 days.**

1. When in your life did you first notice trouble swallowing? ______________

1 ☐ Never had trouble swallowing — **PLEASE SKIP TO QUESTION 16**
2 ☐ Less than 1 month ago
3 ☐ 1 to 3 months ago
4 ☐ 4 to 11 months ago
5 ☐ 1 to 5 years ago
6 ☐ More than 5 years ago

2. In the past 30 days, have you had trouble swallowing, not associated with other cold symptoms (such as strep throat or mono)?

1 ☐ Yes — **CONTINUE BELOW**
2 ☐ No — **PLEASE SKIP TO QUESTION 16**

3. In the past 30 days, has your swallowing trouble gotten better, remained unchanged, or gotten worse?

1 ☐ Better 2 ☐ Unchanged 3 ☐ Worse

If your swallowing trouble has changed in the past 30 days, how much has it changed?

1 ☐ Changed hardly at all
2 ☐ Change a little
3 ☐ Moderately changed
4 ☐ Changed a good bit
5 ☐ Changed very much
6 ☐ Change a great deal

4. How would you rate the severity of your trouble swallowing over the past 30 days?

1 ☐ Doesn't bother me at all
2 ☐ Mild — can be ignored if I don't think about it
3 ☐ Moderate — cannot be ignored, but does not affect my lifestyle
4 ☐ Severe — affects my lifestyle
5 ☐ Very severe — markedly affects my lifestyle
6 ☐ I don't know/remember

5. In the past 30 days, on a scale of 0 to 10, how would you rate the severity of your trouble swallowing with 0 being "Not at all severe" and 10 being "Very severe"?

0	1	2	3	4	5	6	7	8	9	10
Not at all severe										Very severe

6. How do you rate your swallowing problem today?

0	1	2	3	4	5	6
No problem						Extremely severe problem

7. How often have you had difficulty swallowing in the past 30 days?

1 ☐ None
2 ☐ Less than once a week
3 ☐ Once a week
4 ☐ Several times a week
5 ☐ Daily
6 ☐ With every meal
7 ☐ With cach swallow
8 ☐ Unable to eat

8. Do you have problems swallowing liquids?

1 ☐ Yes   2 ☐ No

If yes, is it with: (Mark only one.)

1 ☐ Cold liquids only   2 ☐ Warm liquids only   3 ☐ Both cold and warm liquids

9. Do you have problems swallowing solid foods?

1 ☐ Yes 2 ☐ No

Do you have problems swallowing liquids after solid food is stuck?

1 ☐ Yes 2 ☐ No

10. In the past 30 days, have you <u>avoided</u> any of the following types of foods to avoid food getting stuck? (Mark "Yes" or "No" for all.)

	Yes	No
**Oatmeal** (or other foods, like grits, cream of wheat, rice)	1 ☐	2 ☐
**Banana** (or other foods, like pudding, jello)	1 ☐	2 ☐
**Apple** (or other foods with fiber, such as celery)	1 ☐	2 ☐
**Ground meat** (like hamburger or ground turkey)	1 ☐	2 ☐
**Bread** (or other foods, like cake, doughnuts, muffins)	1 ☐	2 ☐
**Meat** (fibrous meats, like steak or chicken)	1 ☐	2 ☐

11. In the past 30 days, have you had <u>trouble</u> swallowing any of these foods or other foods like them? (Mark "Yes" or "No" for all.)

	Yes	No
**Oatmeal** (or other foods, like grits, cream of wheat, rice)	1 ☐	2 ☐
**Banana** (or other foods, like pudding, jello)	1 ☐	2 ☐
**Apple** (or other foods with fiber, such as celery)	1 ☐	2 ☐
**Ground meat** (like hamburger or ground turkey)	1 ☐	2 ☐
**Bread** (or other foods, like cake, doughnuts, muffins)	1 ☐	2 ☐
**Meat** (fibrous meats, like steak or chicken)	1 ☐	2 ☐

12. Do you modify your food (such as boil, use a blender, cut into small pieces, or dunk in liquid) to make it easier to swallow?

1 ☐ Yes 2 ☐ No

If yes, which of the following foods or foods like them do you modify? (Mark all that apply.)

1 ☐ Oatmeal (or other foods, like grits, cream of wheat, rice)
1 ☐ Banana (or other foods, like pudding, jello)
1 ☐ Apple (or other foods with fiber, such as celery)
1 ☐ Ground meat (like hamburger or ground turkey)
1 ☐ Bread (or other foods, like cake, doughnuts, muffins)
1 ☐ Meat (fibrous meats, like steak or chicken)

13. Compared to other people around you, in the past 30 days, at what pace do you chew during a meal?

1 ☐ At the same speed
2 ☐ A little slower
3 ☐ Much slower

14. How many minutes does it take you to eat an average meal?

1 ☐ 15 minutes
2 ☐ 16 to 30 minutes
3 ☐ 31 to 45 minutes
4 ☐ 46 to 60 minutes
5 ☐ More than 60 minutes
6 ☐ Can't eat

15. In the past 30 days, have you had pills stick in your swallowing tube?

1 ☐ Yes 2 ☐ No

16. In the past 30 days, have you had solid food (not medications) stick in your swallowing tube?

1 ☐ Yes 2 ☐ No

If yes, was it stuck for:

1 ☐ Less than 5 minutes 2 ☐ 5 minutes or more

17. In the past 30 days, when swallowing:
(Mark all that apply.)

1 ☐ It hurts all the way down
1 ☐ It hurts when food gets stuck
1 ☐ It doesn't hurt at all

18. In the past 30 days, have you experienced heartburn, a burning pain or discomfort behind the breast bone in the chest?

1 ☐ Yes 2 ☐ No

If yes, how often does this or did this heartburn occur?

1 ☐ Less than once a month
2 ☐ About once a month
3 ☐ About once a week
4 ☐ Several times a week
5 ☐ Daily

Is your heartburn better (eased) by taking antacids? (Examples: Amphojel, Alternagel, Gaviscon, Maalox, Mylanta, Riopan, Rolaids, Tums.)

1 ☐ I do not take antacids for heartburn 2 ☐ Yes 3 ☐ No

In the past 30 days, has your heartburn awakened you at night?

1 ☐ Yes 2 ☐ No

In the past 30 days, has your heartburn often traveled up toward your neck?

1 ☐ Yes 2 ☐ No

19. In the past 30 days, have you experienced acid regurgitation, a bitter or sour-tasting fluid coming up from the stomach into your mouth or throat?

1 ☐ Yes 2 ☐ No

If yes, do you experience acid regurgitation at least once a week?

1 ☐ Yes 2 ☐ No

20. Have you ever been diagnosed with seasonal or environmental (dogs, cats, cows, horses, weeds, mold, pollen) allergies?

1 ☐ Yes 2 ☐ No 3 ☐ Don't know/Not sure

21. Have you ever been diagnosed with a food allergy?

1 ☐ Yes 2 ☐ No 3 ☐ Don't know/Not sure

22. Have you ever been told by a doctor, nurse, or other health professional that you had asthma?

1 ☐ Yes 2 ☐ No 3 ☐ Don't know/Not sure 4 ☐ Refused

If yes, do you still have asthma?

1 ☐ Yes 2 ☐ No 3 ☐ Don't know/Not sure 4 ☐ Refused

The following questions ask about your use of prescription and over-the-counter medicines over the past 30 days.

23. Have you taken antacids in the past 30 days? (Examples: Amphojel, Alternagel, Gaviscon, Maalox, Mylanta, Riopan, Rolaids, Tums used as an antacid.)

1 ☐ Yes 2 ☐ No

24. Have you taken any of the following medicines in the past 30 days: Axid (nizatidine), Carafate (sucralfate), Pepcid (famotidine), Tagamet (cimetidine), Zantac (ranitidine)?

1 ☐ Yes 2 ☐ No

25. Have you taken any of the following medicines in the past 30 days: Aciphex (rabeprazole), Nexium (esomeprazole), Protonix (pantoprazole), Prevacid (lansoprazole), Prilosec, Prilosec OTC, Zegerid (omeprazole), or Kapidex?

1 ☐ Yes 2 ☐ No

26. Have you had a surgical treatment or procedure where part of your stomach was wrapped around the end of your esophagus (Nissen or Belsey fundoplication)?

1 ☐ Yes 2 ☐ No 3 ☐ Unsure

If yes, was this done in the past 30 days?

1 ☐ Yes 2 ☐ No

27. Have you had part of your esophagus removed?

1 ❑ Yes 2 ❑ No 3 ❑ Unsure

If yes, was this done in the past 30 days?

1 ❑ Yes 2 ❑ No

28. Have you had dilation (stretching) of the esophagus?

1 ❑ Yes 2 ❑ No 3 ❑ Unsure

If yes, was this done in the past 30 days?

1 ❑ Yes 2 ❑ No

Thank you for completing this questionnaire!

**Yvonne Romero et. al., The Mayo Dysphagia Questionnaire-30: Documentation of Reliability and Validity of a Tool for Interventional Trials in Adults with Esophageal Disease Dysphagia, 2010, 25;221-230*

Appendix 7 Itch assessment scale for the pediatric burn patients

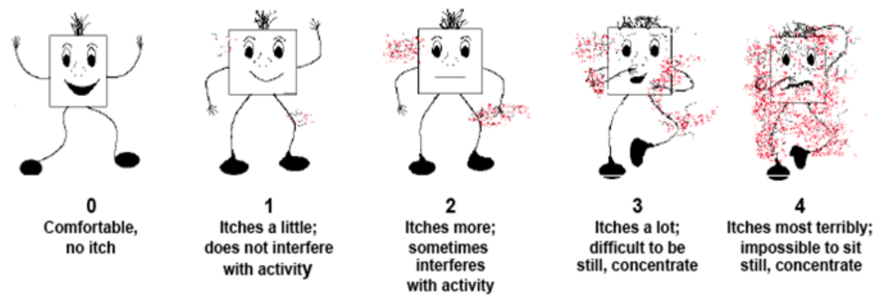

Morris et al, Itch Assessment Scale for the Pediatric Burn Survivor; J of Burn Care & Research 2012

Appendix 8 Wong Baker Faces Scale for pain

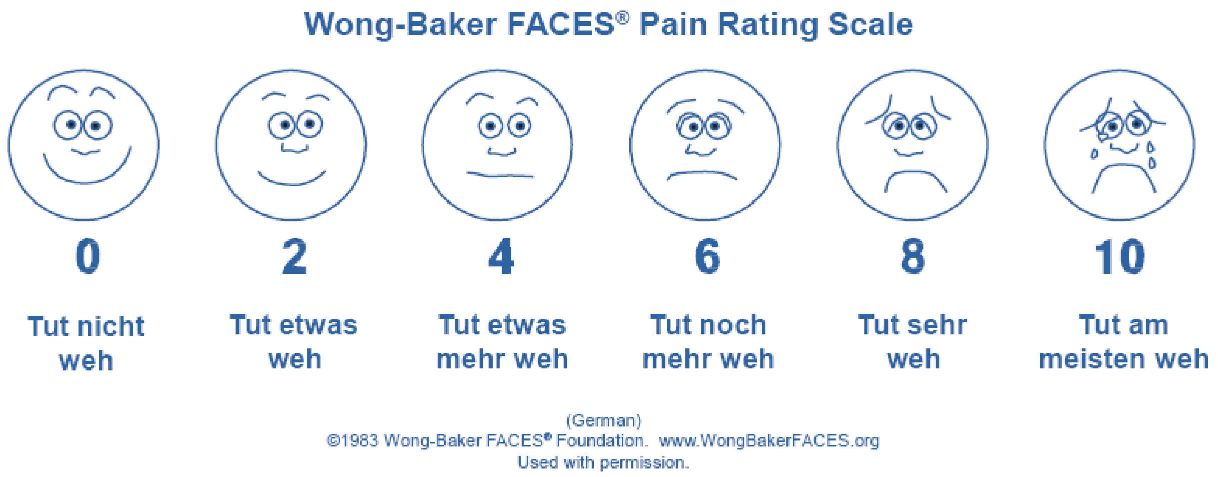

Appendix 9 QoLEB

Fragebogen zur Lebensqualität bei Epidermolysis bullosa (QOLEB)

Bitte beantworten Sie die folgenden Fragen zum Thema, wie sich die EB auf Ihr Leben auswirkt. Kreuzen Sie dabei jeweils die Antwort an, die Ihrer Situation am ehesten entspricht. Bitte geben Sie ganz unten noch an, wie lange Sie zum Ausfüllen dieses Fragebogens gebraucht haben.

1 Beeinflusst die EB Ihre Mobilität zu Hause?
- ☐ Überhaupt nicht
- ☐ Ein bisschen
- ☐ Ziemlich
- ☐ Extrem

2 Beeinflusst die EB Ihre Möglichkeiten zu baden oder zu duschen?
- ☐ Nein, überhaupt nicht
- ☐ Ja, manchmal benötige ich Hilfe
- ☐ Ja, meist benötige ich Hilfe
- ☐ Ja, ich brauche jedes Mal Hilfe beim Baden/Duschen

3 Haben Sie durch die EB körperliche Schmerzen?
- ☐ Keine Schmerzen
- ☐ Gelegentlich Schmerzen
- ☐ Häufig Schmerzen
- ☐ Ständig Schmerzen

4 Wie sehr sind Sie durch die EB beim Schreiben beeinträchtigt?
- ☐ Ich bin beim Schreiben nicht beeinträchtigt
- ☐ Ich finde es schwierig, den Stift zu halten
- ☐ Ich finde tippen leichter als schreiben
- ☐ Ich kann wegen der EB gar nicht schreiben

5 Beeinflusst die EB Ihre Fähigkeit zu essen?
- ☐ Nein, ich esse ganz normal
- ☐ Ein bisschen
- ☐ Ziemlich
- ☐ Ich benötige zur Ernährung eine Magensonde

6 Sind Sie durch die EB beim Einkaufen beeinträchtigt?
- ☐ Nein, überhaupt nicht
- ☐ Ein bisschen
- ☐ Ziemlich
- ☐ Ich brauche immer Hilfe

7 Wie wirkt sich die EB auf Ihre sportlichen Aktivitäten aus?
- ☐ Gar nicht
- ☐ Ich muss bei sportlichen Aktivitäten vorsichtig sein
- ☐ Ich muss manche sportlichen Aktivitäten vermeiden
- ☐ Ich muss alle sportlichen Aktivitäten vermeiden

8 Wie frustriert sind Sie wegen der EB?
- ☐ Überhaupt nicht
- ☐ Ein bisschen
- ☐ Ziemlich
- ☐ So sehr, dass ich fast ständig wütend bin

9 Beeinflusst die EB Ihre Mobilität außerhalb des Hauses?
- ☐ Überhaupt nicht
- ☐ Ein bisschen
- ☐ Ziemlich
- ☐ Extrem

10 Wie sehr beeinflusst die EB Ihre Beziehungen zu Familienmitgliedern?
- ☐ Überhaupt nicht
- ☐ Ein bisschen
- ☐ Ziemlich
- ☐ Extrem

11 Inwieweit schämen Sie sich vor anderen Menschen wegen der EB?
- ☐ Überhaupt nicht
- ☐ Ein bisschen
- ☐ Ziemlich
- ☐ Extrem

12 War es oder ist es aufgrund der EB notwendig, das häusliche Umfeld anzupassen oder umzubauen (Einbau von Rampen usw.)?
- ☐ Nein, überhaupt nicht
- ☐ Ein wenig
- ☐ Ziemlich
- ☐ In erheblichem Umfang

13 Wirkt sich die EB auf Ihr Verhältnis zu Ihrem Freundeskreis aus?
- ☐ Nein, überhaupt nicht
- ☐ Ein bisschen
- ☐ Ziemlich
- ☐ Meine sozialen Beziehungen sind stark eingeschränkt

14 Wie besorgt oder ängstlich sind Sie wegen der EB?
- ☐ Überhaupt nicht
- ☐ Ein bisschen
- ☐ Ziemlich
- ☐ Extrem

15 Inwieweit sind Sie oder Ihre Familie finanziell durch die EB belastet?
- ☐ Überhaupt nicht
- ☐ Ein bisschen
- ☐ Ziemlich
- ☐ Extrem

16 Wie deprimiert sind Sie wegen der EB?
- ☐ Überhaupt nicht
- ☐ Ein bisschen
- ☐ Ziemlich
- ☐ Ich bin ständig sehr deprimiert

17 Inwieweit fühlen Sie sich durch die Reaktion anderer auf Ihre EB (z. B. Hänseln und Anstarren) unwohl?
- ☐ Überhaupt nicht
- ☐ Ein bisschen
- ☐ Ziemlich
- ☐ So sehr, dass ich nicht unter Leute gehe

Wie lange haben Sie zum Ausfüllen dieses Fragebogens gebraucht?
.... Minuten

Vielen Dank.

**QOLEB-German-Version 14/2014, J Compilation, British association of dermatologists, British Journal of Dermatology, 2009 161, pp 1323-1330*

Appendix 10 Children's Dermatology Life Quality Index (CDLQI)

Ärger mit der Haut

Das Ziel des Fragebogens ist es herauszufinden, wie sehr dich dein Hautproblem **IN DER LETZTEN WOCHE** beeinflusst hat. Bitte kreuze ein Kästchen für jede Frage an.

IN DER LETZTEN WOCHE

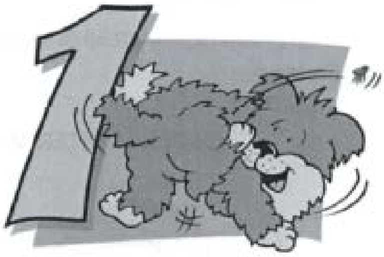

Hat deine Haut **gejuckt**, war **wund** oder hat **weh getan**?

- ☐ Sehr
- ☐ Ziemlich
- ☐ Etwas
- ☐ Gar nicht

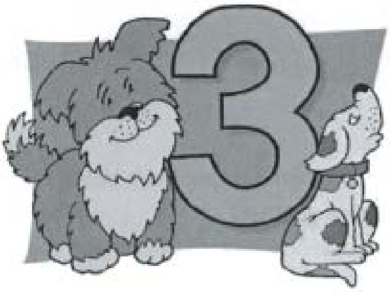

Hat dein Hautproblem deine **Freundschaften** gestört?

- ☐ Sehr
- ☐ Ziemlich
- ☐ Etwas
- ☐ Gar nicht

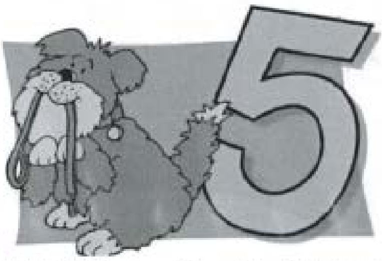

Hat dich dein Hautproblem beim **Spielen,** bei deinen **Hobbys** oder wenn du draussen etwas **unternommen** hast, gestört oder dich daran gehindert?

- ☐ Sehr
- ☐ Ziemlich
- ☐ Etwas
- ☐ Gar nicht

IN DER LETZTEN WOCHE

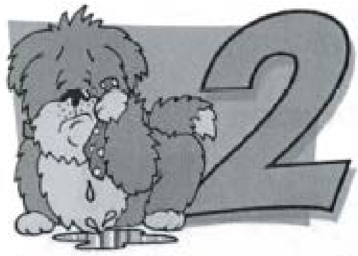

Warst du wegen deiner Haut **verlegen** oder **gehemmt, durcheinander** oder **traurig**?

- ☐ Sehr
- ☐ Ziemlich
- ☐ Etwas
- ☐ Gar nicht

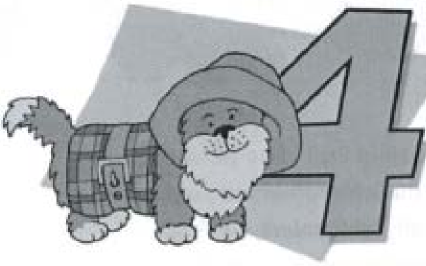

Hast du dich wegen deines Hautproblems umgezogen oder **andere** oder **besondere Kleidung/Schuhe** getragen?

- ☐ Sehr
- ☐ Ziemlich
- ☐ Etwas
- ☐ Gar nicht

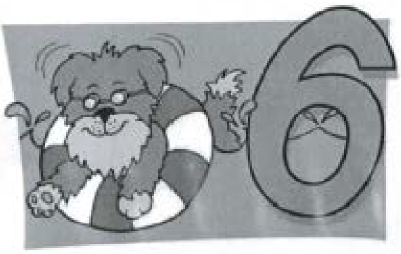

Hast du es wegen deines Hautproblems vermieden, zum **Schwimmen** oder **einem anderen Sport** zu gehen?

- ☐ Sehr
- ☐ Ziemlich
- ☐ Etwas
- ☐ Gar nicht

DERMATOLOGISCHER LEBENSQUALITÄTSFRAGEBOGEN FÜR KINDER

IN DER LETZTEN WOCHE

ENTWEDER

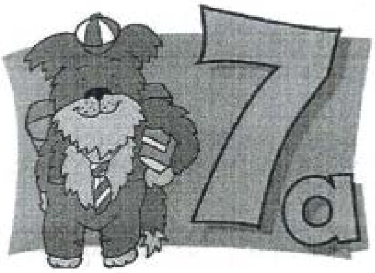

Wenn in der Schulzeit: Hat dein Hautproblem deine **Mitarbeit** in der **Schule** gestört?

Sehr ☐
Ziemlich ☐
Etwas ☐
Gar nicht ☐

ODER

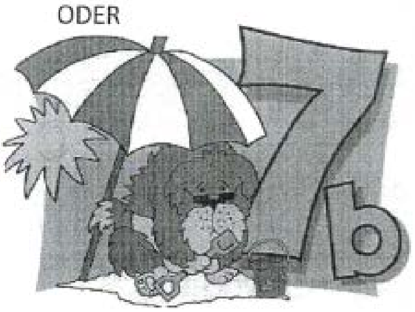

Wenn in den Ferien: Hat dein Hautproblem deinen Spaß an den **Ferien** gestört?

IN DER LETZTEN WOCHE

Sehr ☐
Ziemlich ☐
Etwas ☐
Gar nicht ☐

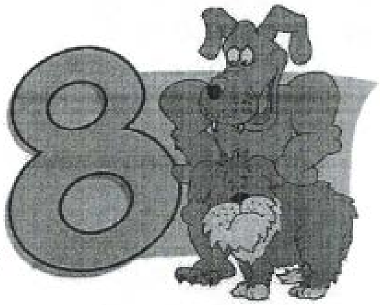

Hat dir deine Haut Probleme gemacht, weil andere **dir Schimpfnamen zugerufen, dich gehänselt, schikaniert, dir Fragen gestellt haben** oder **dich gemieden** haben?

IN DER LETZTEN WOCHE

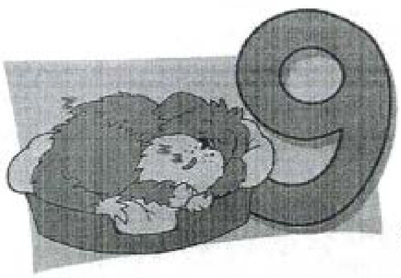

Sehr ☐
Ziemlich ☐
Etwas ☐
Gar nicht ☐

Hat dich dein Hautproblem beim **Schlafen** gestört?

Krankenhaus-Nr.
Name:
Alter:
Adresse:

Diagnose:
Datum:
CDLQI Wert:

CDLQI © M.S. Lewis-Jones, A.Y. Finlay, Juni 1993.
Illustrationen © Media Ressources Centre, UWCM. Dez. 1996

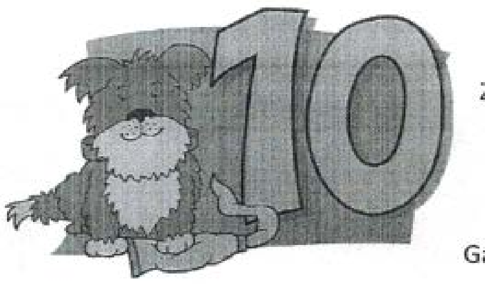

Sehr ☐
Ziemlich ☐
Etwas ☐
Gar nicht ☐

Hat die **Behandlung** deiner Haut dir Probleme gemacht?

Bitte schau nach, ob du JEDE Frage beantwortet hast. Vielen Dank.

**CDLQI M.S.Lewis-Jones, A.Y.Finlay, 1993   Illustration:Media Ressources Center, UWCM,1996*

Appendix 11 Exacerbating factors

1. Leistungsfähigkeit	□ normal	□ gesteigert	□ reduziert
2. Schlafbedürfnis	□ normal	□ gesteigert	□ reduziert
3. Nervosität/Anspannung	□ normal	□ gesteigert	□ reduziert
4. Bewegung/ Mobilität	□ normal	□ gesteigert	□ reduziert
5. Appetit	□ normal	□ gesteigert	□ reduziert
6. Übelkeit	□ normal	□ gesteigert	□ reduziert
7. Vermehrtes Weinen / Quengeln	□ normal	□ gesteigert	□ reduziert
8. Weitere:________________________	□ normal	□ gesteigert	□ reduziert

Appendix 12 Morphometric pseudosyndactyly scoring

General information

We will develop a specific morphometric scoring instrument of pseudosyndactyly progression in RDEB using two approaches.

First, in analogy to the score used in the RDEB mouse model volar, dorsal and lateral photographs of both hands will be taken, along with a centimeter scale. Using Image J software, the length of individual fingers will be measured from the fingertip to the point of fusion with adjacent fingers. As the patients will grow during the treatment period, the measurements will be adjusted to the length of the underarm and the body height as internal reference.

Second, the hand contours will be drawn by a perpendicular pen, and the shadow area will be determined. Also this area will be correlated to the length of the underarm and the body height. For inter-individual comparison of the scores, values will be expressed as % change of the baseline measurement.

Central data assessment of hand images collected from all patients take place in Freiburg. For details see laboratory manual.

Appendix 13 Fibrotic and inflammatory markers in skin biopsy specimens

Biopsy procedure

A 4-mm punch biopsy will be taken from clinically unaffected skin, 1-2 cm from the border of scarred/fibrotic area at a similar site on the hands or feet, in order to increase reproducibility and to minimize variation arising from acute blistering-associated inflammation. The biopsies will be fixed in formalin immediately after collection, stored and then send to Freiburg to be processed for (immuno)histological analysis (for details refer to the laboratory manual).

Biopsy sample processing

First, collagen VII will be stained to ensure that the biopsied area does not represent a revertant mosaic spot.

Second, TGFβ activity will be assessed by staining for TGFβ protein, TGFβ receptor II and pSMAD2/3.

Inflammation and immune reactions will be determined by staining with antibodies to CD3 (mature T-cells), CD4 (mainly T-helper cells), CD8 (mainly cytotoxic T-cells), CD68 (monocytes/macrophages), TNFα and IL6.

Histological stains (H&E, picrosirus red) will reveal the extent of fibrotic processes.

At least two skin sections will be stained for each marker, images captured and the intensity of the signals assessed with Image J software.

Central sample processing and analysis will take place in Freiburg. For details see laboratory manual.

Appendix 14 The fibrotic and inflammatory markers in serum

The central analyses of collected blood samples will include TGFβ levels, and biomarkers of inflammation and fibrosis, such as TGFβ, TNFα, IL6 and amyloid.

TGFβ and TNFα will be analyzed by specific quantitative ELISAs (RayBiotech) at the EB Center Freiburg, IL6 and amyloid are measured at the central laboratories of the Medical Centers in Freiburg and Salzburg.

In addition, the sera will be analyzed for procollagen peptides PINP and PIIINP by ELISA (Uscn Life Science Inc.) at the EB Center Freiburg.

Central sample processing and analysis will take place in Freiburg. For details see laboratory manual.
